# Impact of G‐CSF on Donor TCR Clonal Diversity and T Cell Function During Donor HSC Mobilisation

**DOI:** 10.1111/cpr.70213

**Published:** 2026-04-16

**Authors:** Xinye Li, Zhixi Chen, Jie Chen, Xingwei Zhang, Jiamian Zheng, Zirui Liu, Meixue Pan, Yue Li, Bo Yu, Shaohua Chen, Jing Lai, Xianfeng Zha, Liye Zhong, Yikai Zhang, Yangqiu Li

**Affiliations:** ^1^ State Key Laboratory of Bioactive Molecules and Druggability Assessment, Guangdong Basic Research Center of Excellence for Natural Bioactive Molecules and Discovery of Innovative Drugs, Key Laboratory for Regenerative Medicine, Ministry of Education, Institute of Hematology, School of Medicine Jinan University Guangzhou China; ^2^ Department of Hematology First Affiliated Hospital of Jinan University Guangzhou China; ^3^ Department of Clinical Laboratory First Affiliated Hospital of Jinan University Guangzhou China; ^4^ Medical Experiment Research Center School of Medicine, Jinan University Guangzhou China; ^5^ Experimental Teaching Center of Functional Science of Basic Medicine, School of Medicine Jinan University Guangzhou China

**Keywords:** allogeneic haematopoietic stem cell transplantation, graft‐versus‐host disease, recombinant granulocyte colony‐stimulating factor, T cell receptor, T cells

## Abstract

Recombinant granulocyte colony‐stimulating factor (G‐CSF) is widely used to mobilise donor stem cells into peripheral blood for allogeneic haematopoietic stem cell transplantation (allo‐HSCT). Studies have shown that G‐CSF may reduce the incidence of acute graft‐versus‐host disease (aGVHD) following allo‐HSCT by modulating T cell function. However, the patterns and mechanisms by which G‐CSF regulates T cell function remain unclear. In this study, we used RNA sequencing combined with T cell receptor (TCR) immune repertoire sequencing to discover that G‐CSF mobilisation leads to a reduction in donor TCR clonal diversity, downregulation of genes associated with TCR recombination, suppression of multiple antigen presentation processes, and varying degrees of downregulation in multiple T cell function‐related signalling pathways. The results suggest that the pathway by which G‐CSF mediates a low incidence of GVHD after allo‐HSCT is by interfering with donor TCR recombination and antigen presentation processes, resulting in suppression of multiple T cell functions.

## Graphical abstract

In this study, we found that G‐CSF mobilisation caused a reduction in TCR clonal diversity and down‐regulation of T‐cell function in donors. This may be one of the reasons for the low incidence of GVHD mediated by G‐CSF after allo‐HSCT. *Source:* Created in BioRender. Li https://BioRender.com/otownki.

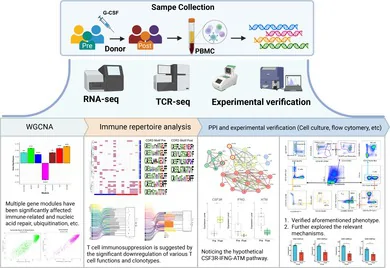

## 1 Introduction

Granulocyte colony‐stimulating factor (G‐CSF) is a widely used mobilising agent in haematopoietic stem cell transplantation. This glycoprotein can induce the production of mature neutrophils, promote the proliferation and differentiation of haematopoietic stem and progenitor cells (HSPCs), and facilitate the release of HSPCs from the bone marrow into peripheral blood [1, 2, 3, 4, 5]. Research has indicated that G‐CSF mobilises donor early myeloid‐derived suppressor cells (eMDSCs) with immunosuppressive potential, which can inhibit recipient acute graft‐versus‐host disease (aGVHD) [6]. Recently, studies have demonstrated that G‐CSF has immunomodulatory effects on both innate and adaptive immune cells [7, 8, 9]. G‐CSF can induce T cell tolerance by increasing the quantity and function of regulatory T cells (Tregs) [10, 11, 12], regulating cytokine expression, inhibiting T cell proliferation, and maintaining T cell immune hyporesponsiveness [13, 14, 15]. However, G‐CSF does not seem to influence the graft versus leukaemia (GVL) function [16, 17, 18]. Previous studies have also reported temporary immunosuppression in donors following G‐CSF mobilisation treatment [19].

The T cell receptor (TCR) is a crucial component for T cell antigen recognition, and its diversity largely determines the potential of T cells to recognise various antigens or antigen epitopes. TCR repertoire sequencing (TCR‐seq) is widely used to assess TCR diversity and to screen for highly selected TCR clones. The TCR rearrangement process primarily involves DNA double‐strand breaks and recombination repair of TCR gene ends through non‐homologous end joining (NHEJ) [20, 21, 22]. Numerous genes related to TCR rearrangement [13, 20, 23, 24, 25, 26, 27, 28, 29, 30, 31] and NHEJ [32] have been identified. Studies have also reported that the TCR α and δ enhancers [29, 33, 34, 35, 36], MYB proto‐oncogene (*c‐Myb*) [33, 34, 37], and core‐binding factor subunit beta (*CBF/PEBP2*) [34, 38] are involved in the TCR rearrangement process. Additionally, various transcription factors such as GATA binding protein 3 (*GATA3*), signal transducer and activator of transcription 5 (*STAT5*) [39], RUNX family transcription factor 1 (*RUNX1*)/ *ETS* proto‐oncogene 1 (*ETS1*) [27], and *MYB*/*RUNX1* [28, 29, 40, 41], participate in regulating TCR rearrangement. Thus, any alteration in the different stages of TCR rearrangement or in the genes involved could affect TCR diversity and the formation of antigen‐specific TCR clones. In contrast, alteration of TCR diversity in peripheral blood T cells also indicates a predominant usage of the TCR repertoire in different contexts.

In this study, we utilised transcriptome sequencing (RNA‐seq) combined with TCR‐seq and bioinformatic analysis to examine changes in the donor transcriptome and TCR clonal diversity before and after G‐CSF mobilisation. We discovered that G‐CSF treatment markedly downregulates donor TCR diversity, suppresses the expression of genes involved in TCR rearrangement, and inhibits biological behaviours related to T cell function. The high‐frequency TCR rearrangement patterns in donors were notably altered following G‐CSF mobilisation, suggesting that G‐CSF treatment might induce changes in the types or epitopes of antigens recognised by donor T cells. Furthermore, G‐CSF treatment inhibited multiple antigen presentation biological processes in the donors. These findings will further enrich our understanding of the mechanisms of T cell immune tolerance after allogeneic haematopoietic stem cell transplantation (allo‐HSCT) induced by G‐CSF.

## 2 Materials and Methods

### 2.1 Clinical Sample Collection

The donors involved in this study, who were preparing to provide haematopoietic stem cells for patients undergoing allo‐HSCT, were from the Department of Haematology at the First Affiliated Hospital of Jinan University (*n* = 8, pair‐wise). The pre‐mobilisation samples were the donors' peripheral blood (PB) without treatment with recombinant human granulocyte colony‐stimulating factor injection (Xiamen Tebao Biological Engineering Co., China). The post‐mobilisation samples were the donors' PB collected on the morning of the fifth day after treatment with 5 μg/(kg·12 h) G‐CSF injection nine times. The PB samples collected were residual samples remaining after the donors completed necessary clinical tests. The donors provided informed consent, and this study was approved by the Ethics Committee of the First Affiliated Hospital of Jinan University (20220915).

### 2.2 Peripheral Blood Mononuclear Cells Extraction

Peripheral blood mononuclear cells (PBMCs) were isolated and extracted from collected donor or healthy volunteers' PB samples using Ficoll–Hypaque gradient centrifugation following previously described methods [42, 43]. After extraction, the donors' PBMCs were fully lysed with TRIzol (Invitrogen, Thermo Fisher Scientific) and stored at −80°C for subsequent RNA extraction. The healthy volunteers' PBMCs were used for the T cells' enrichment, culture, and G‐CSF treatment.

### 2.3 T Cell Enrichment, Culture, and Treatment

The CD3^+^ T cells for culture and G‐CSF treatment in vitro were enriched from the PBMCs of healthy volunteers (*n* = 4). PBMCs were isolated as described in 2.2 above. The enrichment and culture of CD3^+^ T cells were performed as described in our previous study [44]. The cells were expanded for 4 days after activation by T cell TransAct (Miltenyi Biotec, Germany) and then used for G‐CSF (Xiamen Tebao Biological Engineering Co., China) treatment. The cells were treated with different concentrations of G‐CSF (10, 50, and 100 ng/mL) [45, 46, 47] for 4 days, and then were collected for flow cytometry, RNA sequencing (RNA‐seq), TCR repertoire sequencing (TCR‐seq), and quantitative PCR (qPCR).

### 2.4 Flow Cytometry Analysis

Treated human CD3^+^ T cells were collected. The treatment and antibody staining were performed as reported in our previous study [44]. The cultured samples were stained with CD3‐FITC, CD8‐PerCP‐Cy5.5, CCR7‐PE, CD45RA‐APC and CD69‐PE‐Cy7. The donors' PB samples before and after G‐CSF mobilisation were lysed with red‐cell lysates, and then stained with CD3‐Spark blue 550, CD4‐cFluor BYG750, CD8‐PerCP‐Cy5.5, CCR7‐PE‐Fire810, CD45RA‐BV510, CD16‐Pacific blue, CD56‐APC‐R700, CD127‐Spark NIR 685, CD25‐PE‐Fire 640, CD14‐BV605, CD141‐BV785, CD11c‐APC, CD19‐APC‐Cy7 and HLA‐DR‐BV711. Flow cytometric analysis of the culture samples was performed using a CytoFLEX (Beckman Coulter, USA) flow cytometer, while the donors' samples (*n* = 6, pair‐wise) were analysed using a Cytek Aurora 3 Laser (Cytek, USA). All data were analysed using FlowJo software.

### 2.5 RNA Extraction and qPCR Analysis

Total RNA was extracted from the donors' PBMCs before and after G‐CSF mobilisation as well as the G‐CSF‐treated CD3^+^ T cells in vitro using TRIzol following the detailed procedure described in our previous study [48, 49]. The extracted RNA was stored at −80°C for subsequent TCR repertoire sequencing, RNA‐seq and qPCR. cDNA libraries were prepared from the total RNA with a reverse transcription kit (Invitrogen, USA). The oligonucleotide sequences are provided in Table S1. ACTB was used as an internal control.

### 2.6 TCR Repertoire Sequencing

The TCR‐seq was performed on donors' PBMCs before and after G‐CSF mobilisation or the G‐CSF‐treated CD3^+^ T cells in vitro. A portion of the extracted RNA was used to amplify immune library sequences using the 5′ Rapid Amplification of cDNA Ends (RACE) technique. After amplification, the concentration and integrity of the fragments were assessed using Qubit, Agilent and qPCR. Qualified libraries were sequenced using the HiSeq or MiSeq platforms. TCR gene amplification and sequencing were performed by *Guangzhou Huayin Medical Laboratory Center*. After passing quality control, upstream analysis was completed using the MixCR software [50, 51] (v4.5.0), retaining complementarity‐determining region 3 (CDR3) sequences with greater than four amino acids, nucleic acid lengths in multiples of three, and no stop codons as qualified clones for downstream bioinformatics analysis. Subsequent analysis was conducted using the R software (R4.3.2) immunarch package (v0.9.0, CRAN https://cran.r‐project.org/). Multiple statistical analysis methods were adopted, including Clonotype, Chao1, and D50. All of these analysis methods are based on the number of clonotypes (not counts) and the immunarch analysis pipeline. The original sequencing data were first normalised through immunarch algorithms before being used for subsequent statistical analysis. MotifStack [52] was used to create CDR3 amino acid motif plots. We also used TCRdist3 [53, 54] (v0.1.0) for meta‐clonotype identification and analysis. An empirical cumulative distribution function (ECDF) was constructed for each unique TCR, showing the proportion of all TCRs within a specified radius. The OLGA algorithm [55] was employed, generating synthetic background TCR arrays from 100,000 OLGA‐generated TCRs and 100,000 TCRs subsampled from cord blood [56, 57], and TCRdist3 arrays were constructed by selecting top‐ranked clones in the Pre and Post groups.

### 2.7 RNA‐seq and Statistical Analysis

An aliquot of RNA extracted from donors' PBMCs before and after G‐CSF mobilisation was used for library preparation and bulk RNA‐seq using the VAHTS Universal DNA Library Prep Kit for Illumina V3. After passing quality control, the raw data were analysed using STAR (v2.7.11a). Further processing and counting were conducted using Picard (v3.1.1) and featureCounts (subread, v2.0.6). Subsequently, we first explored the relationship between gene expression and clinical conditions using weighted gene co‐expression network analysis (WGCNA) [58] (v1.72 CRAN https://cran.r‐project.org/) and then predicted immune infiltration using CIBERSORT, MCPCOUNTER, and QuantiSEQ (https://compbio.cn/timer3/) [59, 60, 61, 62]. Further analysis and visualisation of the results were performed using R software (R4.3.3). Differential expression analysis was conducted using EdgeR (v4.4.2) [63] (Log2FC > 2 or Log2FC < −2, *p* < 0.05) with enrichment analysis performed using Gene Ontology (GO) [64] and Kyoto Encyclopaedia of Genes and Genomes (KEGG). Finally, gene set enrichment analysis (GSEA) was conducted using clusterProfiler (v4.14.6) [65]. To ensure reproducibility, the same random seed was set for each experiment (R: set. seed, seed = 233). An aliquot of the RNA extracted from the CD3^+^ T cells treated by G‐CSF in vitro was also used for library preparation and bulk RNA‐seq using the VAHTS Universal DNA Library Prep Kit for Illumina V3. Subsequent procedures and codes for the quality control, raw data processing, the differential expression analysis, the GO analysis, KEGG analysis, and the GSEA analysis were the same as those described above for analysing data from donors' PBMCs before and after G‐CSF mobilisation. The EdgeR, clusterProfiler, ComplexHeatmap (v2.22.0) and motifStack (v4.16.0) were installed via Bioconductor (http://bioconductor.org/). All statistical analyses were performed using R4.1.0 and GraphPad Prism 10 software, including *t*‐test, Wilcoxon rank‐sum test and Kruskal–Wallis test. All significance levels were indicated by *p*‐values, with *p* < 0.05 considered statistically significant (****p < 0.0001, ***p < 0.001, **p < 0.01, * *p* < 0.05, ns: not significant).

## 3 Results

### 3.1 G‐CSF Treatment Affects Donors' T Cell Function

**FIGURE 1:**
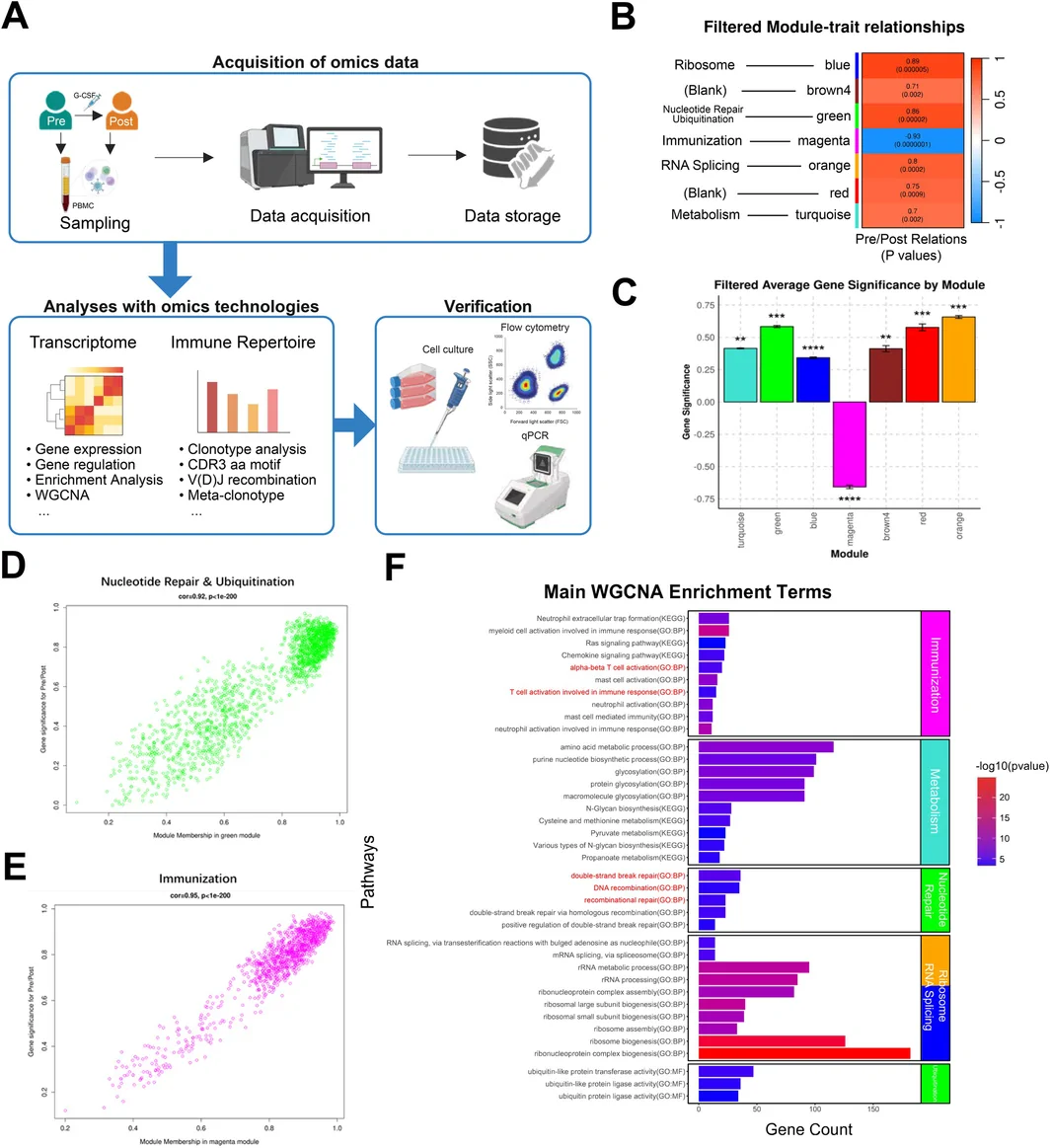
Correlation between Trait‐Related modules and gene significance by WGCNA before and after G‐CSF treatment. (A) Flow chart of this study. (B) Correlation between WGCNA modules and traits. Shown are correlations between different WGCNA modules and traits. (C) Average gene significance of each WGCNA module. Displayed is the average significance of the genes within each module. (D, E) Scatter plots of trait‐related modules and gene significance for the most significant green and magenta modules. Genes highly correlated with the module (upper right) are also highly correlated with the trait; genes with low module correlation (lower left) are also not related to the trait. These plots show a high correlation between gene significance and module membership. (F) General enrichment analysis results for major module genes. Statistical methods: (B‐E) Pearson correlation. (F) Hypergeometric test. *p*‐values were adjusted by the Benjamini–Hochberg method. The stars refer to the *p*‐value markers. **** = *p* < 0.0001, *** = *p* < 0.001, ** = *p* < 0.01. *Source:* Created in BioRender. Li https://BioRender.com/r5jty4e.

RNA‐seq combined with TCR‐seq was used to examine transcriptomic changes and TCR clonal diversity in donor PBMCs before and after G‐CSF mobilisation. qPCR was then used to verify the expression level of genes of interest in donor PBMCs before and after G‐CSF mobilisation (Figure 1A).

**FIGURE 2:**
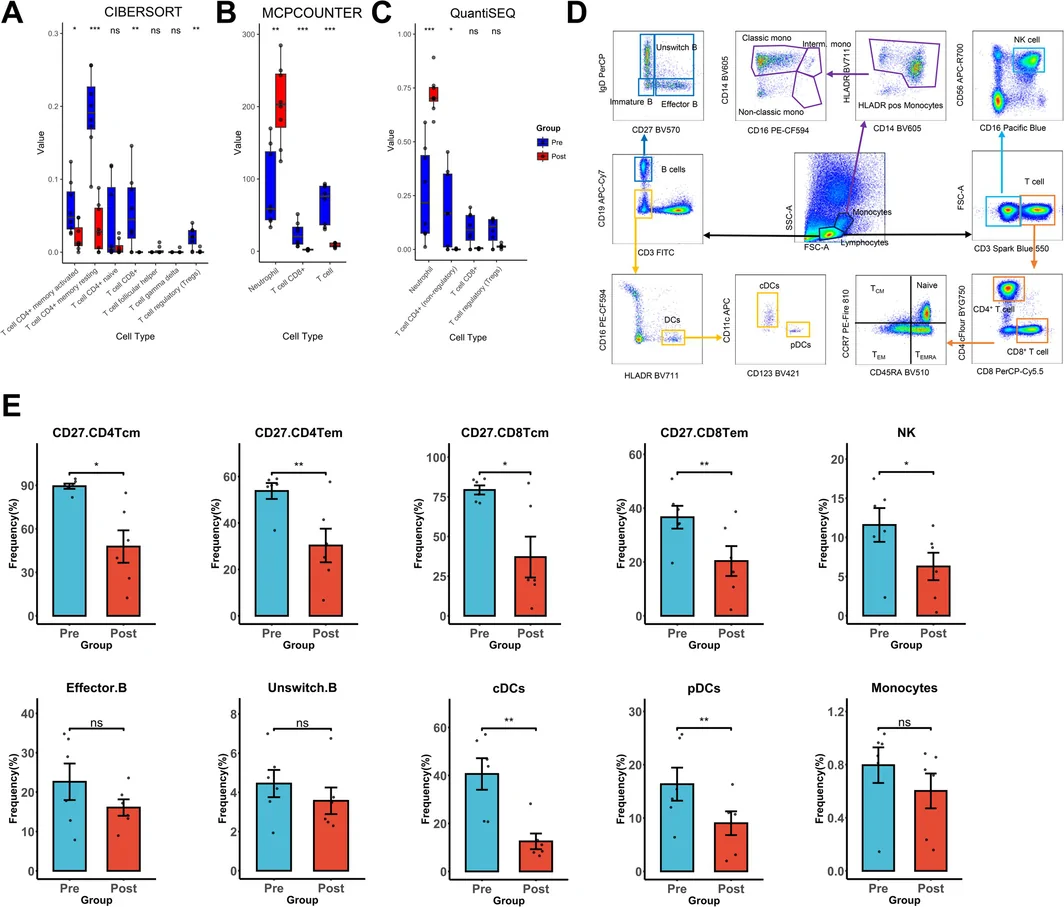
G‐CSF treatment affects the distribution of donors' T cell subsets. Changes in the proportions of T cells before and after donor mobilisation predicted by CIBERSORT (A), MCPCOUNTER (B) and QuantiSEQ (C). The proportion of different immune cells in donors' PB before and after G‐CSF mobilisation was detected by multicolour flow cytometry. (D) Gating strategies for the cell populations. (E) Proportions of various immune cells. Statistical methods: (A–C) Wilcoxon rank‐sum test. (E) Paired sample *t*‐test. The stars refer to the *p*‐value markers., ** = *p* < 0.01, * = *p* < 0.05, ns = *p* > 0.05.

Using WGCNA, seven gene modules strongly correlated with the pre‐ and post‐mobilisation states (*p* < 0.01, *r* > 0.7, Pearson correlation; Figure 1B–E, Figure S1A–F), and there was clustering of the genome‐wide expression patterns in donor PBMCs before and after G‐CSF mobilisation [58, 66]. GO/KEGG enrichment analysis revealed that these modules were primarily annotated in six categories: ribosomal behaviour, nucleic acid repair, ubiquitination, RNA splicing, immunity and metabolism (Figure 1F). Among these, immunity (purple gene module, Figure 1E) and nucleic acid repair and ubiquitination (green gene module, Figure 1D) demonstrated the most significant differences. These data suggest that G‐CSF treatment may influence processes such as nucleic acid repair and ubiquitination, as well as cellular metabolism and immune cell function. We next used three immune cell infiltration analysis algorithms, CIBERSORT, MCPCOUNTER and QuantiSEQ [59, 60, 61, 62], to predict the proportion of various immune cell types before and after G‐CSF mobilisation (Figure 2A–C, Figure S1G–I). The results indicated a significant increase in the proportion of neutrophils following G‐CSF treatment, while other immune cells, including CD4^+^ T cells, CD8^+^ T cells, B cells, natural killer cells (NK cells), dendritic cells (DCs) and eosinophils, exhibited a decrease. In addition, multicolour flow cytometry also demonstrated that the proportion of different T cell subsets, B cells, DCs and NK cells decreased after G‐CSF mobilisation (Figure 2D,E), which was consistent with the results of immune cell infiltration analysis. Furthermore, we also treated the human CD3^+^ T cells with G‐CSF in vitro. The flow cytometry results showed that the proportion of activated CD4^+^ memory T cells (Act CD4^+^ TM) and resting CD4^+^ memory T cells (Res CD4^+^ TM) decreased after G‐CSF treatment. These results showed a trend similar to that of the immune infiltration analysis. However, there was no significant alteration in the proportion of CD8^+^ T cells after G‐CSF treatment in vitro (Figure S2A–C).

**FIGURE 3:**
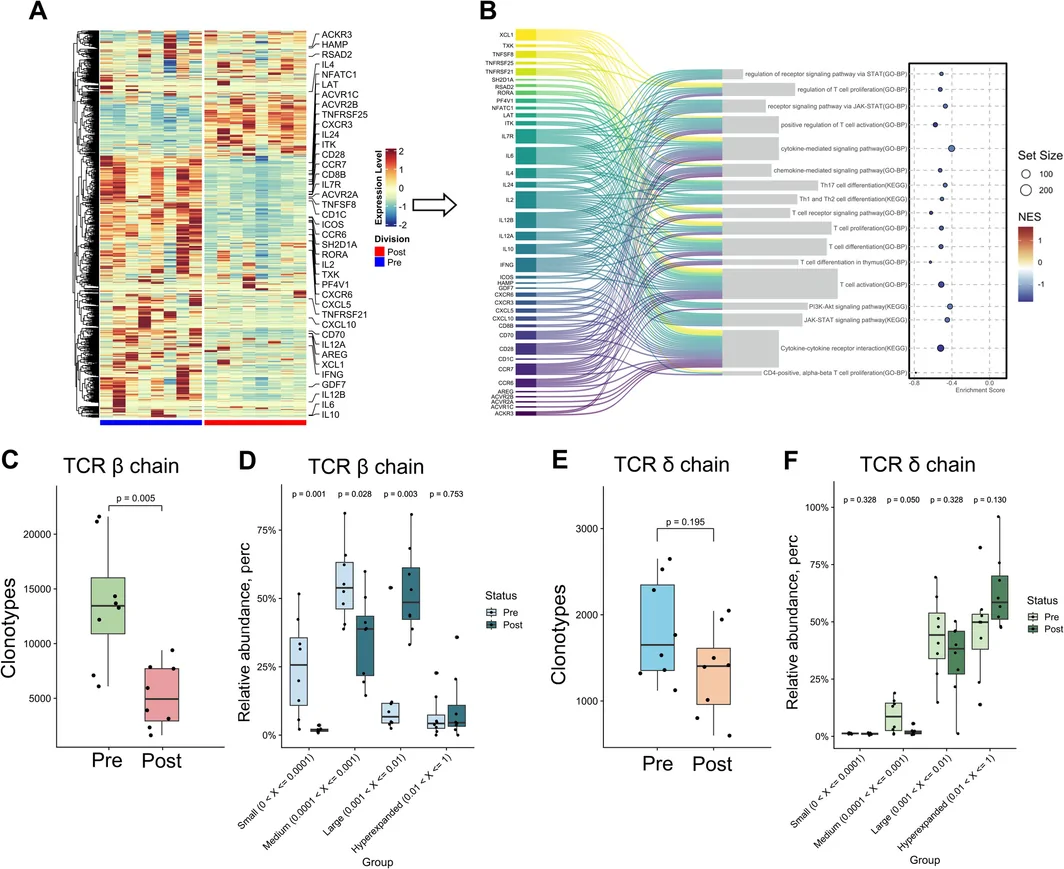
G‐CSF treatment inhibits donor T cell function. (A) Heatmap of differentially expressed T cell function genes before and after G‐CSF mobilisation. (B) GSEA results of T cell function‐related genes before and after G‐CSF mobilisation. Displayed are the GSEA results for the genes related to T cell function before and after G‐CSF mobilisation. (C) Absolute count of donor TCR Vβ chain clones before and after G‐CSF mobilisation. (D) Spatial stability analysis of donor TCR Vβ chain clones before and after G‐CSF mobilisation. (E) Absolute count of donor TCR Vδ chain clones before and after G‐CSF mobilisation. (F) The spatial stability of the TCR Vδ chain clones in donors before and after G‐CSF mobilisation is shown. Statistical methods: (C–F) Wilcoxon rank‐sum test.

Previous studies have reported that G‐CSF can modulate T cell immune function [9, 19], and the WGCNA results indicated that G‐CSF treatment could significantly alter the expression of genes highly associated with immunity. Therefore, by interrogating our RNA‐seq data using the ImmPort database [67], we further found that G‐CSF treatment could downregulate various T cell biological processes, including T cell activation, proliferation and differentiation. Several T cell function‐related signalling pathways, such as the TCR signalling pathway (KEGG: hsa04660, GO:0050852), the PI3K‐AKT signalling pathway (KEGG: hsa04151) and the JAK–STAT signalling pathway (KEGG: hsa04630, GO:0007259), were also inhibited following G‐CSF treatment (Figure 3A,B). These findings suggest that G‐CSF treatment may suppress donor T cell function.

### 3.2 Donor TCR Diversity Significantly Decreases After G‐CSF Mobilisation

TCR diversity (also known as richness) is fundamental to the capacity of T cells to recognise antigens, playing a critical role in their immune function. Thus, assessing TCR diversity can provide insight into the immune status of T cells in subjects [68, 69, 70, 71]. We used TCR‐seq to examine changes in donor TCR diversity before and after G‐CSF mobilisation. Initially, we analysed the frequency differences in the usage of the separate V, D and J gene segments in the TCR β and TCR δ chains before (Pre) and after (Post) G‐CSF mobilisation. Interestingly, there were no significant differences in the high‐frequency usage of the separate V, D and J gene segments in the TCR β chain between the Pre and Post groups (Figure S3), or in the TCR δ chain between the Pre and Post groups (Figure S4). We subsequently quantified the absolute counts of TCR clones in both groups and performed D50 and Chao1 statistical analyses. The absolute counts of TCR β chain clones significantly decreased in the Post group compared to the Pre group (*p* = 0.005, Wilcoxon rank‐sum test) with individual reductions ranging from 15% to 70% (Figure 3 C, D). The D50 and Chao1 analyses also demonstrated a significant decrease in the Post group compared to the Pre group (Figure S5A,B). For the TCR δ chain clones, the absolute counts, D50 and Chao1 analyses exhibited less pronounced reduction with a downward trend in the Post group relative to the Pre group (Figure 3E,F and Figure S5C,D). Moreover, further clonal space homeostasis analysis revealed a significant decrease in the proportion of small and medium clones observed for the TCR β chain in the Post group, indicating a marked reduction in TCR β chain clonal diversity. Whereas only a decreasing trend in medium clones was detected for the TCR δ chain in the Post group (Figure 3D,F). Additionally, clone index distribution analysis demonstrated a substantial increase in the proportion of clones at [11: 100) and [101: 1000) and a substantial decrease in the proportion of clones at index levels of [1001: 3000) and [3001: 10000) in the Post group for the TCR β chain (Figure S5E). This indicated that the high‐frequency TCR β chain clones were increased while the low‐frequency TCR β chain clones were reduced, which suggested the reduction of TCR β chain clonal diversity. Whereas only an increasing trend in the proportion of clones at [1: 10) and [11: 100) and a decreasing trend in the proportion of clones at [101: 1000) in the Post group for the TCR δ chain were found (Figure S5F). Overall, these results suggest that G‐CSF treatment induces a reduction in the clonal diversity of donor TCR β and δ chains, potentially leading to a decreased capacity of donor T cells to recognise antigens following G‐CSF mobilisation.

### 3.3 Alterations in the High‐Frequency Utilisation of TCR Clonotypes in Donor T Cells After G‐CSF Mobilisation

It is known that the frequency of TCR clonotype utilisation reflects the dominance of antigen‐specific T cell clonal expansion [72, 73]. In this study, we found that the TOP 20 high‐frequency TCR Vβ clonotypes in the Pre group were almost absent in the high‐frequency clonotypes in the Post group. Similarly, the TOP 20 high‐frequency TCR Vβ clonotypes in the Post group were expressed at very low levels or simply not expressed in the Pre group (Figures S6A, S7), indicating significant differences in high‐frequency TCR Vβ clonotype utilisation before and after G‐CSF mobilisation. A similar trend was observed for the high‐frequency TCR Vδ clonotypes, where those highly expressed in the Pre group were either expressed at a low level or unexpressed in the Post group or vice versa (Figures S6B, S8). These results suggest that G‐CSF treatment induces significant alterations in high‐frequency TCR clonotype utilisation, implying that G‐CSF treatment may result in changes in dominant expanded T cell clones in donors.

**FIGURE 4:**
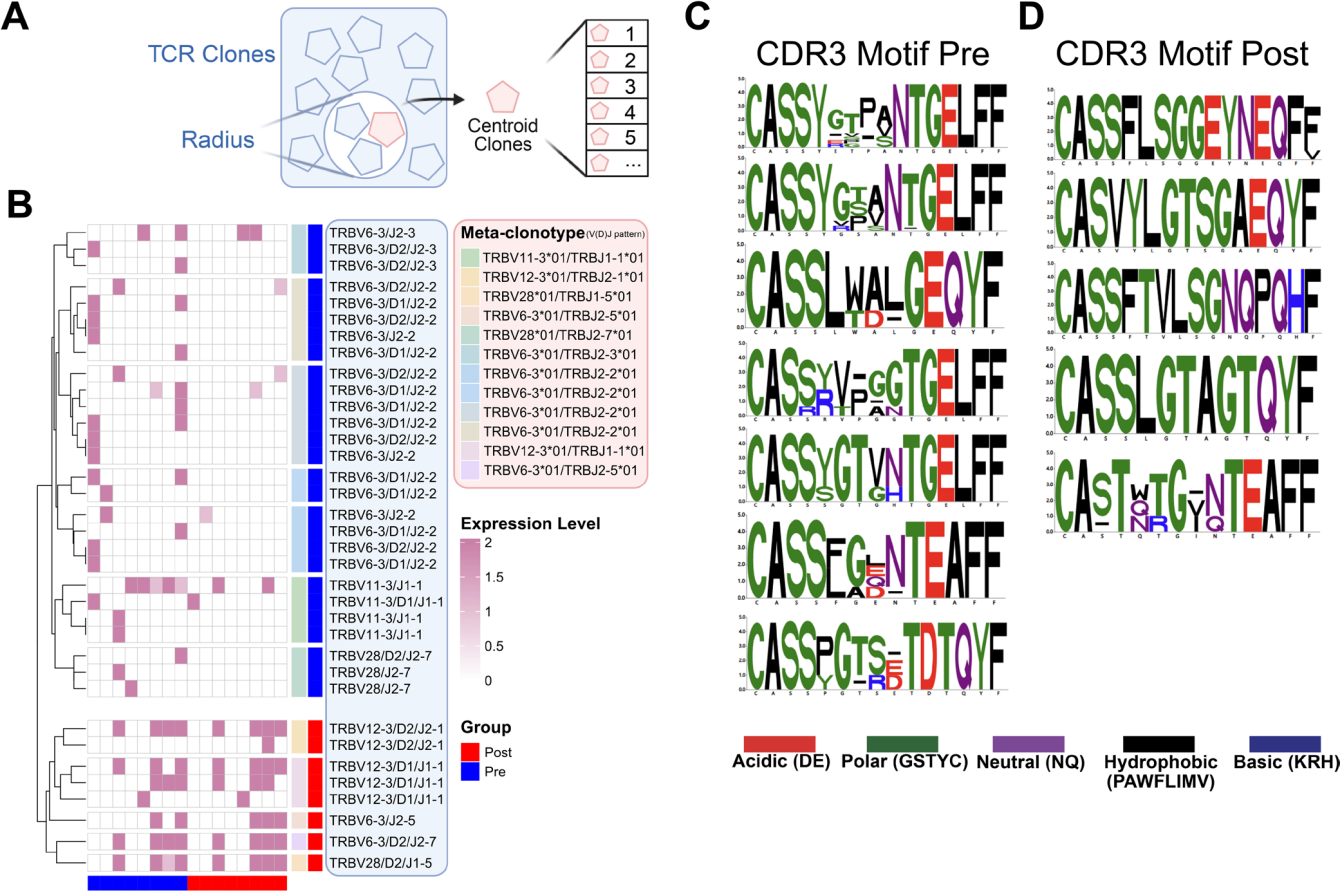
Changes in the TCR Vβ chain clonotype distribution before and after G‐CSF mobilisation. (A) Work‐flow of the meta‐clonotype analysis. (B) The connection between Pre/Post TCR clonotypes and their mapped meta‐clonotypes. (C) Distribution of TCR Vβ chain clonotype motifs in donors before G‐CSF mobilisation. (D) Distribution of TCR Vβ chain clonotype motifs in donors after G‐CSF mobilisation. *Source:* Created in BioRender. Li https://BioRender.com/zr70r9i.

Research has indicated that different TCR rearrangement patterns may recognise the same type of antigen or similar antigenic epitopes of the same antigen type [74]. By analysing meta‐clonotypes [53], we could aggregate different TCR rearrangement patterns from a functional perspective to clarify the antigenic similarity recognised by different TCR rearrangement patterns, that is, the similarity of antigen types or epitopes recognised by different TCR rearrangement patterns. Thus, we performed meta‐clonotype analysis of the top 200 (TOP 200), 300 (TOP 300) and 500 (TOP 500) clonotypes. First, we used the OLGA algorithm [55, 56] to remove background TCR clonotypes (BUR) and identify antigen‐enriched TCR clonotypes (AER). Then, we used an ECDF curve to check whether the removal of BUR affects the enrichment of AER (Figure S9). Meta‐clonotype analysis was then conducted on the filtered clonotypes and identified AER (Figure 4A). The results demonstrated a large number of meta‐clonotypes with repeated V(D)J patterns in the TOP 500 group (Table S2), indicating poor aggregation analysis outcomes. The Post group in the TOP 200 failed to yield effective meta‐clonotypes (Table S3); thus, we selected the TOP 300 group for subsequent analyses (Table 1).

**TABLE 1:** Changes in the distribution of TCR Vβ chain clonotypes (Top 300) before and after G‐CSF mobilisation (pre/post).

CDR3 motif	Centroid	Motif‐constraint	Radius	Meta‐Clonotype
Meta‐clonotype pre				
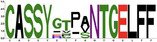	TRBV6‐3*01/TRBJ2‐2*01 CASSYETPANTGELFF	(SY[REG]?[GPSTV][P]?[ASV]NTGEL)	36	TRBV6‐3*01+ CASSYETPANTGELFF + 36+ (SY[REG]?[GPSTV][P]?[ASV]NTGEL)
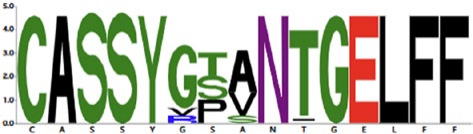	TRBV6‐3*01/TRBJ2‐2*01 CASSYGSANTGELFF	(SY[RGV][PST][ASV]N[T]?GEL)	28	TRBV6‐3*01+CASSYGSANTGELFF + 28+ (SY[RGV][PST][ASV]N[T]?GEL)
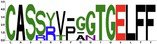	TRBV6‐3*01/TRBJ2‐2*01 CASSRVPGGTGELFF	([RS][RY][TV][P]?[AG]?[NG]TGEL)	36	TRBV6‐3*01+ CASSRVPGGTGELFF + 36+ ([RS][RY][TV][P]?[AG]?[NG]TGEL)
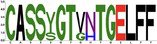	TRBV6‐3*01/TRBJ2‐2*01 CASSSGTGHTGELFF	(S[SY]GT[GV][NH]TGEL)	34	TRBV6‐3*01+ CASSSGTGHTGELFF + 34+ (S[SY]GT[GV][NH]TGEL)
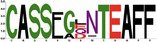	TRBV11‐3*01/TRBJ1‐1*01 CASSFGENTEAFF	(S[LF][AG][DQEL][N]?TEA)	30	TRBV11‐3*01+ CASSFGENTEAFF + 30+ (S[LF][AG][DQEL][N]?TEA)
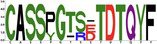	TRBV6‐3*01/TRBJ2‐3*01 CASSPGTSETDTQYF	(S[PY]G[T]?[RS][DE]?TDTQ)	32	TRBV6‐3*01+ CASSPGTSETDTQYF + 32+ (S[PY]G[T]?[RS][DE]?TDTQ)
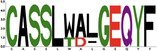	TRBV28*01/TRBJ2‐7*01 CASSLWALGEQYF	(SL[TW][AD][L]?GEQ)	28	TRBV28*01+ CASSLWALGEQYF + 28+ (SL[TW][AD][L]?GEQ)
Meta‐clonotype post				
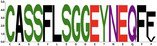	TRBV12‐3*01/TRBJ2‐1*01 CASSFLSGGEYNEQFF	(SFLSGGEYNEQ)	26	TRBV12‐3*01+ CASSFLSGGEYNEQFF+ 26+ (SFLSGGEYNEQ)
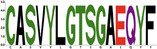	TRBV6‐3*01/TRBJ2‐5*01 CASVYLGTSGAEQYF	(VYLGTSGAEQ)	36	TRBV6‐3*01+ CASVYLGTSGAEQYF+ 36+ (VYLGTSGAEQ)
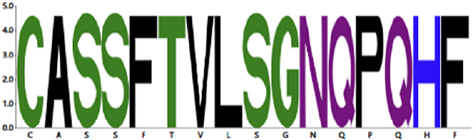	TRBV28*01/TRBJ1‐5*01 CASSFTVLSGNQPQHF	(SFTVLSGNQPQ)	34	TRBV28*01+ CASSFTVLSGNQPQHF + 34+ (SFTVLSGNQPQ)
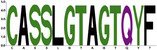	TRBV6‐3*01/TRBJ2‐5*01 CASSLGTAGTQYF	(SLGTAGTQ)	34	TRBV6‐3*01+ CASSLGTAGTQYF + 34+ (SLGTAGTQ)
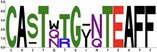	TRBV12‐3*01/TRBJ1‐1*01 CASTQTGINTEAFF	(T[NQW][RT]G[IV]?[NQ]TEA)	36	TRBV12‐3*01+CASTQTGINTEAFF + 36+ (T[NQW][RT]G[IV]?[NQ]TEA)

The meta‐clonotype aggregation results for the TOP 300 group demonstrated that the Pre group aggregated into seven meta‐clonotypes: TRBV28*01‐J2‐7*01 (CASSLWALGEQYF), TRBV6‐3*01‐J2‐2*01 (CASSYGSANTGELFF), TRBV11‐3*01‐J1‐1*01 (CASSFGENTEAFF), TRBV6‐3*01‐J2‐3*01 (CASSPGTSETDTQYF), TRBV6‐3*01‐J2‐2*01 (CASSSGTGHTGELFF), TRBV6‐3*01‐J2‐2*01 (CASSYETPANTGELFF) and TRBV6‐3*01‐J2‐2*01 (CASSRVPGGTGELFF) (Figure 4B,C). The Post group aggregated into five meta‐clonotypes: TRBV12‐3*01‐J2‐1*01 (CASSFLSGGEYNEQFF), TRBV28*01‐J1‐5*01 (CASSFTVLSGNQPQHF), TRBV6‐3*01‐J2‐5*01 (CASSLGTAGTQYF), TRBV6‐3*01‐J2‐5*01 (CASVYLGTSGAEQYF) and TRBV12‐3*01‐J1‐1*01 (CASTQTGINTEAFF) (Figure 4B,D). Analysis of V‐J pairing for the major meta‐clonotype centre clones revealed that the primary V‐J pairings in the Pre group meta‐clonotypes were TRBV6‐3*01 + TRBJ2‐2*01 (5, representing the number of genes) / TRBJ2‐3*01 (1), while the Post group had the primary V‐J pairings of TRBV12‐3*01 + TRBJ2‐1*01 (1)/TRBJ1‐1*01 (1) and TRBV6‐3*01 + TRBJ2‐5*01 (2) (Figure 4B). These results indicate substantial differences in the primary centre AERs between the Pre and Post groups, suggesting significant changes in the central TCR clonotypes of donors after G‐CSF mobilisation. These results indicate that G‐CSF mobilisation may lead to a marked alteration in the types or epitopes of antigens recognised by donor T cells. These findings are consistent with the above analysis of high‐frequency TCR clonotypes, further confirming that G‐CSF treatment can induce significant changes in donor TCR clonotypes.

### 3.4 Potential Molecular Mechanisms of TCR Clonal Changes in Donors Before and After G‐CSF Mobilisation

**FIGURE 5:**
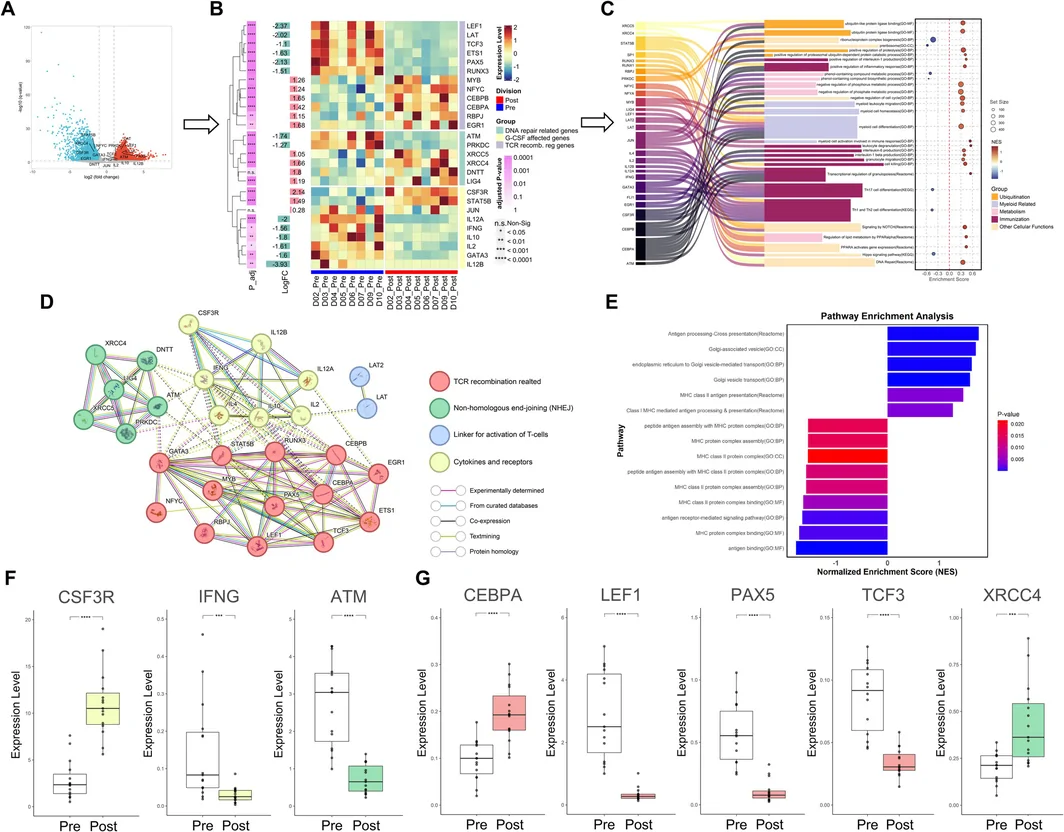
Effects of G‐CSF treatment on TCR rearrangement‐related genes in donors. (A) Volcano plot of differentially expressed genes in donor PBMCs before and after G‐CSF mobilisation. (B) Changes in expression of genes related to TCR rearrangement, DNA repair, and cytokines or receptors that are directly or indirectly affected by G‐CSF. (C) GSEA of the genes involved in TCR rearrangement, DNA repair, and cytokines or receptors impacted by G‐CSF with *p* < 0.05. (D) Protein–Protein Interaction (PPI) analysis of TCR rearrangement, DNA repair, and cytokine or receptor genes directly or indirectly affected by G‐CSF. (E) GSEA pathway analysis of the antigen presentation genes related to G‐CSF treatment in donors. (F) qPCR results of *CSF3R*, *IFNG*, and *ATM* in donor PBMCs before and after G‐CSF treatment. (G) qPCR results of *CEBPA*, *LEF1*, *PAX5*, *TCF3*, and *XRCC4* in donor PBMCs before and after G‐CSF treatment. Statistical methods: (A) Negative binomial generalised linear model likelihood ratio test, GLM‐LRT. *p*‐values were adjusted by the Benjamini–Hochberg (BH) method. (E) Permutation test. *p*‐values were adjusted by the BH method. (F, G) Wilcoxon rank‐sum test. The stars refer to the *p*‐value markers. **** = *p* < 0.0001, *** = *p* < 0.001.

The TCR rearrangement process involves active DNA double‐strand breaks and recombination repair, which is regulated by various genes and factors [20, 21, 23, 25, 29, 30, 32]. In this study, WGCNA results suggested that G‐CSF treatment could alter the nucleic acid repair process in donors (Figure 1D, Figure S1D). To further characterise the potential molecular mechanisms underlying the changes in the donor TCR clonal diversity and high‐frequency clonotype utilisation induced by G‐CSF treatment, we evaluated the expression changes of genes related to DNA repair and TCR rearrangement that have been reported in the literature [20, 21, 22, 32]. The results demonstrated significant downregulation of DNA repair‐related genes, including protein kinase DNA‐activated catalytic subunit (*PRKDC*) and ATM serine/threonine kinase (*ATM*), and TCR rearrangement‐related genes, including lymphoid enhancer binding factor 1 (*LEF1*), *ETS1*, transcription factor 3 (*TCF3*), linker for activation of T cells (*LAT*), *RUNX3* and paired box 5 (*PAX5*), after G‐CSF treatment (Figure 5A,B. Negative binomial generalised linear model likelihood ratio test, GLM‐LRT). Additionally, previous studies have reported that G‐CSF can directly or indirectly regulate the expression of certain cytokines or receptors such as interleukin 10 (*IL10*), *IL12A*, interferon‐γ (*IFNG*) [16, 19, 72, 73, 75], *IL2*, *GATA3,* and *IL12B*. Moreover, RNA‐seq data demonstrated that G‐CSF treatment significantly reduces the expression of these cytokines and receptors. Gene Set Enrichment Analysis (GSEA) of these genes indicated significant changes in cellular biological processes such as ubiquitination (GO:0044389, GO:0031625 and GO:0032436), phosphorylation metabolism (GO:0032436 and GO:0045936), lipid metabolism (R‐HSA‐400206) and DNA repair (R‐HSA‐73894) as well as signalling pathways including the NOTCH signalling pathway (R‐HSA‐157118), the PPARA signalling pathway (R‐HSA‐400206) and the Hippo signalling pathway (KEGG:hsa04390) after G‐CSF treatment (Figure 5C). Protein–protein interaction (PPI) analysis of these genes demonstrates that *CSF3R*, the gene that encodes the G‐CSF receptor, is located in a central position, and it may regulate NHEJ and TCR rearrangement‐related genes through *IFNG/ATM* (Figure 5D). The qPCR results also confirmed that *CSF3R* expression was significantly increased, whereas *IFNG* and *ATM* expression levels were significantly downregulated after G‐CSF treatment. Other genes involved in DNA repair and TCR rearrangement also demonstrated changes to varying degrees (Figure 5F,G). Therefore, it is thought that the *CSF3R‐IFNG‐ATM* signalling axis may be involved in the process of donor TCR clonal diversity and high‐frequency TCR clonotype changes induced by G‐CSF treatment.

Donor CD3^+^ T cells were also subjected to RNA‐seq and TCR‐seq following G‐CSF induction, revealing similar expression patterns for the aforementioned genes (Figure S2D,E). Correlation analysis of key differentially expressed genes with predicted proportions of different T cell subsets revealed varying degrees of correlation between these genes and resting CD4^+^ memory T cells, naive CD4^+^ T cells, and CD8^+^ T cells, while the correlation with activated CD4^+^ memory T cells was not strong (Figure S10). These results suggest that genes involved in regulating TCR rearrangement are primarily associated with CD4^+^ T cells and have partial associations with CD8^+^ T cell subsets.

The process of antigen‐specific T cell recognition of antigens mainly involves antigen presentation and the recognition of antigens by specific TCR clones. Using both CIBERSORT and MCPCOUNTER analysis, we found that the proportion of M0 macrophages (resting state) was increased, while the proportion of dendritic cells (DCs) and B cells was reduced by G‐CSF mobilisation (Figure S1G–I). To further elucidate whether G‐CSF treatment also affects the antigen presentation process, we conducted GSEA on the RNA‐seq results, and as expected, G‐CSF treatment inhibited multiple major histocompatibility complex (MHC)‐related biological processes and cellular components, such as MHC class II protein complex assembly, peptide antigen assembly with the MHC II protein complex, and MHC II protein complex binding (Figure 5E), indicating that G‐CSF treatment may impact the antigen presentation process.

## 4 Discussion

G‐CSF is widely used to mobilise donor HSPCs for allo‐HSCT. Although the proportion of T cells in HSPCs mobilised by G‐CSF is significantly higher than that in bone marrow (BM) grafts, mobilisation of G‐CSF does not increase the incidence or severity of aGVHD in recipients [6, 16]. Several studies have suggested that G‐CSF can alter T cell function, downregulate costimulatory molecules, and mediate the Th1 and Th2 immune response by modulating cytokine expression. Franzke et al. demonstrated that G‐CSF can induce immune tolerance in T cells by upregulating the expression of functionally active G‐CSF receptors on CD4^+^ and CD8^+^ T cells [9, 76]. Analysis of RNA‐seq and WGCNA data in this study preliminarily confirmed that G‐CSF treatment could suppress multiple T cell functions and related signalling pathways in donors. Additionally, we found that G‐CSF mobilisation induces changes in nucleic acid repair, RNA splicing, and ribosomal behaviour in donor PBMCs. Interestingly, WGCNA results also demonstrated that the ubiquitination modification process is affected after G‐CSF mobilisation. Previous studies have shown that ubiquitination is involved in the regulation of T cell function [77, 78]. For example, RING‐box 1 (*Rbx1*) is essential for maintaining Treg effector subsets and loss of *Rbx1* alters the fate of γδ T cells in the mouse thymus [79]. Therefore, we hypothesise that G‐CSF treatment not only suppresses donor T cell function but may also mediate the functional regulation of multiple donor cells, including T cells, at the level of gene expression and regulation.

With regard to TCR types, T cells are primarily composed of two subgroups that are distinguished by the expression of αβ or γδ TCR dimers. αβ^+^ T cells are mainly circulating cells that are divided into two major subgroups: CD4^+^ and CD8^+^ T cells, which primarily recognise MHC‐restricted antigen peptides. γδ^+^ T cells can recognise non‐MHC‐restricted antigen peptides and play a crucial role in pathogen clearance and immune regulation. Several studies have suggested that donor αβ^+^ T cells are the main contributors to recipient aGVHD [80, 81, 82], and results from both rat models and human clinical trials indicate that using anti‐αβ^+^ TCR monoclonal antibodies (mAbs) to deplete T cells from BM grafts can effectively prevent aGVHD. Generation and maintenance of TCR repertoire diversity are key to establishing a stable and effective immune response. Studies have shown that lack of TCR repertoire diversity is associated with a decline in the body's immune response potential. Using qPCR and gene scanning, Dr. Liu's team found that the expression frequency of the donor TRAV and TRBV subfamilies decreased to various degrees after G‐CSF mobilisation [3]. Through TCR immune repertoire sequencing and bioinformatics analysis, we also observed a significant reduction in the clonal diversity of the TCR Vβ chain in donor T cells after G‐CSF mobilisation with a similar trend observed for the TCR V δ chain. The potential impact of reduced TCR clonal diversity in G‐CSF‐mobilised HSPCs on the low incidence of aGVHD requires further investigation, including monitoring clinical outcome following allo‐HSCT and identifying variations in the ability of individual TCR clones to contribute to aGVHD or GVL.

CDR3 is the core region for TCR antigen recognition, and its V(D)J recombination pattern could, to some extent, reflect TCR clonal diversity [54, 83]. The TCR immune repertoire sequencing method could be used in a high‐throughput manner to screen for frequently used TCR V(D)J recombination patterns in tested samples. Studies have shown that the same type of antigen or similar antigenic epitopes may be recognised by multiple different TCR clones, indicating that these different TCR recombination patterns have some functional similarity in antigen recognition. Meta‐clonotype analysis could reflect the similarity of different TCR recombination patterns in recognising antigen types or epitopes. In this study, we used TCRdist3 to perform meta‐clonotype analysis of the top 300 TCR clonotypes selected before and after G‐CSF mobilisation in donors. We found a reduction in meta‐clonotypes and significant changes in the central AER after G‐CSF mobilisation in donors. This reduction indicated that G‐CSF treatment may lead to a decreased potential for antigen/epitope recognition by donor T cells, and it may be a reason for the lower incidence and low grade of GVHD onset after allo‐HSCT. Interestingly, we found that the usage of the separate V, D and J gene segments in the TCR β chain did not change significantly before and after G‐CSF mobilisation, while the V(D)J rearrangement pattern of the CDR3 was significantly changed. The RNA‐seq results also demonstrated that G‐CSF treatment affects the expression of TCR rearrangement‐related genes. Previous research has shown that TCR clonal diversity is influenced by multiple factors, including thymic selection, antigen stimulation, the insertion of non‐template‐encoded nucleotides at V(D)J junctions, selective proliferation of specific T cell clones and others. In this study, all donors were healthy adults, so the likelihood of thymic selection and antigen stimulation was relatively low [84, 85]. Therefore, we hypothesised that the V(D)J rearrangement pattern of CDR3 caused by G‐CSF treatment may be correlated with the insertion or deletion of nucleotides during V, D and J fragment linking, or the expansion of specific TCR clones.

TCR rearrangement is a multi‐gene and mechanism‐regulated process. In this study, we found that G‐CSF mobilisation significantly changed the expression of genes involved in the TCR recombination regulation process. PPI analysis of these genes showed that the *CSF3R‐IFNG‐ATM* signalling axis is in a central position. There have been reports that ATM is associated with DNA double‐strand breaks [86], and this gene may be involved in the V(D)J rearrangement process [87, 88]. Moreover, G‐CSF could reduce the production of IFN‐γ and IL‐4 in T cells during bone marrow transplantation [89]. In addition, G‐CSF receptor‐deficient T cells secreted more IFN‐γ and IL‐17A in a mouse tumour model [90]. These findings preliminarily suggest that the *CSF3R‐IFNG‐ATM* signalling axis may be involved in the reduction in donor TCR clonal diversity caused by G‐CSF mobilisation. However, this is only a speculative hypothesis, and the detailed regulatory mechanism of this signalling axis requires confirmation with further experiments, which is a limitation of this study. Further exploration of the role and detailed mechanism of the *CSF3R‐IFNG‐ATM* signalling pathway in regulating donor TCR clonal diversity and inhibiting T cells after G‐CSF treatment will deepen our understanding of the mechanism of G‐CSF‐mediated low aGVHD after allo‐HSCT. In addition, the differentially expressed genes were positively correlated with the proportion of different T cell subsets before and after G‐CSF mobilisation and were mainly associated with multiple CD4^+^ T cell subsets. We also found that multiple biological activities involved in antigen presentation were significantly inhibited, including multiple MHC II molecule‐related biological behaviours. Moreover, immune cell infiltration analysis indicated a decrease in B cells after G‐CSF mobilisation in donors. The above findings suggest that a potential mechanism by which G‐CSF mediates the low incidence of aGVHD after transplantation is by interfering with TCR recombination and antigen presentation, thereby weakening or inhibiting the recognition of recipient antigens by donor T cells, and G‐CSF may have a greater effect on the immune function of CD4^+^ T cells. In contrast, alterations in CD8^+^ T cells were minimal in this study, which may suggest that G‐CSF treatment has less impact on CD8^+^ T cells while still maintaining GVL ability for allo‐HSCT.

## 5 Conclusions

To the best of our knowledge, we for the first time characterised the alterations in donor T cells after G‐CSF mobilisation. Significantly, G‐CSF mobilisation may affect donor TCR recombination‐related genes, T cell function‐related processes and antigen recognition‐related processes, thereby mediating a reduction in donor TCR recombination and weakening T cell function, revealing a primary explanation for the low incidence of GVHD mediated by G‐CSF after allo‐HSCT.

## Author Contributions

Y. L., Y. Z., J. C., and L. Z. conceptualised and designed the study. Z. C., X. Z., Z. L., and M. P. designed and conducted the majority of the cell experiments. X. L. and Y. Z. contributed to the transcriptomic and immune repertoire analysis. J. C. and J. L. mainly performed the G‐CSF mobilisation and evaluation of donors. J. Z., X. L., Z. C. B. Y., and Y. L. collected most of the blood samples, performed flow cytometry, and analysed the results. X. L., Z. C., and Y. Z. wrote the original manuscript. Y. L., S. C., J. C., and X. Z. supervised and edited the original manuscript. Co‐first authorship and order were determined by the contribution to the results presented in the manuscript and contribution to writing the initial manuscript draft.

## Funding

This study was supported by grants from the National Natural Science Foundation of China (82293632, 82293630, 92474101, and 82400259), the Fundamental Research Funds for the Central Universities (21623121 and 21625219), the Science and Technology Project in Guangzhou (202102070001), and the National College Students' Innovation and Entrepreneurship Training Program (202510559118).

## Conflicts of Interest

The authors declare no conflicts of interest.

## Supporting information


**Table S1:** Oligonucleotide sequences for qPCR.
**Table S2:** Changes in the distribution of TCR Vβ chain clonotypes (Top 500) before and after G‐CSF mobilisation (pre/post)
**Table S3:** Distribution of TCR Vβ chain clonotypes (Top 200) before G‐CSF mobilisation

**Figure S1:** WGCNA module‐trait and gene correlation before and after screening. (A) Correlation between WGCNA modules and traits. (B) Topological Overlap Matrix (TOM) of all genes analysed, where light colours represent low overlap and progressively darker shades of red indicate higher overlap. (C) Average gene significance of each WGCNA module. (D‐F) Scatter plots of trait‐related modules and gene significance for other significant modules. (G) Box plot of changes in the proportions of various immune cells before and after donor mobilisation predicted by CIBERSORT. (H) Box plot of changes in the proportions of various immune cells before and after donor mobilisation predicted by MCPCOUNTER. (I) Box plot of changes in the proportions of various immune cells before and after donor mobilisation predicted by QuantiSEQ. Statistical methods: (A‐F) Pearson correlation. (G‐I) Wilcoxon rank‐sum test. The stars refer to the *p*‐value markers. *** = *p* < 0.001, ** = *p* < 0.01, * = *p* < 0.05, ns = *p* > 0.05.

**Figure S2:** G‐CSF treatment affects the distribution of T cell subsets and TCR clonal diversity in human CD3^+^ T cells in vitro. (A) Gating strategies for the cell populations. (B) Flow cytometry results of human CD3^+^ T cells treated with G‐CSF in vitro. (C) Proportions of activated, resting CD4^+^ T cells and CD8^+^ T cells from human CD3^+^ T cells treated with G‐CSF (*n* = 4). (D) RNA‐seq results of G‐CSF‐related genes in human CD3^+^ T cells treated with G‐CSF in vitro (*n* = 3). (E) The TCR‐seq results of human CD3^+^ T cells treated with G‐CSF in vitro (*n* = 3). Statistical methods: (C) Wilcoxon rank‐sum test. (D‐E) Paired sample t‐test. The stars refer to the *p*‐value markers. *** = *p* < 0.001, ** = *p* < 0.01, * = *p* < 0.05, ns = *p* > 0.05.

**Figure S3:** Changes in high‐frequency usage of TCR Vβ chain V, J, and D gene segments in donors before and after G‐CSF treatment. (A) V region gene changes. (B) J region gene changes. (C) D region gene changes.

**Figure S4:** Changes in high‐frequency usage of the TCR Vδ chain V, J, and D gene segments in donors before and after G‐CSF treatment. (A) V region gene changes. (B) J region gene changes. (C) D region gene changes.

**Figure S5:** Mathematical analysis showing the decline in TCR diversity in donors before and after G‐CSF mobilisation. (A) D50 statistical analysis of donor TCR Vβ chain clones before and after G‐CSF mobilisation. (B) Chao1 statistical analysis of donor TCR Vβ chain clones before and after G‐CSF mobilisation. (C) D50 statistical analysis of donor TCR Vδ chain clones before and after G‐CSF mobilisation. (D) Chao1 statistical analysis of donor TCR Vδ chain clones before and after G‐CSF mobilisation. (E) Clone index of donor TCR Vβ chain clones before and after G‐CSF mobilisation. (F) Clone index of donor TCR Vδ chain clones before and after G‐CSF mobilisation. Statistical methods: Wilcoxon rank‐sum test.

**Figure S6:** G‐CSF treatment induces changes in high‐frequency donor TCR clones. (A) Expression of the top 20 high‐frequency TCR Vβ clones in different donors before and after G‐CSF mobilisation. (B) Expression of the top 20 high‐frequency TCR Vδ clones in different donors before and after G‐CSF mobilisation.

**Figure S7:** Differences in the top 20 high‐frequency TCR Vβ clones in different donors before and after G‐CSF mobilisation.

**Figure S8:** Differences in the top 20 high‐frequency TCR Vδ clones in different donors before and after G‐CSF mobilisation.

**Figure S9:** ECDF curves for antigen‐related and background TCR clones before and after G‐CSF mobilisation. (A) ECDF plot for the top 200 clonotypes. (B) ECDF plot for the top 300 clonotypes. (C) ECDF plot for the top 500 clonotypes.

**Figure S10:** Correlation analysis of the key differentially expressed genes and proportions of the various cell populations predicted by CIBERSORT before and after G‐CSF mobilisation. (A) Correlation between the proportion of resting CD4^+^ memory T cells, activated CD4^+^ memory T cells, naive CD4^+^ T cells, and CD8^+^ T cells and the expression of *CSF3R*, *IFNG* and *ATM*. (B) Correlation between the proportion of resting CD4^+^ memory T cells, activated CD4^+^ memory T cells, naive CD4^+^ T cells, and CD8^+^ T cells and the expression of key TCR rearrangement‐related genes. Statistical methods: Pearson correlation.

## Data Availability

The data that support the findings of this study are available upon request from the corresponding author. The data are not publicly available due to privacy or ethical restrictions.
