# Supplementary material for: Impact of G‐CSF on Donor TCR Clonal Diversity and T Cell Function During Donor HSC Mobilisation
Source: Cell Prolif. 2026 Apr 16;59(9):e70213. doi: 10.1111/cpr.70213 (PMC13325648; doi:10.1111/cpr.70213)
Supplement: Supplementary file 1 [file CPR-59-e70213-s001.pdf]

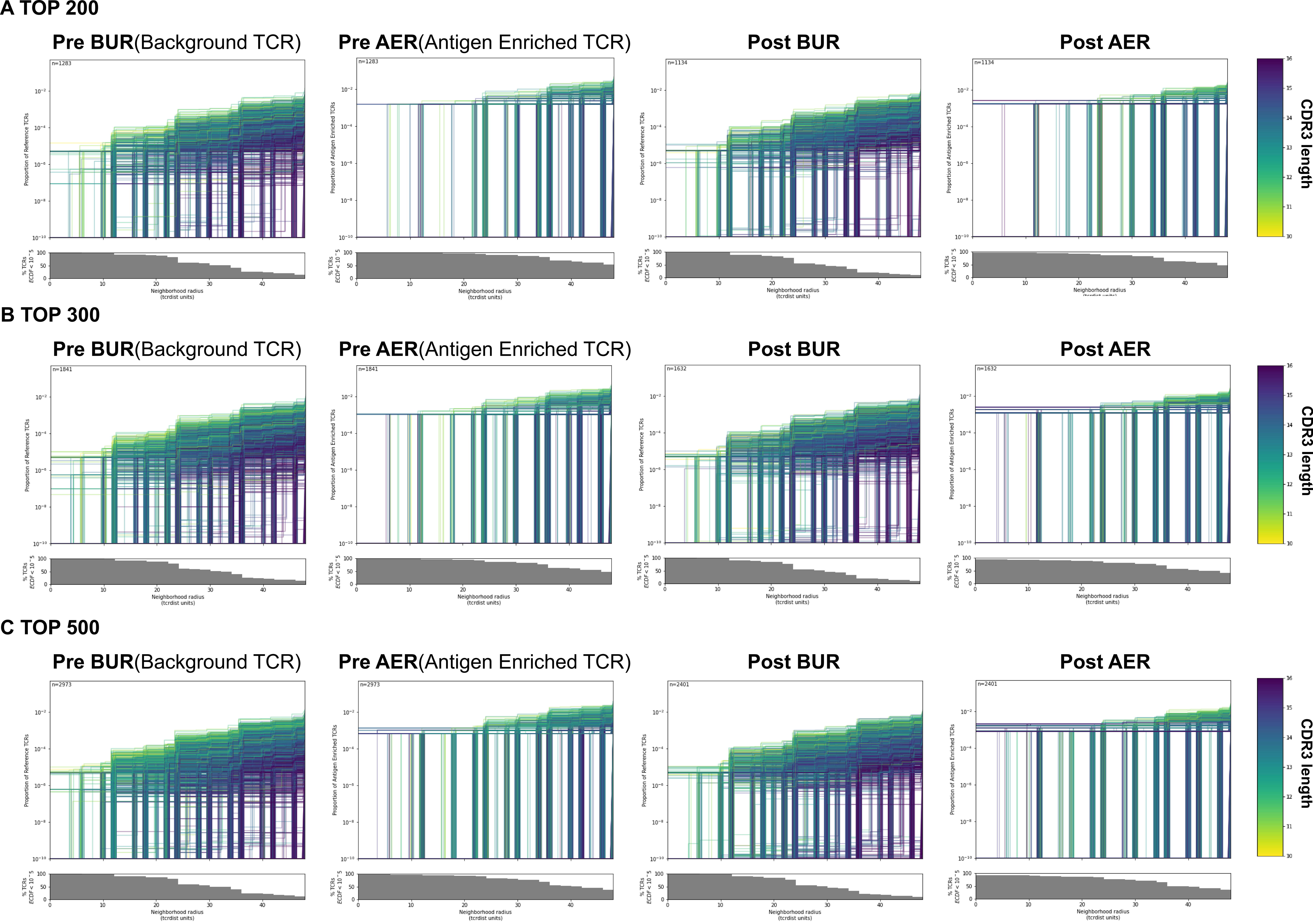
A TOP 200
Pre BUR(Background TCR)
Pre AER(Antigen Enriched TCR)
Post BUR
Post AER
n=1283
n=1283
n=1134
n=1134
B TOP 300
Pre BUR(Background TCR)
Pre AER(Antigen Enriched TCR)
Post BUR
Post AER
n=1841
n=1841
n=1632
n=1632
C TOP 500
Pre BUR(Background TCR)
Pre AER(Antigen Enriched TCR)
Post BUR
Post AER
n=2973
n=2973
n=2401
n=2401
Proportion of Reference TCRs
Proportion of Antigen Enriched TCRs
Neighborhood radius
(tcrdist units)
CDR3 length
