# Supplementary material for: Impact of G‐CSF on Donor TCR Clonal Diversity and T Cell Function During Donor HSC Mobilisation
Source: Cell Prolif. 2026 Apr 16;59(9):e70213. doi: 10.1111/cpr.70213 (PMC13325648; doi:10.1111/cpr.70213)
Supplement: Supplementary file 2 [file CPR-59-e70213-s002.pdf]

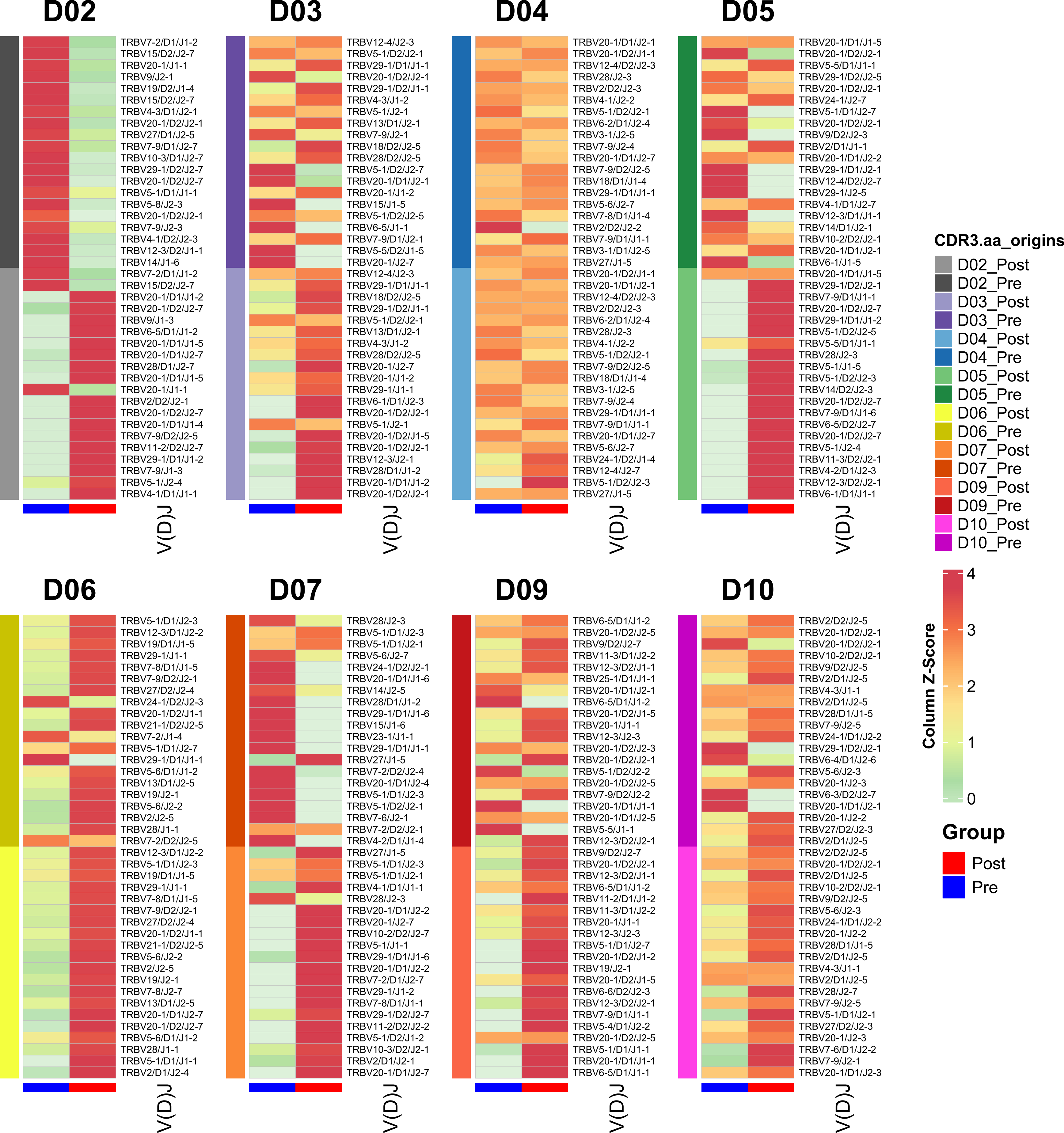
D02
TRBV7-2/D1/J1-2
TRBV15/D2/J2-7
TRBV20-1/J1-1
TRBV9/J2-1
TRBV19/D2/J1-4
TRBV15/D2/J2-7
TRBV4-3/D1/J2-1
TRBV20-1/D2/J2-1
TRBV27/D1/J2-5
TRBV7-9/D1/J2-7
TRBV10-3/D1/J2-7
TRBV29-1/D2/J2-7
TRBV20-1/D2/J2-7
TRBV5-1/D1/J1-1
TRBV5-8/J2-3
TRBV20-1/D2/J2-1
TRBV7-9/J2-3
TRBV4-1/D2/J2-3
TRBV12-3/D2/J1-1
TRBV14/J1-6
TRBV7-2/D1/J1-2
TRBV15/D2/J2-7
TRBV20-1/D1/J1-2
TRBV20-1/D2/J2-7
TRBV9/J1-3
TRBV6-5/D1/J1-2
TRBV20-1/D1/J1-5
TRBV20-1/D1/J2-7
TRBV28/D1/J2-7
TRBV20-1/D1/J1-5
TRBV20-1/J1-1
TRBV2/D2/J2-1
TRBV20-1/D2/J2-7
TRBV20-1/D1/J1-4
TRBV7-9/D2/J2-5
TRBV11-2/D2/J2-7
TRBV29-1/D1/J1-2
TRBV7-9/J1-3
TRBV5-1/J2-4
TRBV4-1/D1/J1-1
V(D)J
D03
TRBV12-4/J2-3
TRBV5-1/D2/J2-1
TRBV29-1/D1/J1-1
TRBV20-1/D2/J2-1
TRBV29-1/D2/J1-1
TRBV4-3/J1-2
TRBV5-1/J2-1
TRBV13/D1/J2-1
TRBV7-9/J2-1
TRBV18/D2/J2-5
TRBV28/D2/J2-5
TRBV5-1/D2/J2-7
TRBV20-1/D1/J2-1
TRBV20-1/J1-2
TRBV15/J1-5
TRBV5-1/D2/J2-5
TRBV6-5/J1-1
TRBV7-9/D1/J2-1
TRBV5-5/D2/J1-5
TRBV20-1/J2-7
TRBV12-4/J2-3
TRBV29-1/D1/J1-1
TRBV18/D2/J2-5
TRBV29-1/D2/J1-1
TRBV5-1/D2/J2-1
TRBV13/D1/J2-1
TRBV4-3/J1-2
TRBV28/D2/J2-5
TRBV20-1/J2-7
TRBV20-1/J1-2
TRBV29-1/J1-1
TRBV6-1/D1/J2-3
TRBV20-1/D2/J2-1
TRBV5-1/J2-1
TRBV20-1/D2/J1-5
TRBV20-1/D2/J2-1
TRBV12-3/J2-1
TRBV28/D1/J1-2
TRBV20-1/D1/J1-2
TRBV20-1/D2/J2-1
V(D)J
D04
TRBV20-1/D1/J2-1
TRBV20-1/D2/J1-1
TRBV12-4/D2/J2-3
TRBV28/J2-3
TRBV2/D2/J2-3
TRBV4-1/J2-2
TRBV5-1/D2/J2-1
TRBV6-2/D1/J2-4
TRBV3-1/J2-5
TRBV7-9/J2-4
TRBV20-1/D1/J2-7
TRBV7-9/D2/J2-5
TRBV18/D1/J1-4
TRBV29-1/D1/J1-1
TRBV5-6/J2-7
TRBV7-8/D1/J1-4
TRBV2/D2/J2-2
TRBV7-9/D1/J1-1
TRBV3-1/D1/J2-5
TRBV27/J1-5
TRBV20-1/D2/J1-1
TRBV20-1/D1/J2-1
TRBV12-4/D2/J2-3
TRBV2/D2/J2-3
TRBV6-2/D1/J2-4
TRBV28/J2-3
TRBV4-1/J2-2
TRBV5-1/D2/J2-1
TRBV7-9/D2/J2-5
TRBV18/D1/J1-4
TRBV3-1/J2-5
TRBV7-9/J2-4
TRBV29-1/D1/J1-1
TRBV7-9/D1/J1-1
TRBV20-1/D1/J2-7
TRBV5-6/J2-7
TRBV24-1/D2/J1-4
TRBV12-4/J2-7
TRBV5-1/D2/J2-3
TRBV27/J1-5
V(D)J
D05
TRBV20-1/D1/J1-5
TRBV20-1/D2/J2-1
TRBV5-5/D1/J1-1
TRBV29-1/D2/J2-5
TRBV20-1/D2/J2-1
TRBV24-1/J2-7
TRBV5-1/D1/J2-7
TRBV20-1/D2/J2-1
TRBV9/D2/J2-3
TRBV2/D1/J1-1
TRBV20-1/D1/J2-2
TRBV29-1/D1/J2-1
TRBV12-4/D2/J2-7
TRBV29-1/J2-5
TRBV4-1/D1/J2-7
TRBV12-3/D1/J1-1
TRBV14/D1/J2-1
TRBV10-2/D2/J2-1
TRBV20-1/D1/J2-1
TRBV6-1/J1-5
TRBV20-1/D1/J1-5
TRBV29-1/D2/J2-1
TRBV7-9/D1/J1-1
TRBV20-1/D2/J2-7
TRBV29-1/D1/J1-2
TRBV5-1/D2/J2-5
TRBV5-5/D1/J1-1
TRBV28/J2-3
TRBV5-1/J1-5
TRBV5-1/D2/J2-3
TRBV14/D2/J2-3
TRBV20-1/D2/J2-7
TRBV7-9/D1/J1-6
TRBV6-5/D2/J2-7
TRBV20-1/D2/J2-7
TRBV5-1/J2-4
TRBV11-3/D2/J2-1
TRBV4-2/D1/J2-3
TRBV12-3/D2/J2-1
TRBV6-1/D1/J1-1
V(D)J
D06
TRBV5-1/D1/J2-3
TRBV12-3/D1/J2-2
TRBV19/D1/J1-5
TRBV29-1/J1-1
TRBV7-8/D1/J1-5
TRBV7-9/D2/J2-1
TRBV27/D2/J2-4
TRBV24-1/D2/J2-3
TRBV20-1/D2/J1-1
TRBV21-1/D2/J2-5
TRBV7-2/J1-4
TRBV5-1/D1/J2-7
TRBV29-1/D1/J1-1
TRBV5-6/D1/J1-2
TRBV13/D1/J2-5
TRBV19/J2-1
TRBV5-6/J2-2
TRBV2/J2-5
TRBV28/J1-1
TRBV7-2/D2/J2-5
TRBV12-3/D1/J2-2
TRBV5-1/D1/J2-3
TRBV19/D1/J1-5
TRBV29-1/J1-1
TRBV7-8/D1/J1-5
TRBV7-9/D2/J2-1
TRBV27/D2/J2-4
TRBV20-1/D2/J1-1
TRBV21-1/D2/J2-5
TRBV5-6/J2-2
TRBV2/J2-5
TRBV19/J2-1
TRBV7-8/J2-7
TRBV13/D1/J2-5
TRBV20-1/D1/J2-7
TRBV20-1/D2/J2-7
TRBV5-6/D1/J1-2
TRBV28/J1-1
TRBV5-1/D1/J1-1
TRBV2/D1/J2-4
V(D)J
D07
TRBV28/J2-3
TRBV5-1/D1/J2-3
TRBV5-1/D1/J2-1
TRBV5-6/J2-7
TRBV24-1/D2/J2-1
TRBV20-1/D1/J1-6
TRBV14/J2-5
TRBV28/D1/J1-2
TRBV29-1/D1/J1-6
TRBV15/J1-6
TRBV23-1/J1-1
TRBV29-1/D1/J1-1
TRBV27/J1-5
TRBV7-2/D2/J2-4
TRBV20-1/D1/J2-4
TRBV5-1/D1/J2-3
TRBV5-1/D2/J2-1
TRBV7-6/J2-1
TRBV7-2/D2/J2-1
TRBV4-2/D1/J1-4
TRBV27/J1-5
TRBV5-1/D1/J2-3
TRBV5-1/D1/J2-1
TRBV4-1/D1/J1-1
TRBV28/J2-3
TRBV20-1/D1/J2-2
TRBV20-1/J2-7
TRBV10-2/D2/J2-7
TRBV5-1/J1-1
TRBV29-1/D1/J1-6
TRBV20-1/D1/J2-2
TRBV7-2/D1/J2-7
TRBV29-1/J1-2
TRBV7-8/D1/J1-1
TRBV29-1/D2/J2-7
TRBV11-2/D2/J2-2
TRBV5-1/D2/J1-2
TRBV10-3/D2/J2-1
TRBV2/D1/J2-1
TRBV20-1/D1/J2-7
V(D)J
D09
TRBV6-5/D1/J1-2
TRBV20-1/D2/J2-5
TRBV9/D2/J2-7
TRBV11-3/D1/J2-2
TRBV12-3/D2/J1-1
TRBV25-1/D1/J1-1
TRBV20-1/D1/J2-1
TRBV6-5/D1/J1-2
TRBV20-1/D2/J1-5
TRBV20-1/J1-1
TRBV12-3/J2-3
TRBV20-1/D2/J2-3
TRBV20-1/D2/J2-1
TRBV5-1/D2/J2-2
TRBV20-1/D2/J2-5
TRBV7-9/D2/J2-2
TRBV20-1/D1/J1-1
TRBV20-1/D1/J2-5
TRBV5-5/J1-1
TRBV12-3/D2/J2-1
TRBV9/D2/J2-7
TRBV20-1/D2/J2-1
TRBV12-3/D2/J1-1
TRBV6-5/D1/J1-2
TRBV11-2/D1/J1-2
TRBV11-3/D1/J2-2
TRBV20-1/J1-1
TRBV12-3/J2-3
TRBV5-1/D1/J2-7
TRBV20-1/D2/J1-2
TRBV19/J2-1
TRBV20-1/D2/J1-5
TRBV6-6/D2/J2-3
TRBV12-3/D2/J2-1
TRBV7-9/D1/J1-1
TRBV5-4/D1/J2-2
TRBV20-1/D2/J2-5
TRBV5-1/D1/J1-1
TRBV20-1/D1/J1-1
TRBV6-5/D1/J1-1
V(D)J
D10
TRBV2/D2/J2-5
TRBV20-1/D2/J2-1
TRBV20-1/D2/J2-1
TRBV10-2/D2/J2-1
TRBV9/D2/J2-5
TRBV2/D1/J2-5
TRBV4-3/J1-1
TRBV2/D1/J2-5
TRBV28/D1/J1-5
TRBV7-9/J2-5
TRBV24-1/D1/J2-2
TRBV29-1/D2/J2-1
TRBV6-4/D1/J2-6
TRBV5-6/J2-3
TRBV20-1/J2-3
TRBV6-3/D2/J2-7
TRBV20-1/D1/J2-1
TRBV20-1/J2-2
TRBV27/D2/J2-3
TRBV2/D1/J2-5
TRBV2/D2/J2-5
TRBV20-1/D2/J2-1
TRBV2/D1/J2-5
TRBV10-2/D2/J2-1
TRBV9/D2/J2-5
TRBV5-6/J2-3
TRBV24-1/D1/J2-2
TRBV20-1/J2-2
TRBV28/D1/J1-5
TRBV2/D1/J2-5
TRBV4-3/J1-1
TRBV2/D1/J2-5
TRBV28/J2-7
TRBV7-9/J2-5
TRBV5-1/D1/J2-1
TRBV27/D2/J2-3
TRBV20-1/J2-3
TRBV7-6/D1/J2-2
TRBV7-9/J2-1
TRBV20-1/D1/J2-3
V(D)J
CDR3.aa_origins
D02_Post
D02_Pre
D03_Post
D03_Pre
D04_Post
D04_Pre
D05_Post
D05_Pre
D06_Post
D06_Pre
D07_Post
D07_Pre
D09_Post
D09_Pre
D10_Post
D10_Pre
Column Z-Score
4
3
2
1
0
Group
Post
Pre
