# Supplementary material for: Impact of G‐CSF on Donor TCR Clonal Diversity and T Cell Function During Donor HSC Mobilisation
Source: Cell Prolif. 2026 Apr 16;59(9):e70213. doi: 10.1111/cpr.70213 (PMC13325648; doi:10.1111/cpr.70213)
Supplement: Supplementary file 3 [file CPR-59-e70213-s003.pdf]

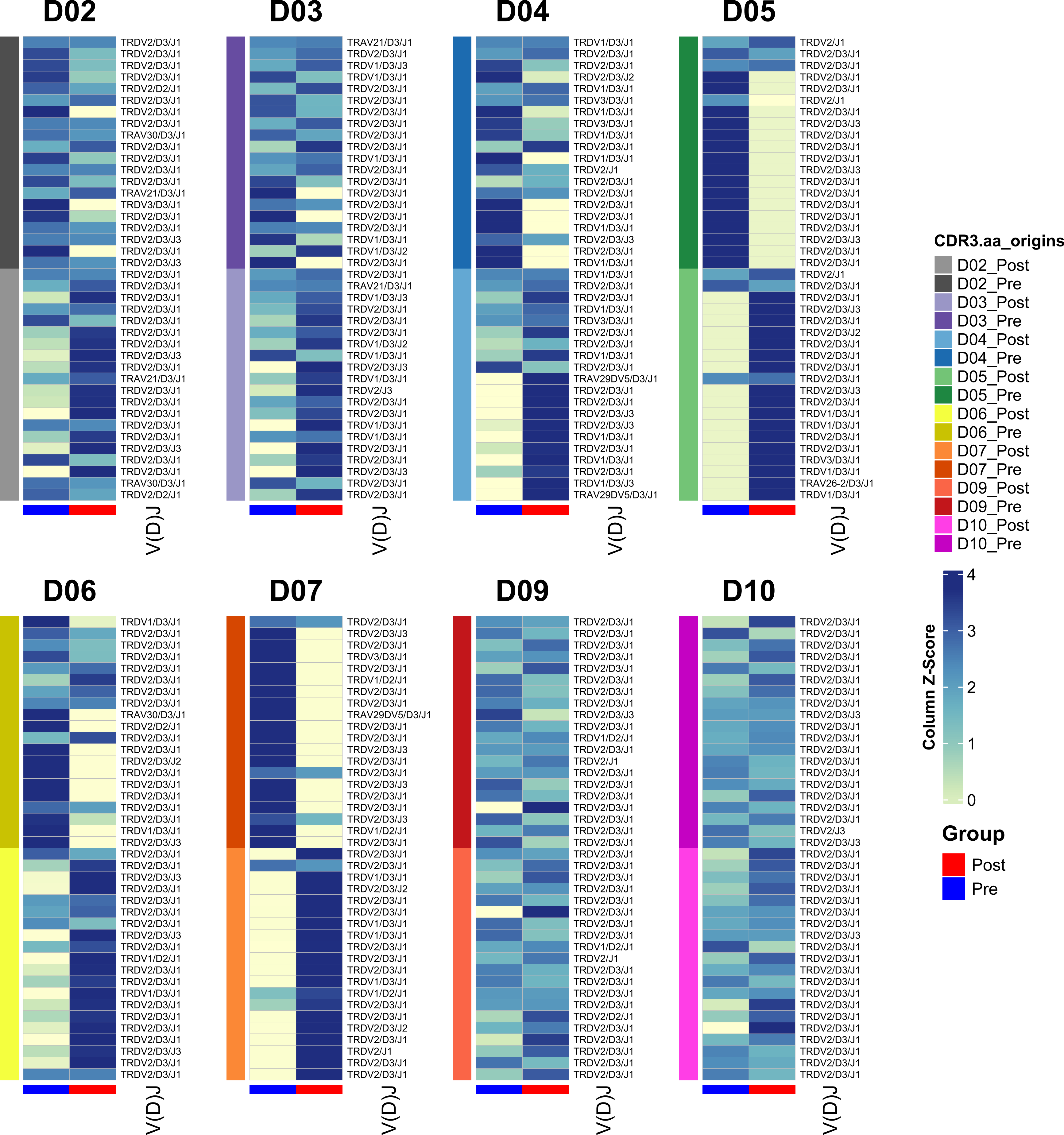

D02
TRDV2/D3/J1
TRDV2/D3/J1
TRDV2/D3/J1
TRDV2/D3/J1
TRDV2/D2/J1
TRDV2/D3/J1
TRDV2/D3/J1
TRDV2/D3/J1
TRAV30/D3/J1
TRDV2/D3/J1
TRDV2/D3/J1
TRDV2/D3/J1
TRDV2/D3/J1
TRAV21/D3/J1
TRDV3/D3/J1
TRDV2/D3/J1
TRDV2/D3/J1
TRDV2/D3/J3
TRDV2/D3/J1
TRDV2/D3/J3
TRDV2/D3/J1
TRDV2/D3/J1
TRDV2/D3/J1
TRDV2/D3/J1
TRDV2/D3/J1
TRDV2/D3/J1
TRDV2/D3/J1
TRDV2/D3/J3
TRDV2/D3/J1
TRAV21/D3/J1
TRDV2/D3/J1
TRDV2/D3/J1
TRDV2/D3/J1
TRDV2/D3/J1
TRDV2/D3/J1
TRDV2/D3/J3
TRDV2/D3/J1
TRDV2/D3/J1
TRAV30/D3/J1
TRDV2/D2/J1
V(D)J
D03
TRAV21/D3/J1
TRDV2/D3/J1
TRDV1/D3/J3
TRDV1/D3/J1
TRDV2/D3/J1
TRDV2/D3/J1
TRDV2/D3/J1
TRDV2/D3/J1
TRDV2/D3/J1
TRDV2/D3/J1
TRDV1/D3/J1
TRDV2/D3/J1
TRDV2/D3/J1
TRDV2/D3/J1
TRDV2/D3/J1
TRDV2/D3/J1
TRDV2/D3/J1
TRDV1/D3/J1
TRDV1/D3/J2
TRDV2/D3/J1
TRDV2/D3/J1
TRAV21/D3/J1
TRDV1/D3/J3
TRDV2/D3/J1
TRDV2/D3/J1
TRDV2/D3/J1
TRDV1/D3/J2
TRDV1/D3/J1
TRDV2/D3/J3
TRDV1/D3/J1
TRDV2/J3
TRDV2/D3/J1
TRDV2/D3/J1
TRDV1/D3/J1
TRDV1/D3/J1
TRDV2/D3/J1
TRDV2/D3/J1
TRDV2/D3/J3
TRDV2/D3/J1
TRDV2/D3/J1
V(D)J
D04
TRDV1/D3/J1
TRDV2/D3/J1
TRDV2/D3/J1
TRDV2/D3/J2
TRDV1/D3/J1
TRDV3/D3/J1
TRDV1/D3/J1
TRDV3/D3/J1
TRDV1/D3/J1
TRDV2/D3/J1
TRDV1/D3/J1
TRDV2/J1
TRDV2/D3/J1
TRDV2/D3/J1
TRDV2/D3/J1
TRDV2/D3/J1
TRDV1/D3/J1
TRDV2/D3/J3
TRDV2/D3/J1
TRDV1/D3/J1
TRDV1/D3/J1
TRDV2/D3/J1
TRDV2/D3/J1
TRDV1/D3/J1
TRDV3/D3/J1
TRDV2/D3/J1
TRDV2/D3/J1
TRDV1/D3/J1
TRDV2/D3/J1
TRAV29DV5/D3/J1
TRDV2/D3/J1
TRDV2/D3/J1
TRDV2/D3/J3
TRDV2/D3/J3
TRDV1/D3/J1
TRDV2/D3/J1
TRDV1/D3/J1
TRDV2/D3/J1
TRDV1/D3/J3
TRAV29DV5/D3/J1
V(D)J
D05
TRDV2/J1
TRDV2/D3/J1
TRDV2/D3/J1
TRDV2/D3/J1
TRDV2/D3/J1
TRDV2/J1
TRDV2/D3/J1
TRDV2/D3/J3
TRDV2/D3/J1
TRDV2/D3/J1
TRDV2/D3/J1
TRDV2/D3/J3
TRDV2/D3/J1
TRDV2/D3/J1
TRDV2/D3/J1
TRDV2/D3/J1
TRDV2/D3/J1
TRDV2/D3/J3
TRDV2/D3/J1
TRDV2/D3/J1
TRDV2/J1
TRDV2/D3/J1
TRDV2/D3/J1
TRDV2/D3/J3
TRDV2/D3/J1
TRDV2/D3/J2
TRDV2/D3/J1
TRDV2/D3/J1
TRDV2/D3/J1
TRDV2/D3/J1
TRDV2/D3/J3
TRDV2/D3/J1
TRDV1/D3/J1
TRDV1/D3/J1
TRDV2/D3/J1
TRDV2/D3/J1
TRDV3/D3/J1
TRDV1/D3/J1
TRAV26-2/D3/J1
TRDV1/D3/J1
V(D)J
CDR3.aa_origins
D02_Post
D02_Pre
D03_Post
D03_Pre
D04_Post
D04_Pre
D05_Post
D05_Pre
D06_Post
D06_Pre
D07_Post
D07_Pre
D09_Post
D09_Pre
D10_Post
D10_Pre
Column Z-Score
4
3
2
1
0
Group
Post
Pre
D06
TRDV1/D3/J1
TRDV2/D3/J1
TRDV2/D3/J1
TRDV2/D3/J1
TRDV2/D3/J1
TRDV2/D3/J1
TRDV2/D3/J1
TRDV2/D3/J1
TRAV30/D3/J1
TRDV2/D2/J1
TRDV2/D3/J1
TRDV2/D3/J1
TRDV2/D3/J2
TRDV2/D3/J1
TRDV2/D3/J1
TRDV2/D3/J1
TRDV2/D3/J1
TRDV2/D3/J1
TRDV1/D3/J1
TRDV2/D3/J3
TRDV2/D3/J1
TRDV2/D3/J1
TRDV2/D3/J3
TRDV2/D3/J1
TRDV2/D3/J1
TRDV2/D3/J1
TRDV2/D3/J1
TRDV2/D3/J3
TRDV2/D3/J1
TRDV1/D2/J1
TRDV2/D3/J1
TRDV2/D3/J1
TRDV1/D3/J1
TRDV2/D3/J1
TRDV2/D3/J1
TRDV2/D3/J1
TRDV2/D3/J1
TRDV2/D3/J3
TRDV2/D3/J1
TRDV2/D3/J1
V(D)J
D07
TRDV2/D3/J1
TRDV2/D3/J3
TRDV2/D3/J1
TRDV3/D3/J1
TRDV2/D3/J1
TRDV1/D2/J1
TRDV2/D3/J1
TRDV2/D3/J1
TRAV29DV5/D3/J1
TRDV2/D3/J1
TRDV2/D3/J1
TRDV2/D3/J3
TRDV2/D3/J1
TRDV2/D3/J1
TRDV2/D3/J3
TRDV2/D3/J1
TRDV2/D3/J1
TRDV2/D3/J3
TRDV1/D2/J1
TRDV2/D3/J1
TRDV2/D3/J1
TRDV2/D3/J1
TRDV1/D3/J1
TRDV2/D3/J2
TRDV2/D3/J1
TRDV2/D3/J1
TRDV1/D3/J1
TRDV1/D3/J1
TRDV2/D3/J1
TRDV2/D3/J1
TRDV2/D3/J1
TRDV1/D3/J1
TRDV1/D2/J1
TRDV2/D3/J1
TRDV2/D3/J1
TRDV2/D3/J2
TRDV2/D3/J1
TRDV2/J1
TRDV2/D3/J1
TRDV2/D3/J1
V(D)J
D09
TRDV2/D3/J1
TRDV2/D3/J1
TRDV2/D3/J1
TRDV2/D3/J1
TRDV2/D3/J1
TRDV2/D3/J1
TRDV2/D3/J1
TRDV2/D3/J1
TRDV2/D3/J3
TRDV2/D3/J1
TRDV1/D2/J1
TRDV2/D3/J1
TRDV2/J1
TRDV2/D3/J1
TRDV2/D3/J1
TRDV2/D3/J1
TRDV2/D3/J1
TRDV2/D3/J1
TRDV2/D3/J1
TRDV2/D3/J1
TRDV2/D3/J1
TRDV2/D3/J1
TRDV2/D3/J1
TRDV2/D3/J1
TRDV2/D3/J1
TRDV2/D3/J1
TRDV2/D3/J1
TRDV2/D3/J1
TRDV1/D2/J1
TRDV2/J1
TRDV2/D3/J1
TRDV2/D3/J1
TRDV2/D3/J1
TRDV2/D3/J1
TRDV2/D2/J1
TRDV2/D3/J1
TRDV2/D3/J1
TRDV2/D3/J1
TRDV2/D3/J1
TRDV2/D3/J1
V(D)J
D10
TRDV2/D3/J1
TRDV2/D3/J1
TRDV2/D3/J1
TRDV2/D3/J1
TRDV2/D3/J1
TRDV2/D3/J1
TRDV2/D3/J1
TRDV2/D3/J1
TRDV2/D3/J3
TRDV2/D3/J1
TRDV2/D3/J1
TRDV2/D3/J1
TRDV2/D3/J1
TRDV2/D3/J1
TRDV2/D3/J1
TRDV2/D3/J1
TRDV2/D3/J1
TRDV2/D3/J1
TRDV2/J3
TRDV2/D3/J3
TRDV2/D3/J1
TRDV2/D3/J1
TRDV2/D3/J1
TRDV2/D3/J1
TRDV2/D3/J1
TRDV2/D3/J1
TRDV2/D3/J1
TRDV2/D3/J3
TRDV2/D3/J1
TRDV2/D3/J1
TRDV2/D3/J1
TRDV2/D3/J1
TRDV2/D3/J1
TRDV2/D3/J1
TRDV2/D3/J1
TRDV2/D3/J1
TRDV2/D3/J1
TRDV2/D3/J1
TRDV2/D3/J1
TRDV2/D3/J1
V(D)J
