# Supplementary material for: Impact of G‐CSF on Donor TCR Clonal Diversity and T Cell Function During Donor HSC Mobilisation
Source: Cell Prolif. 2026 Apr 16;59(9):e70213. doi: 10.1111/cpr.70213 (PMC13325648; doi:10.1111/cpr.70213)
Supplement: Supplementary file 4 [file CPR-59-e70213-s004.pdf]

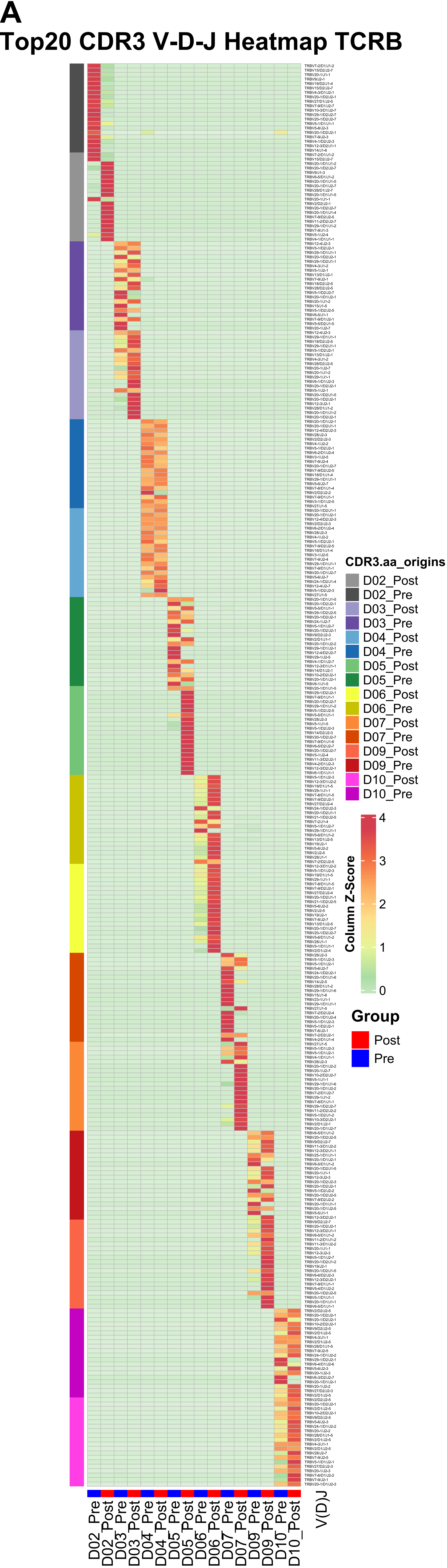
A
Top20 CDR3 V-D-J Heatmap TCRB
CDR3.aa_origins
D02_Post
D02_Pre
D03_Post
D03_Pre
D04_Post
D04_Pre
D05_Post
D05_Pre
D06_Post
D06_Pre
D07_Post
D07_Pre
D09_Post
D09_Pre
D10_Post
D10_Pre
Column Z-Score
4
3
2
1
0
Group
Post
Pre
D02_Pre
D02_Post
D03_Pre
D03_Post
D04_Pre
D04_Post
D05_Pre
D05_Post
D06_Pre
D06_Post
D07_Pre
D07_Post
D09_Pre
D09_Post
D10_Pre
D10_Post
V(D)J

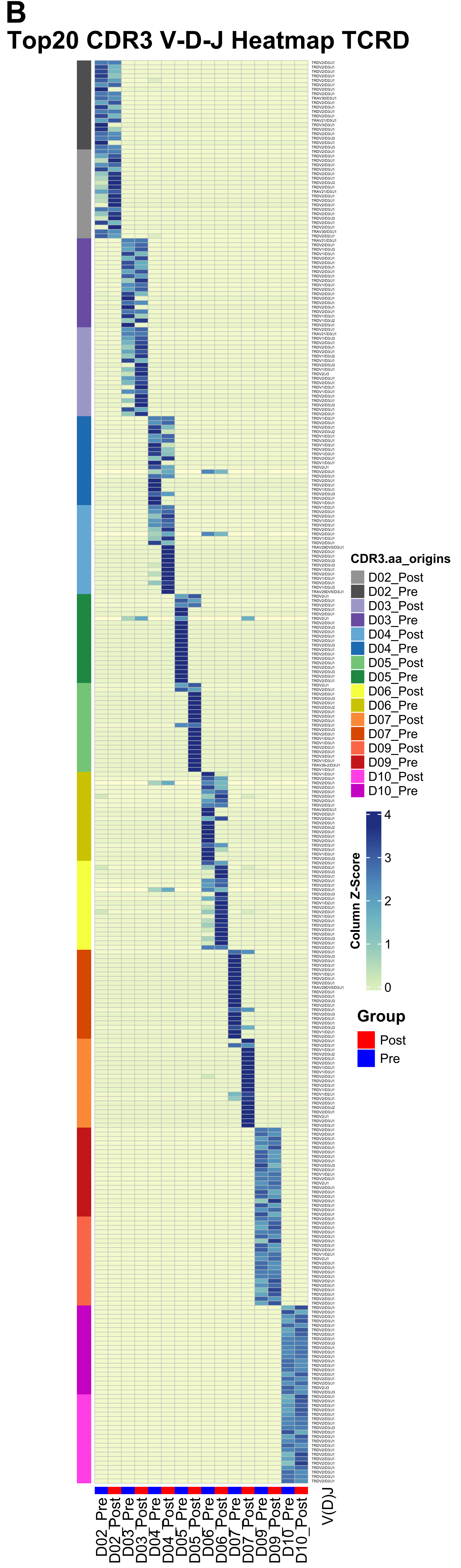
B
Top20 CDR3 V-D-J Heatmap TCRD
CDR3.aa_origins
D02_Post
D02_Pre
D03_Post
D03_Pre
D04_Post
D04_Pre
D05_Post
D05_Pre
D06_Post
D06_Pre
D07_Post
D07_Pre
D09_Post
D09_Pre
D10_Post
D10_Pre
Column Z-Score
4
3
2
1
0
Group
Post
Pre
D02_Pre
D02_Post
D03_Pre
D03_Post
D04_Pre
D04_Post
D05_Pre
D05_Post
D06_Pre
D06_Post
D07_Pre
D07_Post
D09_Pre
D09_Post
D10_Pre
D10_Post
V(D)J
