# Supplementary material for: Impact of G‐CSF on Donor TCR Clonal Diversity and T Cell Function During Donor HSC Mobilisation
Source: Cell Prolif. 2026 Apr 16;59(9):e70213. doi: 10.1111/cpr.70213 (PMC13325648; doi:10.1111/cpr.70213)
Supplement: Supplementary file 5 [file CPR-59-e70213-s006.pdf]

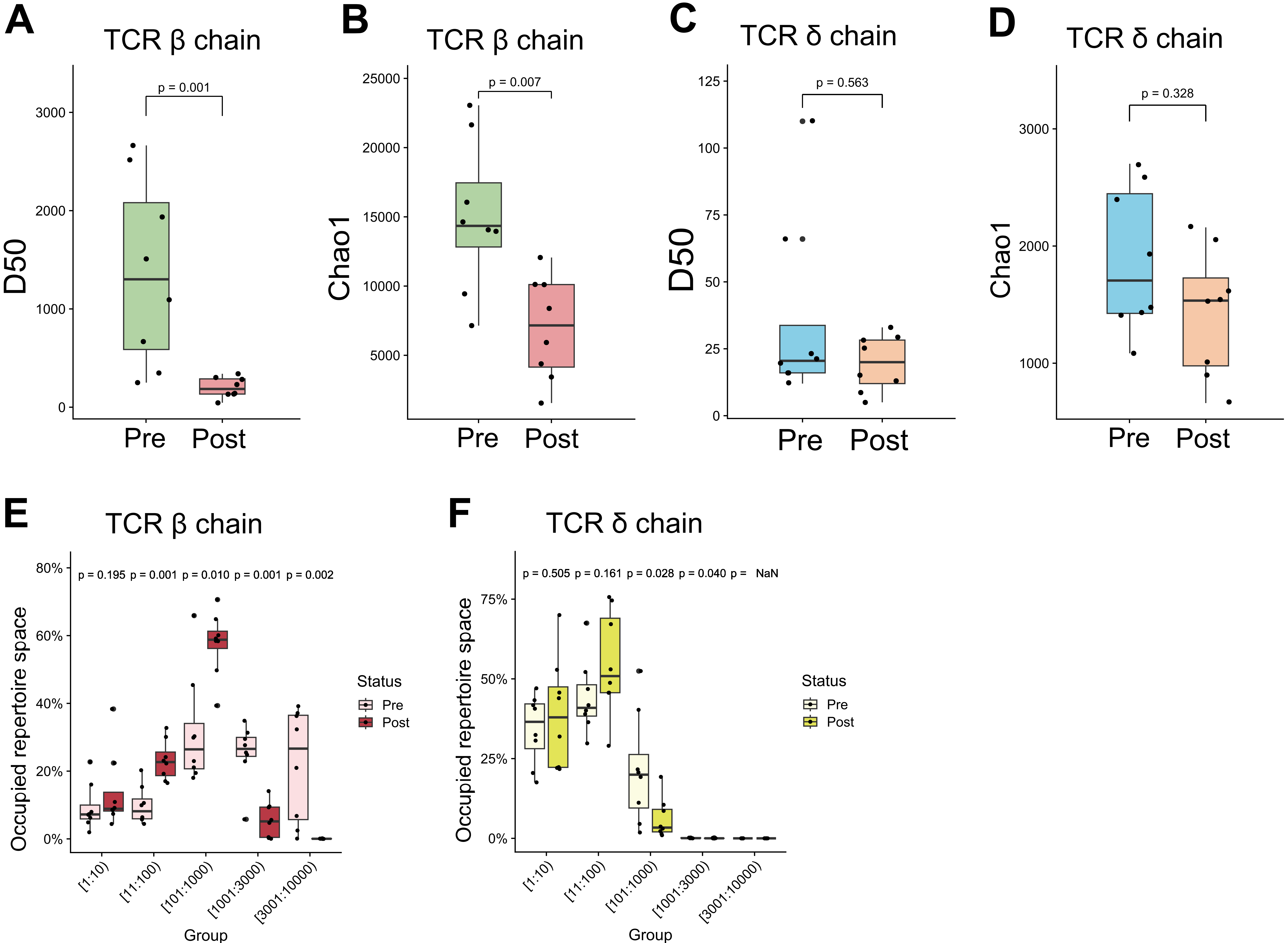
A
TCR β chain
D50
p = 0.001
0
1000
2000
3000
Pre
Post
B
TCR β chain
Chao1
p = 0.007
5000
10000
15000
20000
25000
Pre
Post
C
TCR δ chain
D50
p = 0.563
0
25
50
75
100
125
Pre
Post
D
TCR δ chain
Chao1
p = 0.328
1000
2000
3000
Pre
Post
E
TCR β chain
Occupied repertoire space
p = 0.195 p = 0.001 p = 0.010 p = 0.001 p = 0.002
0%
20%
40%
60%
80%
[1:10)
[11:100)
[101:1000)
[1001:3000)
[3001:10000)
Group
Status
Pre
Post
F
TCR δ chain
Occupied repertoire space
p = 0.505 p = 0.161 p = 0.028 p = 0.040 p = NaN
0%
25%
50%
75%
[1:10)
[11:100)
[101:1000)
[1001:3000)
[3001:10000)
Group
Status
Pre
Post
