# Supplementary material for: Impact of G‐CSF on Donor TCR Clonal Diversity and T Cell Function During Donor HSC Mobilisation
Source: Cell Prolif. 2026 Apr 16;59(9):e70213. doi: 10.1111/cpr.70213 (PMC13325648; doi:10.1111/cpr.70213)
Supplement: Supplementary file 6 [file CPR-59-e70213-s007.pdf]

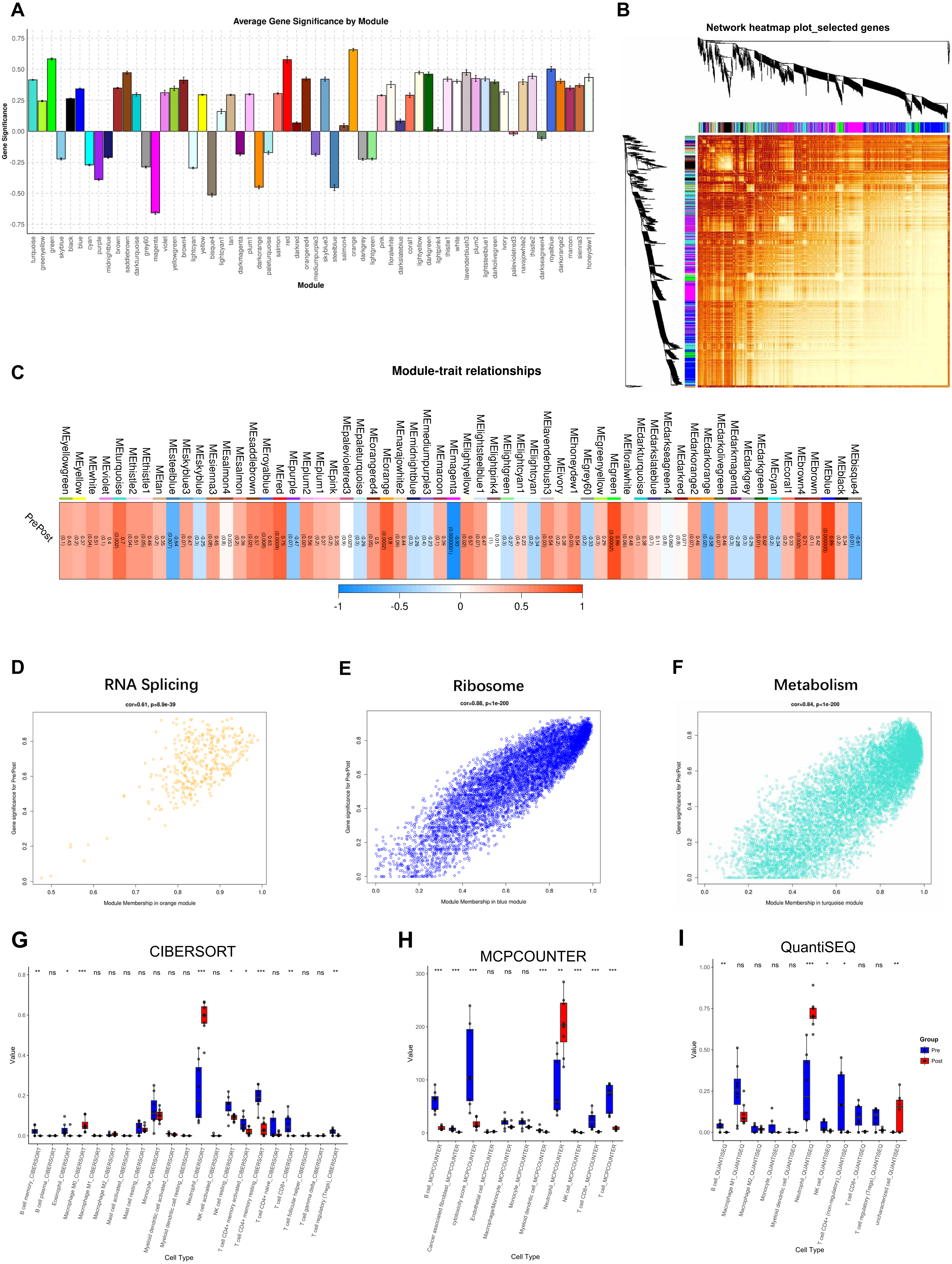
A
Average Gene Significance by Module
Gene Significance
Module
turquoise greenyellow green skyblue black blue cyan purple midnightblue brown saddlebrown darkturquoise grey60 magenta violet yellowgreen brown4 lightcyan yellow bisque4 lightcyan1 tan darkmagenta plum1 darkorange paleturquoise salmon red darkred orangered4 mediumpurple3 skyblue3 steelblue salmon4 orange darkgrey lightgreen pink floralwhite darkslateblue coral1 lightyellow darkgreen lightpink4 thistle1 white lavenderblush3 plum2 lightsteelblue1 darkolivegreen ivory palevioletred3 navajowhite2 thistle2 darkseagreen4 royalblue darkorange2 maroon sienna3 honeydew1
B
Network heatmap plot_selected genes
C
Module-trait relationships
PrePost
MEyellowgreen MEyellow MEwhite MEviolet MEturquoise MEthistle2 MEthistle1 MEtan MEsteelblue MEskyblue3 MEskyblue MEsienna3 MEsalmon4 MEsalmon MEsaddlebrown MEroyalblue MEred MEpurple MEplum2 MEplum1 MEpink MEpalevioletred3 MEpaleturquoise MEorangered4 MEorange MEnavajowhite2 MEmidnightblue MEmediumpurple3 MEmaroon MEmagenta MElightyellow MElightsteelblue1 MElightpink4 MElightgreen MElightcyan1 MElightcyan MElavenderblush3 MEivory MEhoneydew1 MEgrey60 MEgreenyellow MEgreen MEfloralwhite MEdarkturquoise MEdarkslateblue MEdarkseagreen4 MEdarkred MEdarkorange2 MEdarkorange MEdarkolivegreen MEdarkmagenta MEdarkgrey MEdarkgreen MEcyan MEcoral1 MEbrown4 MEbrown MEblue MEblack MEbisque4
D
RNA Splicing
cor=0.61, p=8.9e-39
Gene significance for Pre/Post
Module Membership in orange module
E
Ribosome
cor=0.88, p<1e-200
Gene significance for Pre/Post
Module Membership in blue module
F
Metabolism
cor=0.84, p<1e-200
Gene significance for Pre/Post
Module Membership in turquoise module
G
CIBERSORT
Value
Cell Type
H
MCPCOUNTER
Value
Cell Type
I
QuantiSEQ
Value
Cell Type
Group
Pre
Post
