# Supplementary material for: Impact of G‐CSF on Donor TCR Clonal Diversity and T Cell Function During Donor HSC Mobilisation
Source: Cell Prolif. 2026 Apr 16;59(9):e70213. doi: 10.1111/cpr.70213 (PMC13325648; doi:10.1111/cpr.70213)
Supplement: Supplementary file 7 [file CPR-59-e70213-s008.pdf]

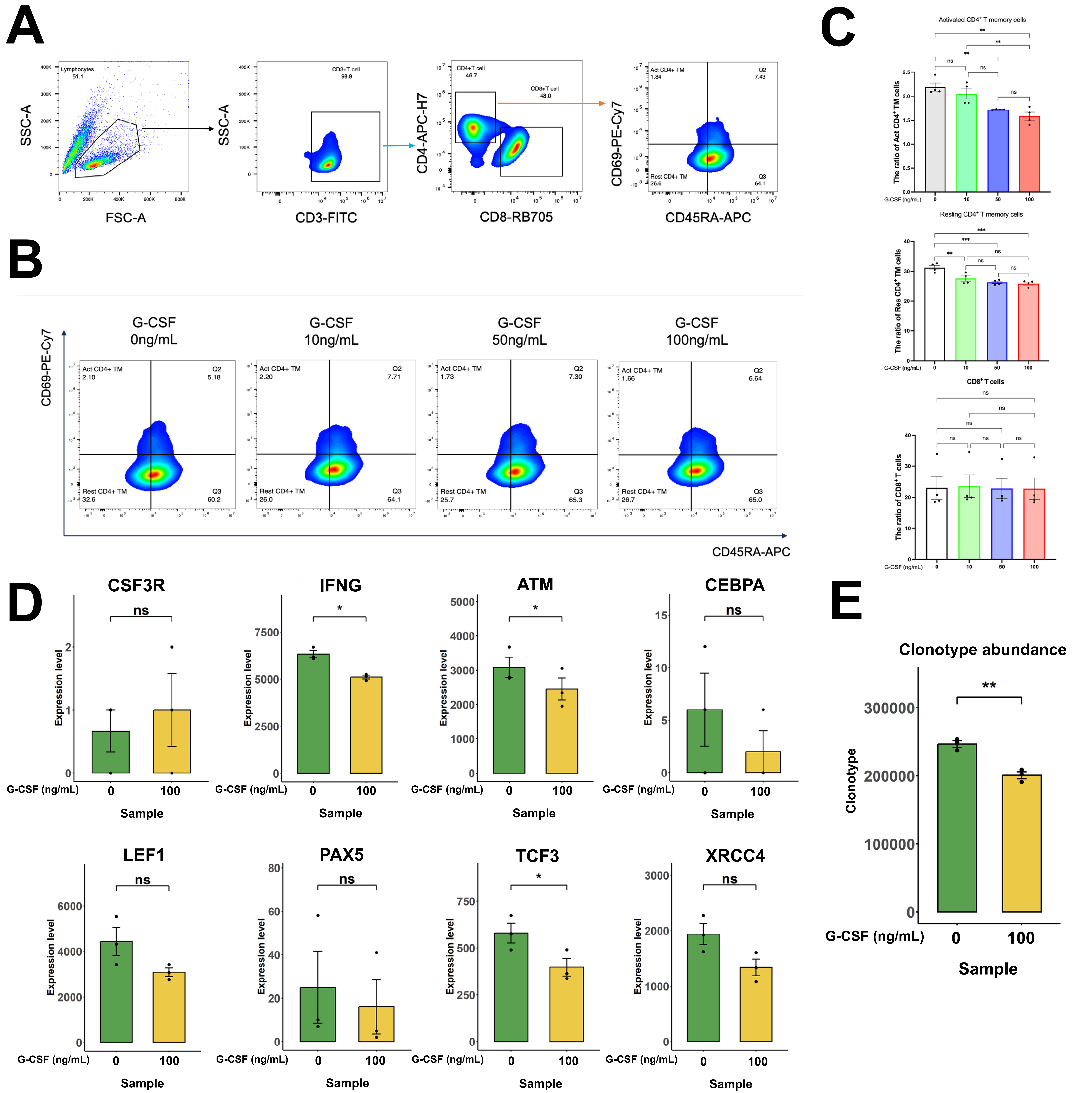

A
Lymphocytes 51.1
SSC-A
FSC-A
CD3+T cell 98.9
CD3-FITC
CD4+T cell 46.7
CD8+T cell 48.0
CD4-APC-H7
CD8-RB705
Act CD4+ TM 1.84
Q2 7.43
Rest CD4+ TM 26.6
Q3 64.1
CD69-PE-Cy7
CD45RA-APC
B
G-CSF 0ng/mL
G-CSF 10ng/mL
G-CSF 50ng/mL
G-CSF 100ng/mL
Act CD4+ TM 2.10
Q2 5.18
Rest CD4+ TM 32.6
Q3 60.2
Act CD4+ TM 2.20
Q2 7.71
Rest CD4+ TM 26.0
Q3 64.1
Act CD4+ TM 1.73
Q2 7.30
Rest CD4+ TM 25.7
Q3 65.3
Act CD4+ TM 1.66
Q2 6.64
Rest CD4+ TM 26.7
Q3 65.0
CD69-PE-Cy7
CD45RA-APC
C
Activated CD4+ T memory cells
The ratio of Act CD4+ TM cells
Resting CD4+ T memory cells
The ratio of Res CD4+ TM cells
CD8+ T cells
The ratio of CD8+ T cells
G-CSF (ng/mL) 0 10 50 100
D
CSF3R
IFNG
ATM
CEBPA
LEF1
PAX5
TCF3
XRCC4
Expression level
G-CSF (ng/mL) 0 100
Sample
E
Clonotype abundance
Clonotype
G-CSF (ng/mL) 0 100
Sample
