# Supplementary material for: Impact of G‐CSF on Donor TCR Clonal Diversity and T Cell Function During Donor HSC Mobilisation
Source: Cell Prolif. 2026 Apr 16;59(9):e70213. doi: 10.1111/cpr.70213 (PMC13325648; doi:10.1111/cpr.70213)
Supplement: Supplementary file 9 [file CPR-59-e70213-s010.pdf]

TCR β chain

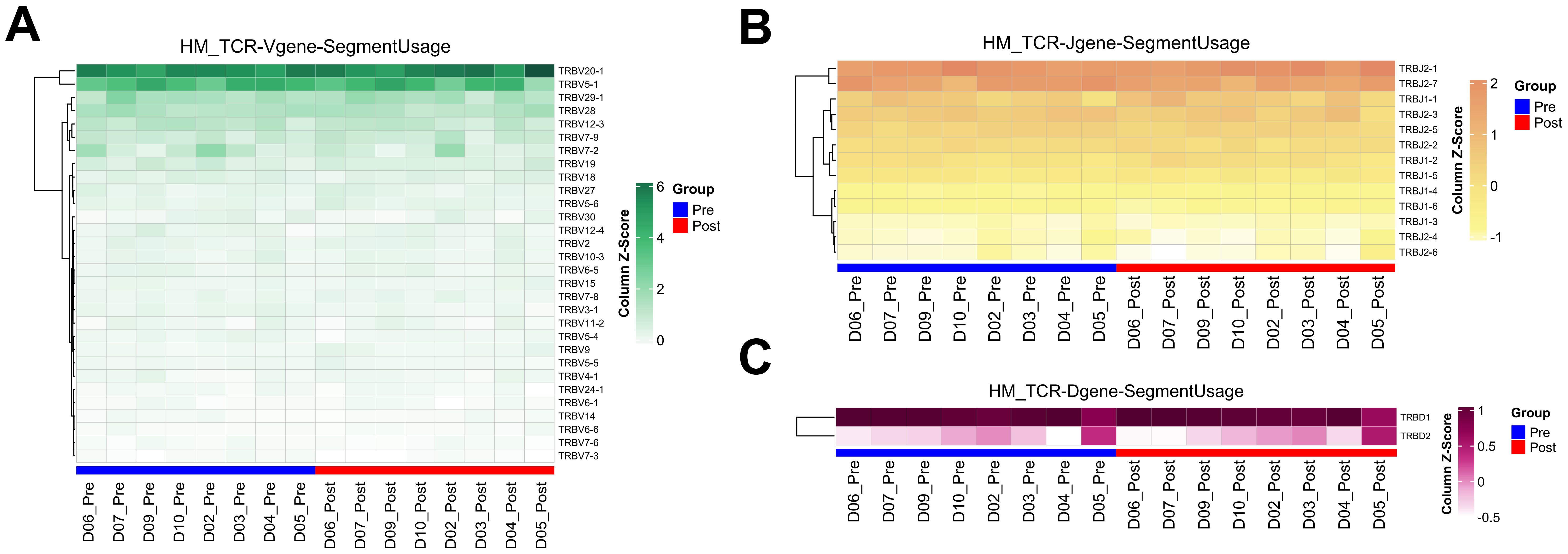
A
HM_TCR-Vgene-SegmentUsage
TRBV20-1
TRBV5-1
TRBV29-1
TRBV28
TRBV12-3
TRBV7-9
TRBV7-2
TRBV19
TRBV18
TRBV27
TRBV5-6
TRBV30
TRBV12-4
TRBV2
TRBV10-3
TRBV6-5
TRBV15
TRBV7-8
TRBV3-1
TRBV11-2
TRBV5-4
TRBV9
TRBV5-5
TRBV4-1
TRBV24-1
TRBV6-1
TRBV14
TRBV6-6
TRBV7-6
TRBV7-3
Column Z-Score
6
4
2
0
Group
Pre
Post
D06_Pre
D07_Pre
D09_Pre
D10_Pre
D02_Pre
D03_Pre
D04_Pre
D05_Pre
D06_Post
D07_Post
D09_Post
D10_Post
D02_Post
D03_Post
D04_Post
D05_Post
B
HM_TCR-Jgene-SegmentUsage
TRBJ2-1
TRBJ2-7
TRBJ1-1
TRBJ2-3
TRBJ2-5
TRBJ2-2
TRBJ1-2
TRBJ1-5
TRBJ1-4
TRBJ1-6
TRBJ1-3
TRBJ2-4
TRBJ2-6
Column Z-Score
2
1
0
-1
Group
Pre
Post
D06_Pre
D07_Pre
D09_Pre
D10_Pre
D02_Pre
D03_Pre
D04_Pre
D05_Pre
D06_Post
D07_Post
D09_Post
D10_Post
D02_Post
D03_Post
D04_Post
D05_Post
C
HM_TCR-Dgene-SegmentUsage
TRBD1
TRBD2
Column Z-Score
1
0.5
0
-0.5
Group
Pre
Post
D06_Pre
D07_Pre
D09_Pre
D10_Pre
D02_Pre
D03_Pre
D04_Pre
D05_Pre
D06_Post
D07_Post
D09_Post
D10_Post
D02_Post
D03_Post
D04_Post
D05_Post
