# Supplementary material for: Impact of G‐CSF on Donor TCR Clonal Diversity and T Cell Function During Donor HSC Mobilisation
Source: Cell Prolif. 2026 Apr 16;59(9):e70213. doi: 10.1111/cpr.70213 (PMC13325648; doi:10.1111/cpr.70213)
Supplement: Supplementary file 10 [file CPR-59-e70213-s011.pdf]

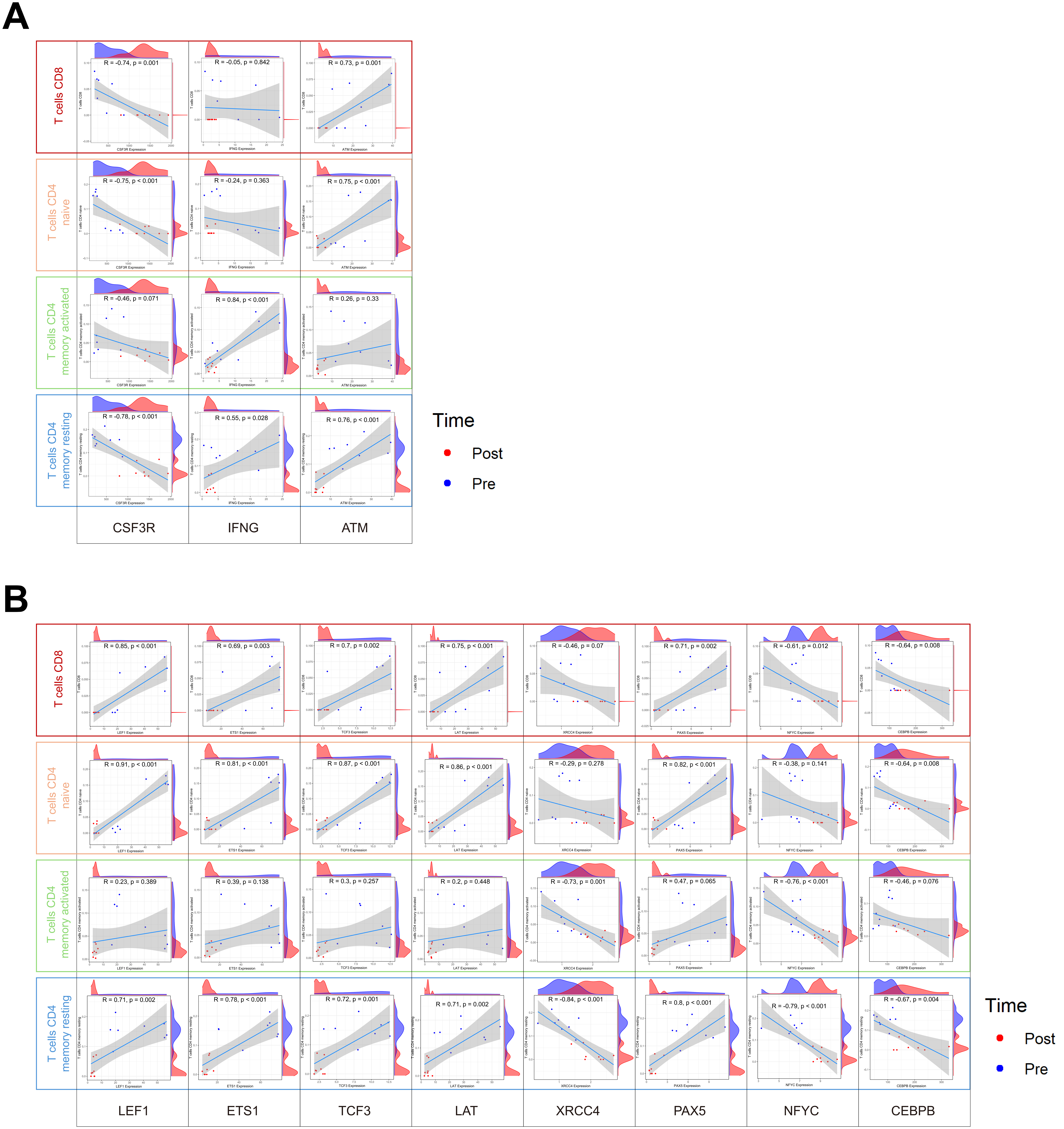
A
T cells CD8
T cells CD4 naive
T cells CD4 memory activated
T cells CD4 memory resting
R = -0.74, p = 0.001
R = -0.05, p = 0.842
R = 0.73, p = 0.001
R = -0.75, p < 0.001
R = -0.24, p = 0.363
R = 0.75, p < 0.001
R = -0.46, p = 0.071
R = 0.84, p < 0.001
R = 0.26, p = 0.33
R = -0.78, p < 0.001
R = 0.55, p = 0.028
R = 0.76, p < 0.001
CSF3R
IFNG
ATM
Time
Post
Pre
B
T cells CD8
T cells CD4 naive
T cells CD4 memory activated
T cells CD4 memory resting
R = 0.85, p < 0.001
R = 0.69, p = 0.003
R = 0.7, p = 0.002
R = 0.75, p < 0.001
R = -0.46, p = 0.07
R = 0.71, p = 0.002
R = -0.61, p = 0.012
R = -0.64, p = 0.008
R = 0.91, p < 0.001
R = 0.81, p < 0.001
R = 0.87, p < 0.001
R = 0.86, p < 0.001
R = -0.29, p = 0.278
R = 0.82, p < 0.001
R = -0.38, p = 0.141
R = -0.64, p = 0.008
R = 0.23, p = 0.389
R = 0.39, p = 0.138
R = 0.3, p = 0.257
R = 0.2, p = 0.448
R = -0.73, p = 0.001
R = 0.47, p = 0.065
R = -0.76, p < 0.001
R = -0.46, p = 0.076
R = 0.71, p = 0.002
R = 0.78, p < 0.001
R = 0.72, p = 0.001
R = 0.71, p = 0.002
R = -0.84, p < 0.001
R = 0.8, p < 0.001
R = -0.79, p < 0.001
R = -0.67, p = 0.004
LEF1
ETS1
TCF3
LAT
XRCC4
PAX5
NFYC
CEBPB
Time
Post
Pre
