# Supplementary material for: Impact of G‐CSF on Donor TCR Clonal Diversity and T Cell Function During Donor HSC Mobilisation
Source: Cell Prolif. 2026 Apr 16;59(9):e70213. doi: 10.1111/cpr.70213 (PMC13325648; doi:10.1111/cpr.70213)
Supplement: Supplementary file 11 [file CPR-59-e70213-s005.docx]

Supplementary Table 1. Oligonucleotide sequences for qPCR.

| Primer | Oligonucleotide sequences |
| --- | --- |
| CSF3R-F | 5’- TGGAGCTGAGAACTACCGAA -3’ |
| CSF3R-R | 5’- CCACATAACCTTGGATCCGT -3’ |
| IFNG-F | 5’-CATGTATTGCTTTGCGTTGG -3’ |
| IFNG-R | 5’-AAGAGTGTGGAGACCATCAA -3’ |
| ATM-F | 5’- ATGCTGTTCTCAGACTGACG -3’ |
| ATM-R | 5’-TGACAGCCAAAGTCTTGAGG -3’ |
| CEBPA-F | 5’- CAAGAAGTCGGTGGACAAGA -3’ |
| CEBPA-R | 5’- GGTCATTGTCACTGGTCAGC -3’ |
| LEF1-F | 5’- CGACCCATACATGTCAAATGG -3’ |
| LEF1-R | 5’- GAGAAAAGTGCTCGTCACTG -3’ |
| PAX5-F | 5’- CTTGCTCATCAAGGTGTCAG -3’ |
| PAX5-R | 5’- CCTCCAATTACCCCAGGCTT -3’ |
| TCF3-F | 5’- CCGACTCCTACAGTGGGCTA -3’ |
| TCF3-R | 5’- CGCTGACGTGTTCTCCTCG -3’ |
| XRCC4-F | 5’- AGCAGGACCAGCTGATGTAT -3’ |
| XRCC4-R | 5’- GCAATGGTGTCCAAGCAATAAC -3’ |
| ACTB-F | 5’-TTGTTACAGGAAGTCCCTTGCC -3’ |
| ACTB-R | 5’-ATGCTATCACCTCCCCTGTGTG -3’ |

Supplementary Table 2 Changes in the distribution of TCR Vβ chain clonotypes (Top 500) before and after G-CSF mobilization (Pre/Post)

| **CDR3 Motif** | **Centroid** | **Motif-Constraint** | **Radius** | **Meta-Clonotype** |
| --- | --- | --- | --- | --- |
| **Meta-clonotype Pre** |  |  |  |  |
|  | TRBV6-3*01/TRBJ2-2*01  CASSYETPANTGELFF | (SY[REG]?[GPSTV][P]?[ASV]NTGEL) | 36 | TRBV6-3*01+ CASSYETPANTGELFF+  36+  (SY[REG]?[GPSTV][P]?[ASV]NTGEL) |
|  | TRBV6-3*01/TRBJ2-2*01  CASSYGSANTGELFF | (SY[RGV][PST][ASV]N[T]?GEL) | 28 | TRBV6-3*01+ CASSYGSANTGELFF+  28+  (SY[RGV][PST][ASV]N[T]?GEL) |
|  | TRBV11-3*01/TRBJ1-1*01  CASSFGENTEAFF | (S[LF][AG][DQEGL][N]?TEA) | 30 | TRBV11-3*01+ CASSFGENTEAFF+  30+  (S[LF][AG][DQEGL][N]?TEA) |
|  | TRBV11-3*01/TRBJ1-1*01  CASSFALNTEAFF | (S[QF][AQGS]?[AEGLY][NT]?TEA) | 36 | TRBV11-3*01+ CASSFALNTEAFF +  36+  (S[QF][AQGS]?[AEGLY][NT]?TEA) |
|  | TRBV6-3*01/TRBJ2-7*01  CASVYLGTSGAEQYF | ([SV]Y[L]?GTSG[AY]EQ) | 36 | TRBV6-3*01+ CASVYLGTSGAEQYF +  36+  ([SV]Y[L]?GTSG[AY]EQ) |
|  | TRBV6-1*01/TRBJ2-2*01  CASSEGDTGELFF | (S[ET][QG][DE]TGEL) | 28 | TRBV6-1*01+ CASSEGDTGELFF +  28+  (S[ET][QG][DE]TGEL) |
|  | TRBV6-1*01/TRBJ2-2*01  CASSTGDTGELFF | (S[ET][QGT][NDE]TGEL) | 32 | TRBV6-1*01+ CASSTGDTGELFF +  32+  (S[ET][QGT][NDE]TGEL) |
|  | TRBV6-3*01/TRBJ1-5*01  CASSSGRLSGNQPQHF | (SSG[RT][LY]S[G]?NQPQ) | 36 | TRBV6-3*01+ CASSSGRLSGNQPQHF+  36+  (SSG[RT][LY]S[G]?NQPQ) |
|  | TRBV6-3*01/TRBJ2-2*01  CASSRVPGGTGELFF | ([RS][RY][TV][P]?[AG]?[NG]TGEL) | 36 | TRBV6-3*01+ CASSRVPGGTGELFF+  36+  ([RS][RY][TV][P]?[AG]?[NG]TGEL) |
|  | TRBV6-3*01/TRBJ2-2*01  CASSSGTGHTGELFF | (S[SY]GT[GV][NH]TGEL) | 34 | TRBV6-3*01+ CASSSGTGHTGELFF+  34+  (S[SY]GT[GV][NH]TGEL) |
|  | TRBV6-3*01/TRBJ2-3*01  CASSPGTSETDTQYF | (S[PY]G[T]?[RS][DE]?TDTQ) | 34 | TRBV6-3*01+ CASSPGTSETDTQYF+  34+  (S[PY]G[T]?[RS][DE]?TDTQ) |
| **Meta-clonotype Post** |  |  |  |  |
|  | TRBV6-1*01/TRBJ2-7*01  CASVYLGTSGAEQYF | (VYLGTSGAEQ) | 36 | TRBV6-1*01+ CASVYLGTSGAEQYF+  36+  (VYLGTSGAEQ) |
|  | TRBV6-3*01/TRBJ1-5*01  CASSSGRLSGNRPQHF | (SSGRLSGN[RQ]PQ) | 36 | TRBV6-3*01+ CASSSGRLSGNRPQHF+  36+  (SSGRLSGN[RQ]PQ) |
|  | TRBV12-3*01/TRBJ2-1*01  CASSFLSGGEYNEQFF | (SFLSGGEYNEQ) | 26 | TRBV12-3*01+ CASSFLSGGEYNEQFF+  26+  (SFLSGGEYNEQ) |
|  | TRBV6-3*01/TRBJ1-5*01  CASSPWTGDQPQHF | (SPWTGDQPQ) | 20 | TRBV6-3*01+ CASSPWTGDQPQHF +  20+  (SPWTGDQPQ) |
|  | TRBV6-3*01/TRBJ2-5*01  CASSLGTAGTQYF | (SLGTAGTQ) | 34 | TRBV6-3*01+ CASSLGTAGTQYF +  34+  (SLGTAGTQ) |
|  | TRBV7-6*01/TRBJ2-2*01  CASSLDRVGTGELFF | (SLDRVGTGEL) | 34 | TRBV7-6*01+ CASSLDRVGTGELFF +  34+  (SLDRVGTGEL) |
|  | TRBV28*01/TRBJ1-5*01  CASSFTVLSGNQPQHF | (SFTVLSGNQPQ) | 34 | TRBV28*01+ CASSFTVLSGNQPQHF+  34+  (SFTVLSGNQPQ) |
|  | TRBV6-3*01/TRBJ2-5*01  CASSFADRQETQYF | (SFADRQETQ) | 26 | TRBV6-3*01+ CASSFADRQETQYF +  26+  (SFADRQETQ) |
|  | TRBV6-3*01/TRBJ2-2*01  CASSSTRPGELFF | (SSTRPGEL) | 22 | TRBV6-3*01+ CASSSTRPGELFF+  22+  (SSTRPGEL) |
|  | TRBV7-2*01/TRBJ1-1*01  CASSPQQGGFTEAFF | (SP[RQ]QGG[F]?TEA) | 36 | TRBV7-2*01+ CASSPQQGGFTEAFF+  36+  (SP[RQ]QGG[F]?TEA) |
|  | TRBV28*01/TRBJ1-5*01  CASSPGGVLEPQHF | (SPGG[AV][QL]E[PT]Q) | 34 | TRBV28*01+ CASSPGGVLEPQHF+  34+  (SPGG[AV][QL]E[PT]Q) |
|  | TRBV7-9*01/TRBJ1-1*01  CASIQGISTEAFF | (IQGISTEA) | 34 | TRBV7-9*01+ CASIQGISTEAFF +  34+  (IQGISTEA) |
|  | TRBV6-3*01/TRBJ1-5*01  CASSYGQAAQPQHF | (SYGQAAQPQ) | 34 | TRBV6-3*01+ CASSYGQAAQPQHF +  34+  (SYGQAAQPQ) |
|  | TRBV6-3*01/TRBJ2-1*01  CASSYGGTGSHEQFF | (SYGG[RT]G[S]?HEQ) | 26 | TRBV6-3*01+ CASSYGGTGSHEQFF +  26+  (SYGG[RT]G[S]?HEQ) |
|  | TRBV12-3*01/TRBJ1-1*01  CASTWTGQTEAFF | ([RT][QSW][T]?[G]?[NQG]TEA) | 34 | TRBV12-3*01+ CASTWTGQTEAFF+  34+  ([RT][QSW][T]?[G]?[NQG]TEA) |
|  | TRBV12-3*01/TRBJ1-1*01  CASTQTGINTEAFF | ([ST][NQW]?[RLT]G[IV]?[NQ]TEA) | 36 | TRBV12-3*01+ CASTQTGINTEAFF +  36+  ([ST][NQW]?[RLT]G[IV]?[NQ]TEA) |
|  | TRBV12-3*01/TRBJ1-5*01  CASSLTGAGQPQHF | (SL[QT]G[AG][DG]QPQ) | 30 | TRBV12-3*01+ CASSLTGAGQPQHF +  30+  (SL[QT]G[AG][DG]QPQ) |
|  | TRBV20-1*01/TRBJ2-1*01  CSAGTHNEQFF | (GT[DH]NEQ) | 16 | TRBV20-1*01+ CSAGTHNEQFF+  16+  (GT[DH]NEQ) |

Supplementary Table 3 Distribution of TCR Vβ chain clonotypes (Top 200) before G-CSF mobilization

| **CDR3 Motif** | **Centroid** | **Motif-Constraint** | **Radius** | **Meta-Clonotype** |
| --- | --- | --- | --- | --- |
|  | TRBV6-3*01/TRBJ2-2*01  CASSYGSANTGELFF | (SY[GV][PST][ASV]NTGEL) | 28 | TRBV6-3*01+ CASSYGSANTGELFF+  28+  (SY[GV][PST][ASV]NTGEL) |
|  | TRBV28*01/TRBJ2-7*01  CASSLWALGEQYF | (SL[TW][AD][L]?GEQ) | 28 | TRBV6-3*01+ CASSLWALGEQYF+  28+  (SL[TW][AD][L]?GEQ) |
|  | TRBV6-3*01/TRBJ2-2*01  CASSSGTGHTGELFF | (S[SY]GT[GV][NH]TGEL) | 34 | TRBV11-3*01+ CASSSGTGHTGELFF+  34+  (S[SY]GT[GV][NH]TGEL) |
|  | TRBV6-3*01/TRBJ2-2*01  CASSRVPGGTGELFF | (S[RY]V[P]?[AG]?[NG]TGEL) | 36 | TRBV11-3*01+ CASSRVPGGTGELFF +  36+  (S[RY]V[P]?[AG]?[NG]TGEL) |
|  | TRBV6-3*01/TRBJ2-7*01  CASSPGTSETDTQYF | (S[PY]G[T]?[RS][DE]?TDTQ) | 34 | TRBV6-3*01+ CASSPGTSETDTQYF +  34+  (S[PY]G[T]?[RS][DE]?TDTQ) |
