# Supplementary figures and images for: Impact of G‐CSF on Donor TCR Clonal Diversity and T Cell Function During Donor HSC Mobilisation
Source: Cell Prolif. 2026 Apr 16:e70213. Online ahead of print. doi: 10.1111/cpr.70213 (PMC13325648; doi:10.1111/cpr.70213)

**A**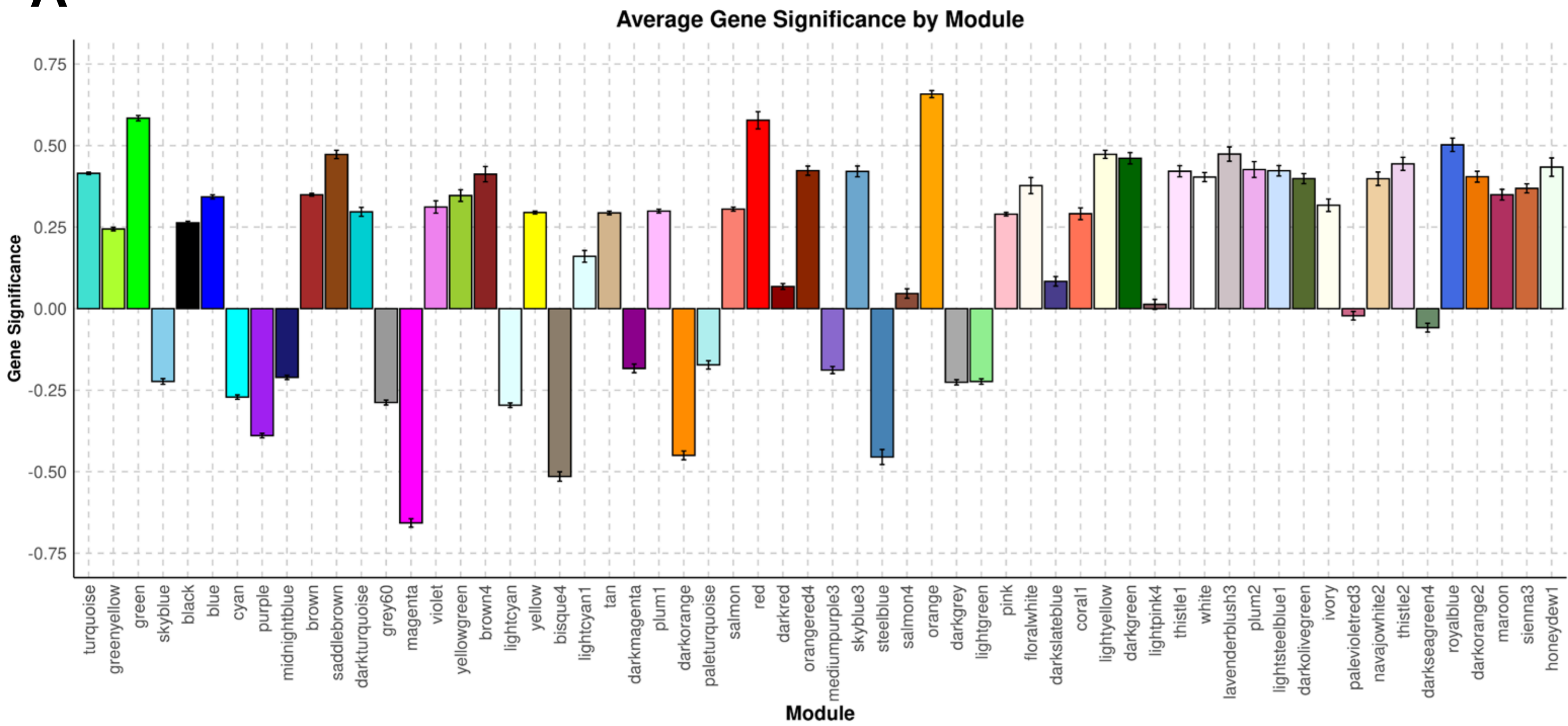**B**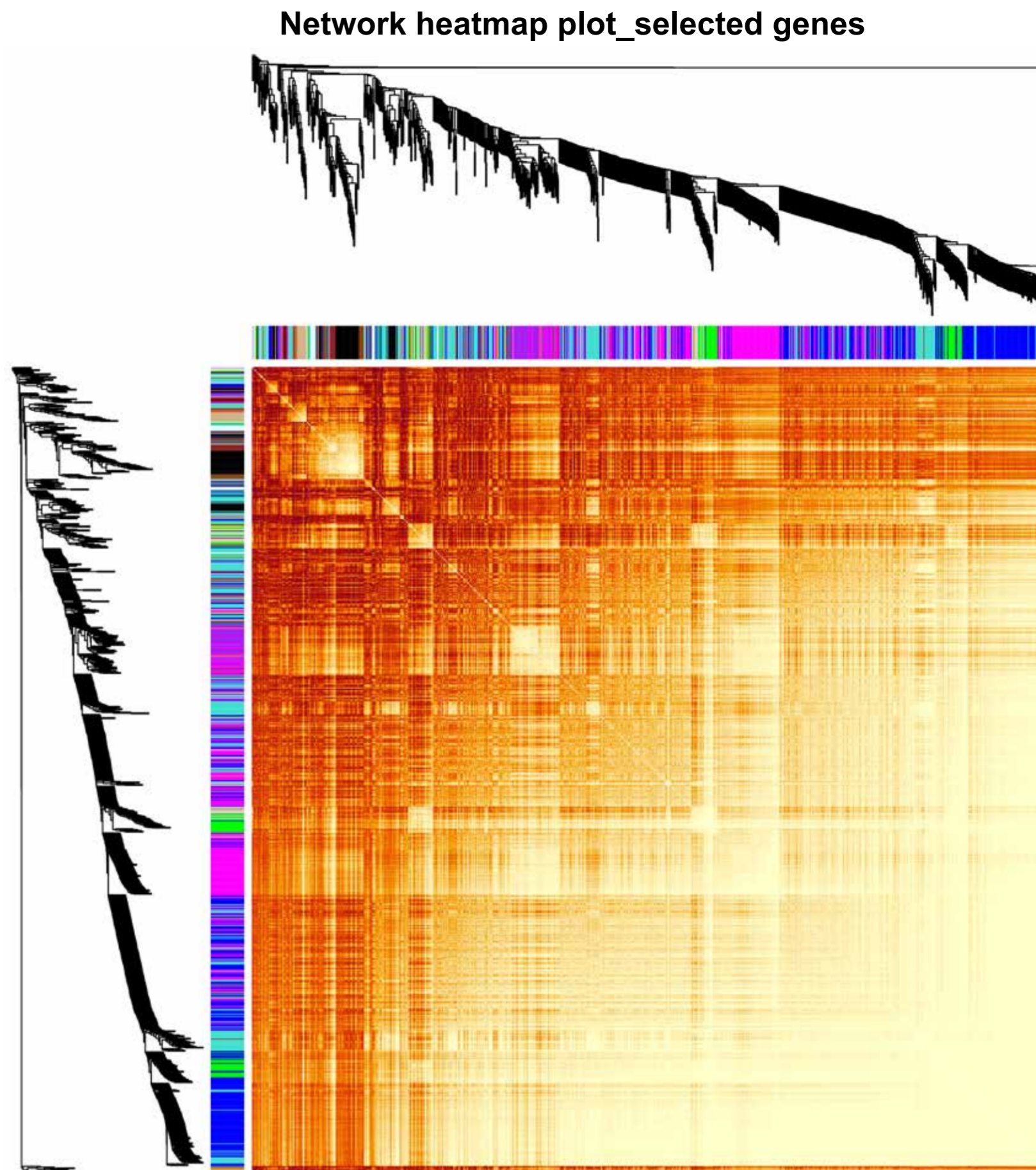**C**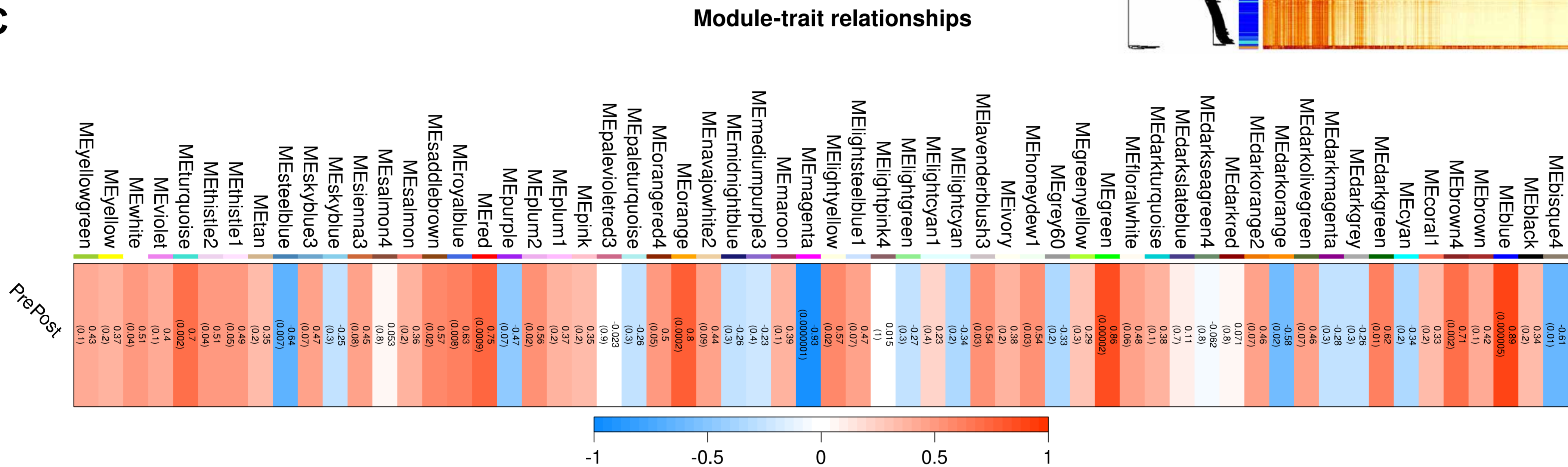**D**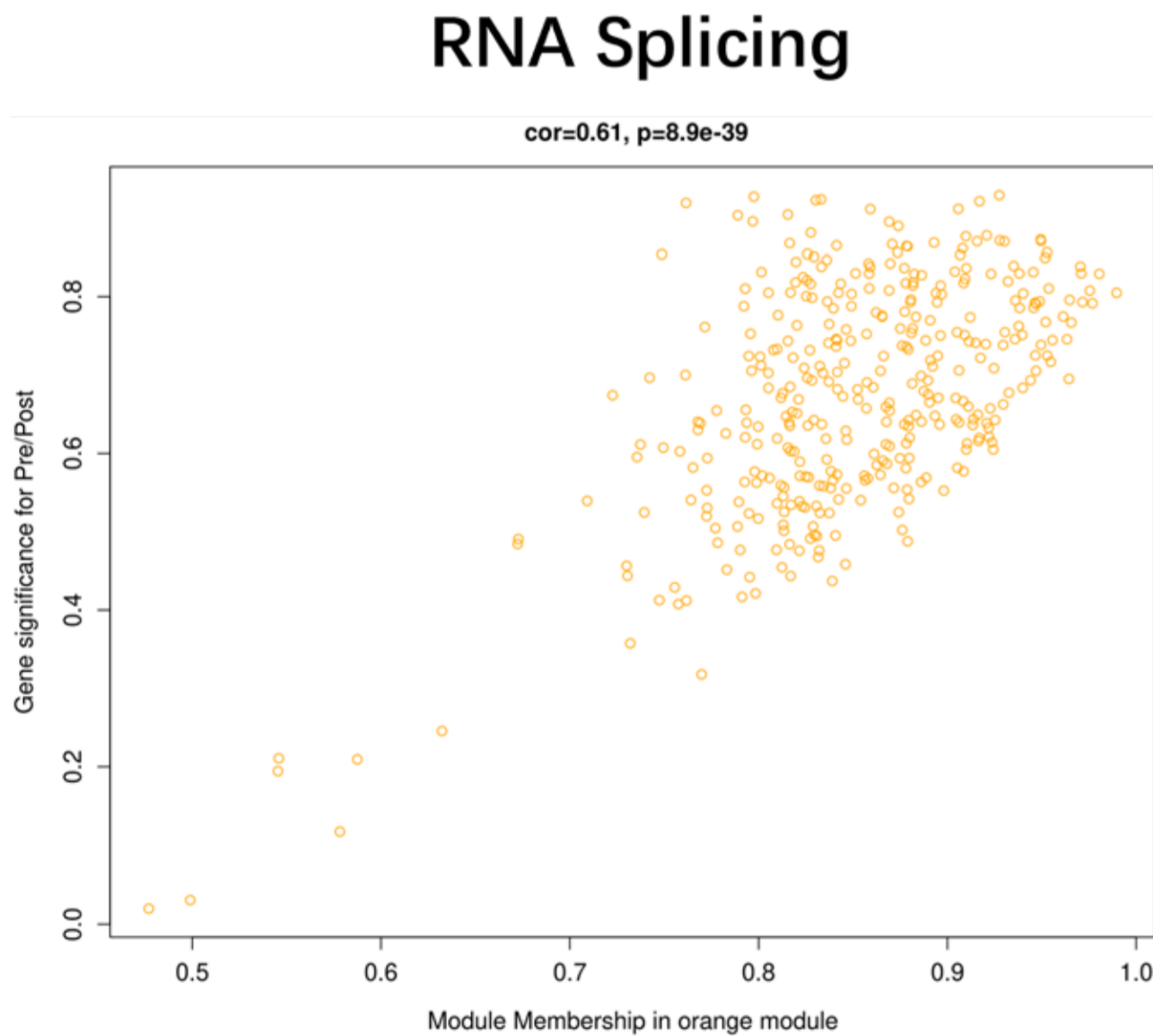**E**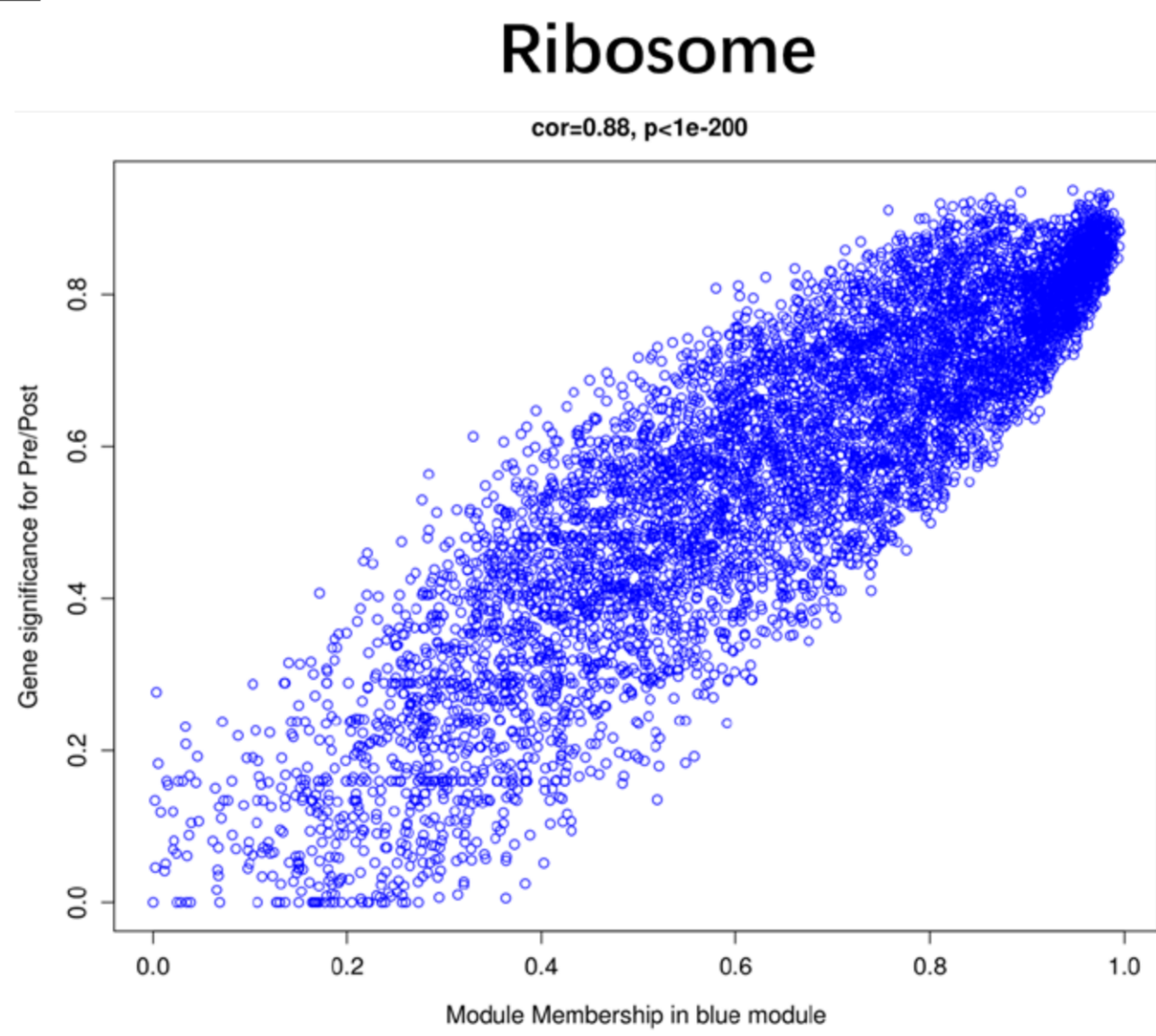**F**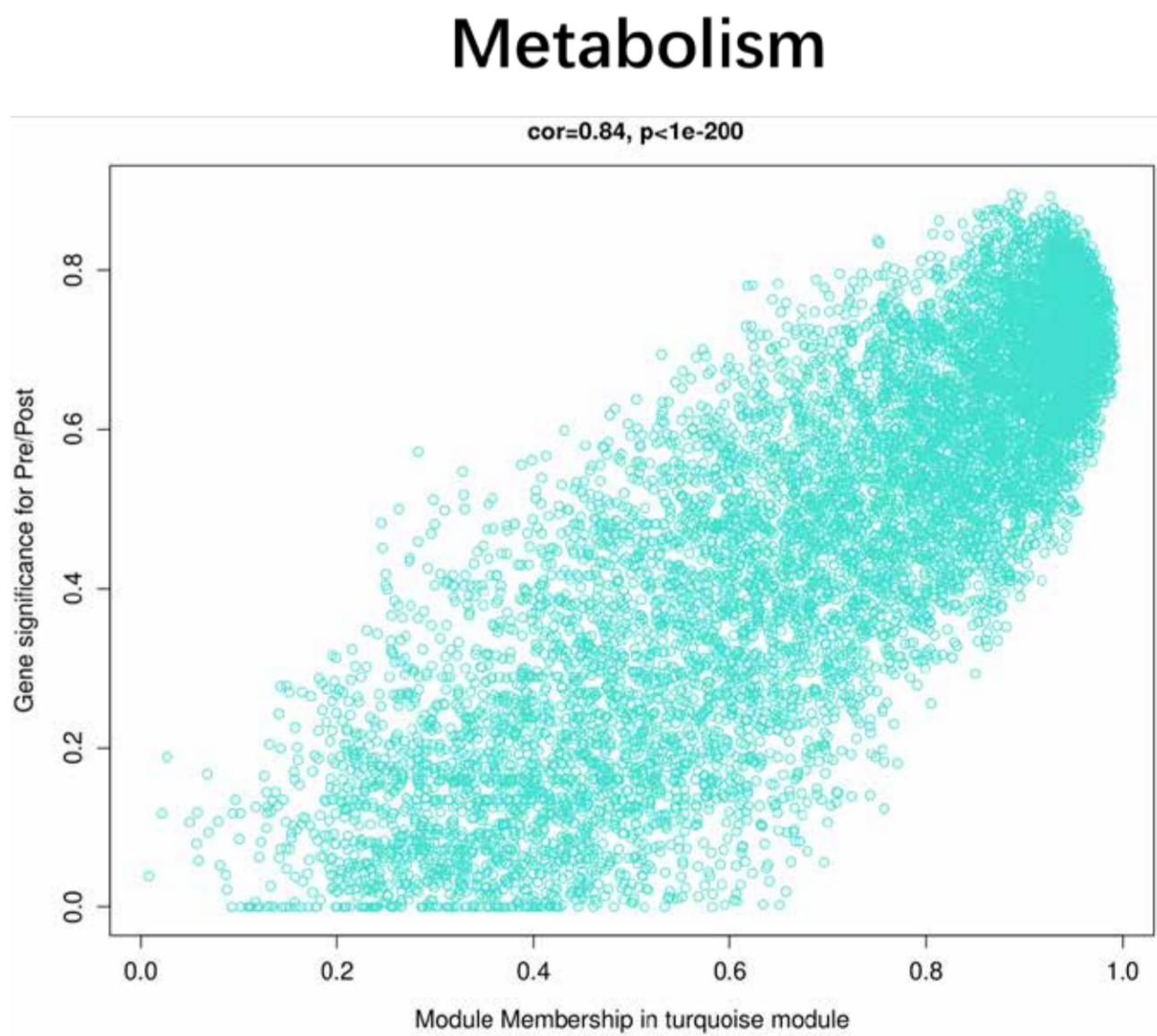**G**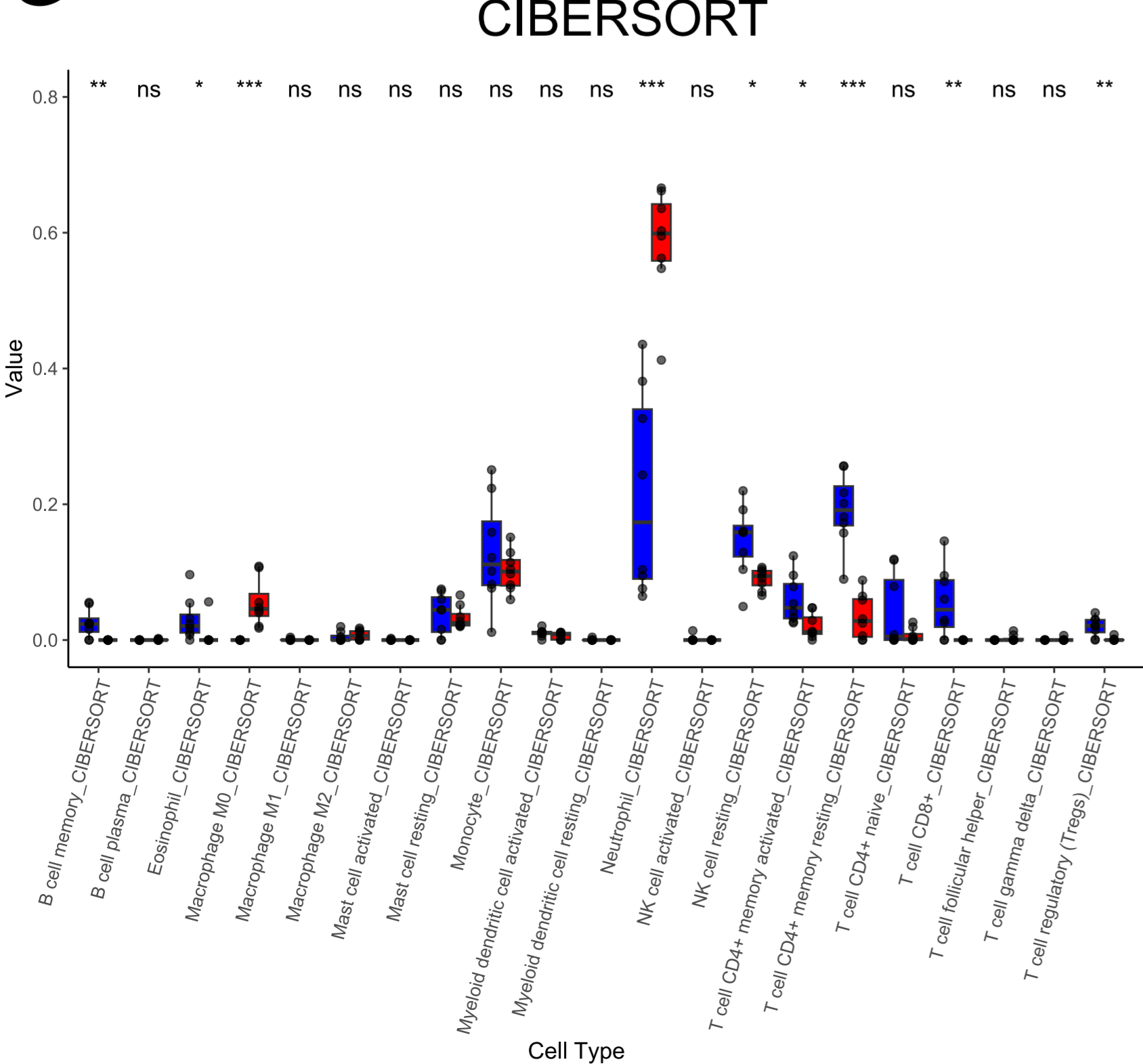**H**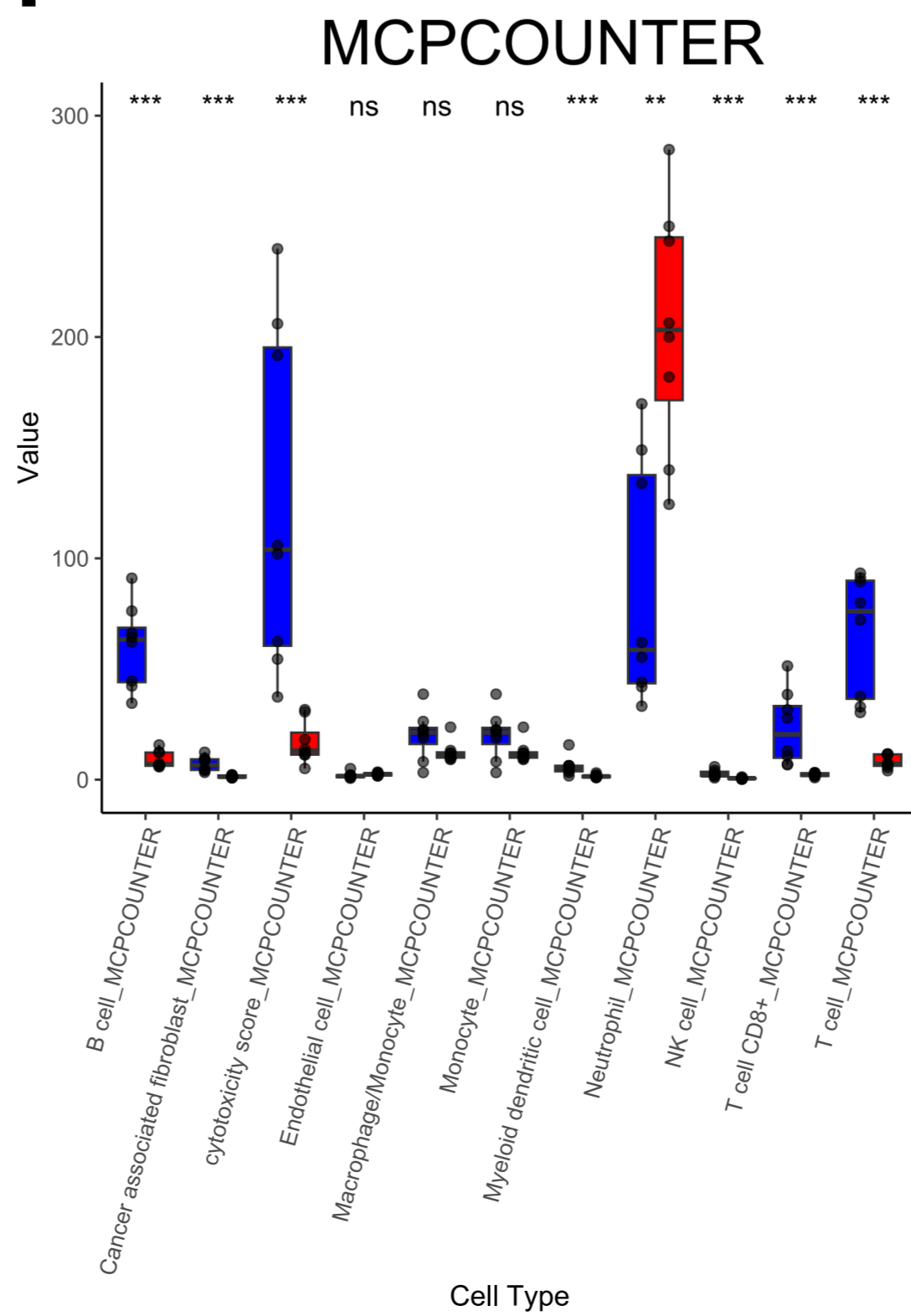**I**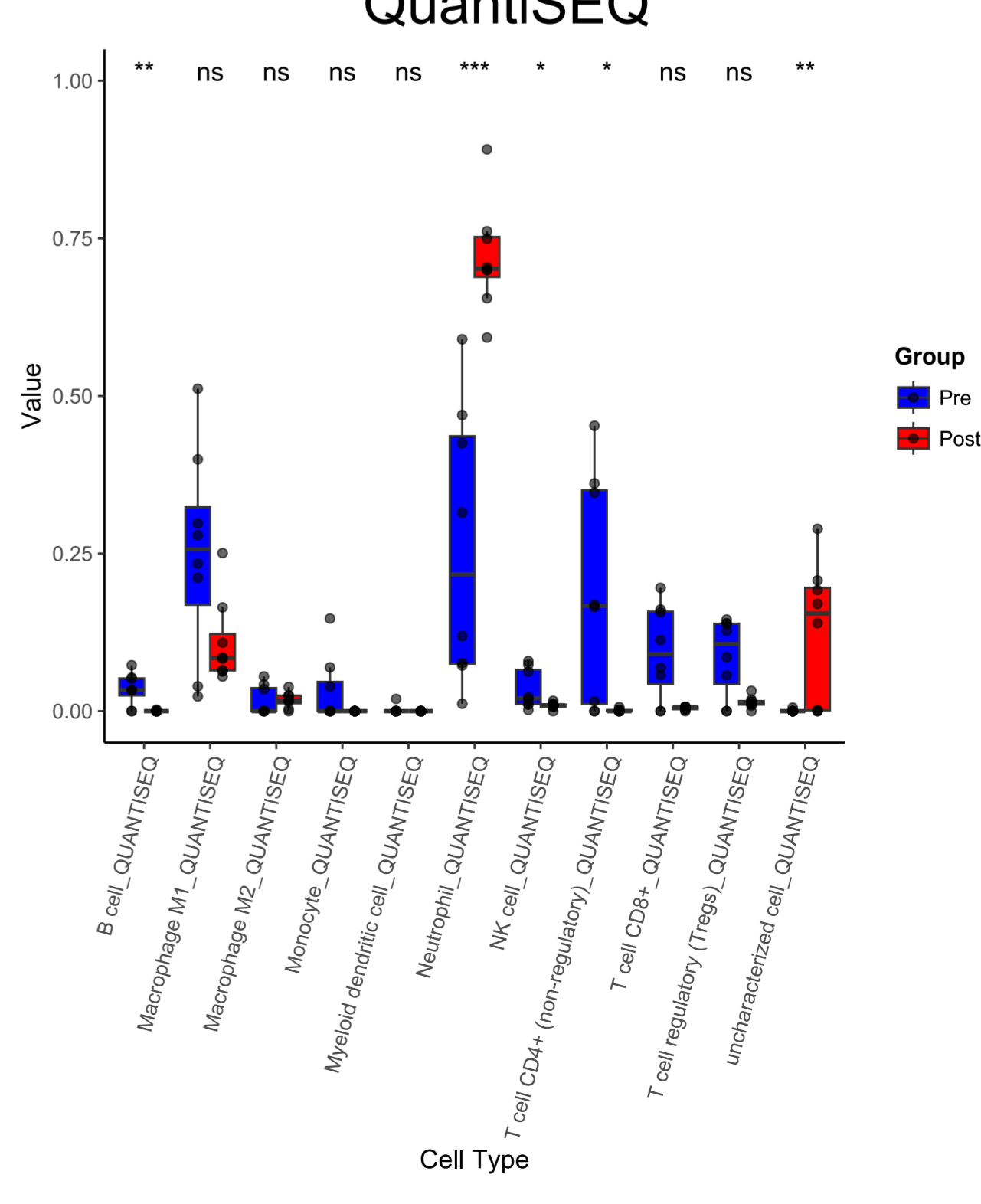

Supplement: Supplementary file 2 — Figure S1: WGCNA module‐trait and gene correlation before and after screening. (A) Correlation between WGCNA modules and traits. (B) Topological Overlap Matrix (TOM) of all genes analysed, where light colours represent low overlap and progressively darker shades of red indicate higher overlap. (C) Average gene significance of each WGCNA module. (D‐F) Scatter plots of trait‐related modules and gene significance for other significant modules. (G) Box plot of changes in the proportions of various immune cells before and after donor mobilisation predicted by CIBERSORT. (H) Box plot of changes in the proportions of various immune cells before and after donor mobilisation predicted by MCPCOUNTER. (I) Box plot of changes in the proportions of various immune cells before and after donor mobilisation predicted by QuantiSEQ. Statistical methods: (A‐F) Pearson correlation. (G‐I) Wilcoxon rank‐sum test. The stars refer to the p‐value markers. *** = p < 0.001, ** = p < 0.01, * = p < 0.05, ns = p > 0.05. [file CPR-9999-e70213-s006.pdf]

# A

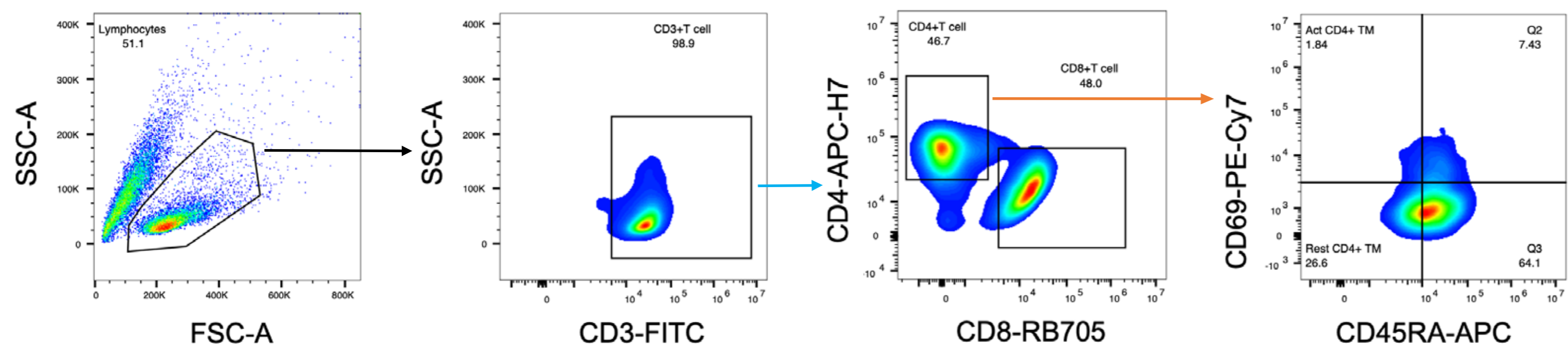

# B

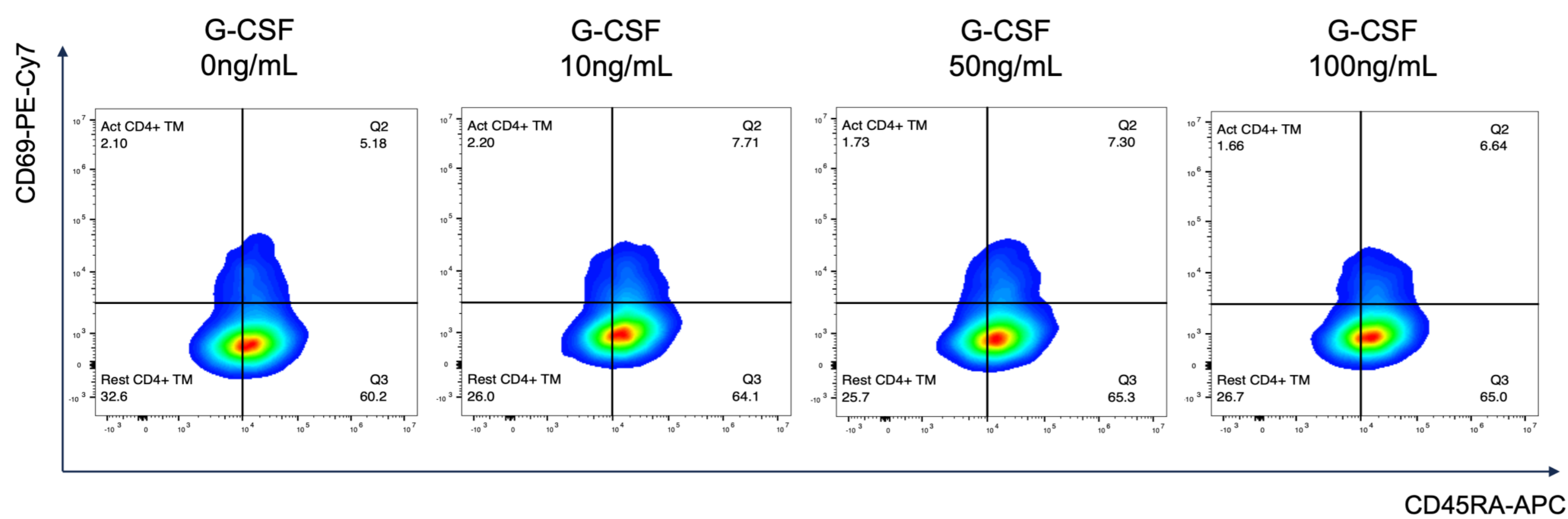

# D

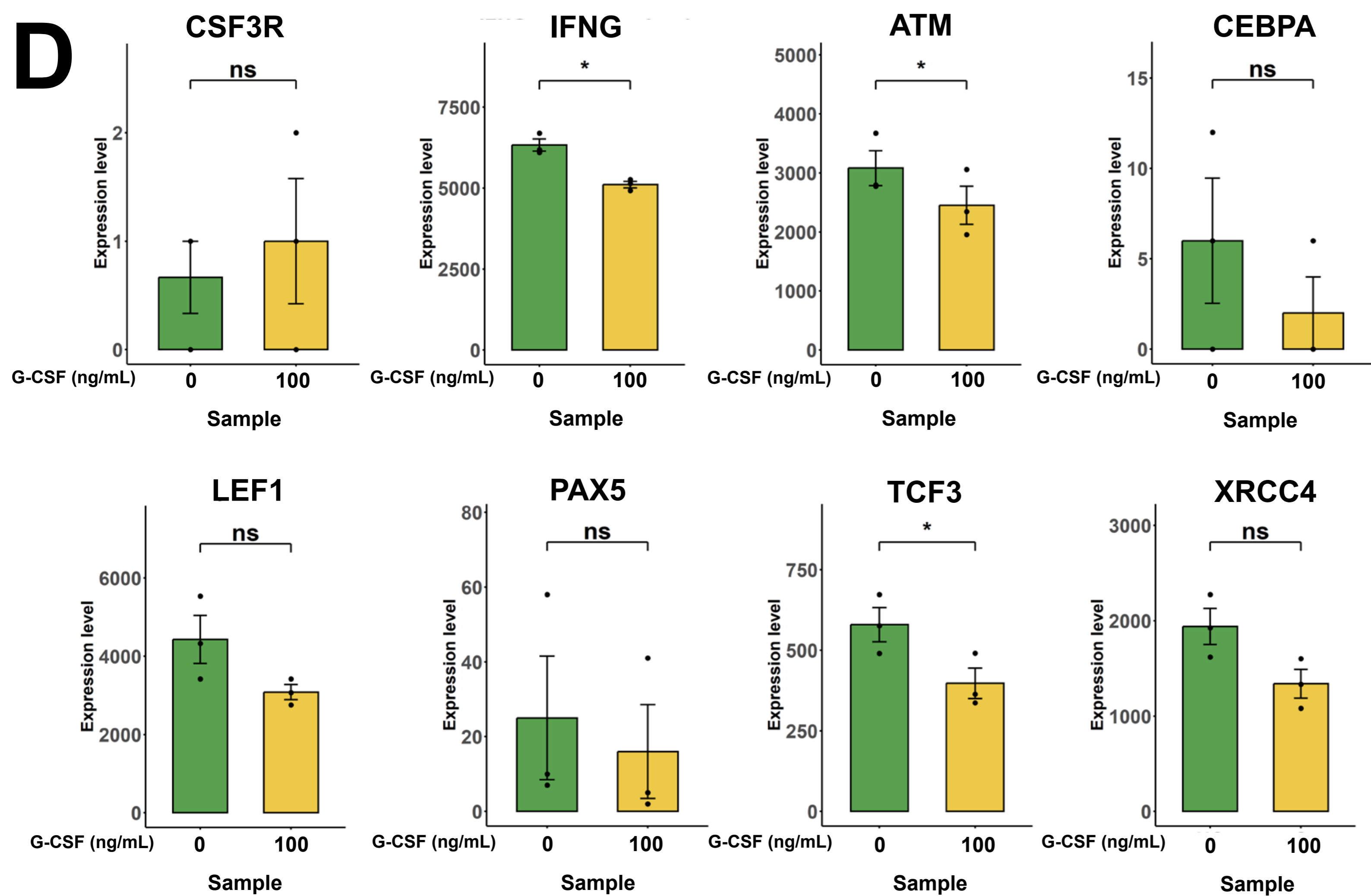

# C

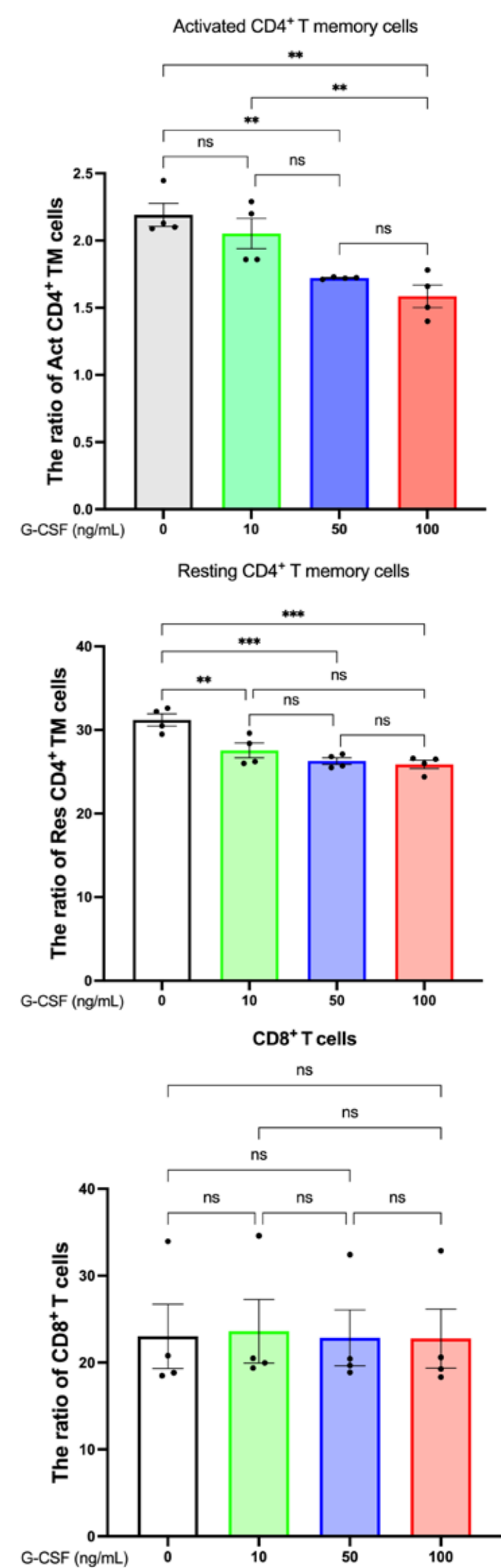

# E

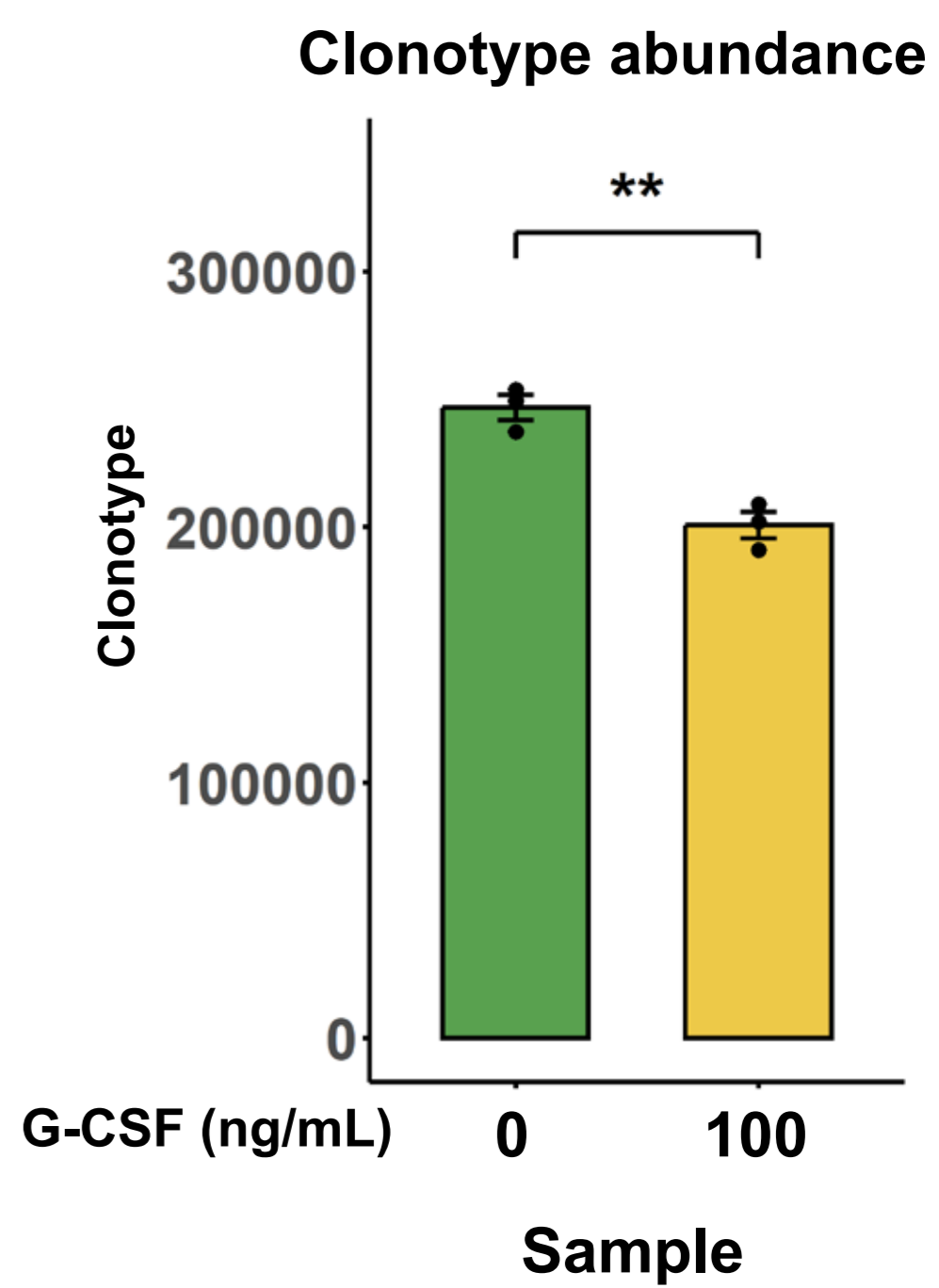

Supplement: Supplementary file 3 — Figure S2: G‐CSF treatment affects the distribution of T cell subsets and TCR clonal diversity in human CD3+ T cells in vitro. (A) Gating strategies for the cell populations. (B) Flow cytometry results of human CD3+ T cells treated with G‐CSF in vitro. (C) Proportions of activated, resting CD4+ T cells and CD8+ T cells from human CD3+ T cells treated with G‐CSF (n = 4). (D) RNA‐seq results of G‐CSF‐related genes in human CD3+ T cells treated with G‐CSF in vitro (n = 3). (E) The TCR‐seq results of human CD3+ T cells treated with G‐CSF in vitro (n = 3). Statistical methods: (C) Wilcoxon rank‐sum test. (D‐E) Paired sample t‐test. The stars refer to the p‐value markers. *** = p < 0.001, ** = p < 0.01, * = p < 0.05, ns = p > 0.05. [file CPR-9999-e70213-s008.pdf]

## A

### Top20 CDR3 V-D-J Heatmap TCRB

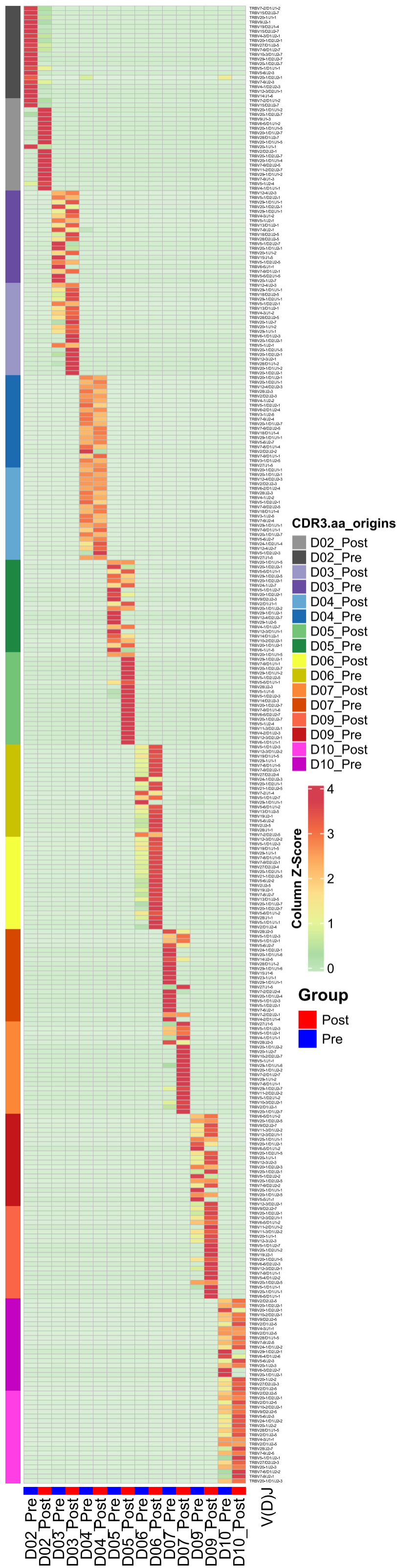

## B

### Top20 CDR3 V-D-J Heatmap TCRD

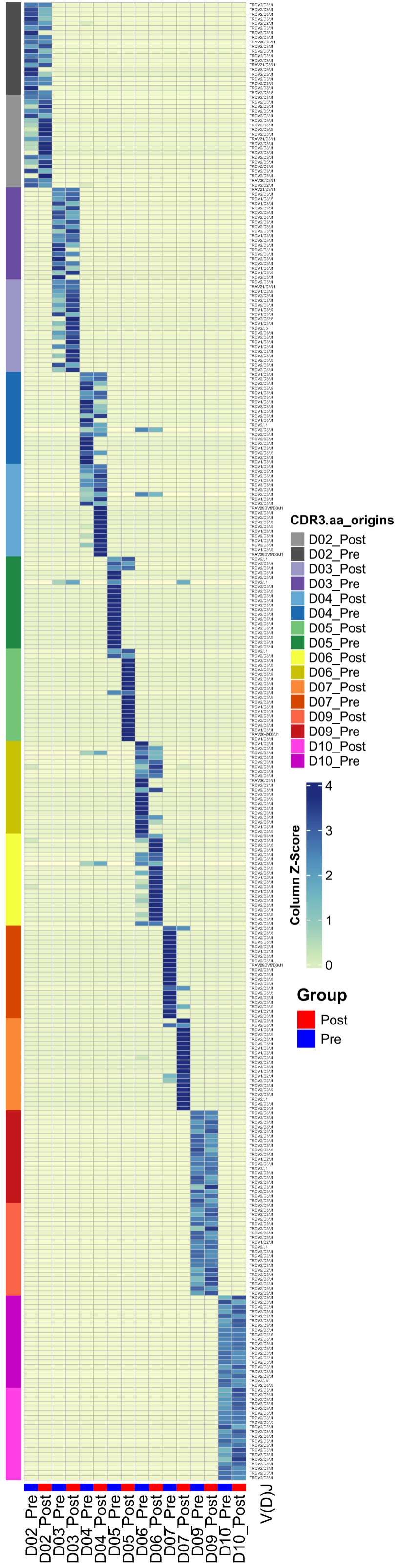

Supplement: Supplementary file 7 — Figure S6: G‐CSF treatment induces changes in high‐frequency donor TCR clones. (A) Expression of the top 20 high‐frequency TCR Vβ clones in different donors before and after G‐CSF mobilisation. (B) Expression of the top 20 high‐frequency TCR Vδ clones in different donors before and after G‐CSF mobilisation. [file CPR-9999-e70213-s005.pdf]

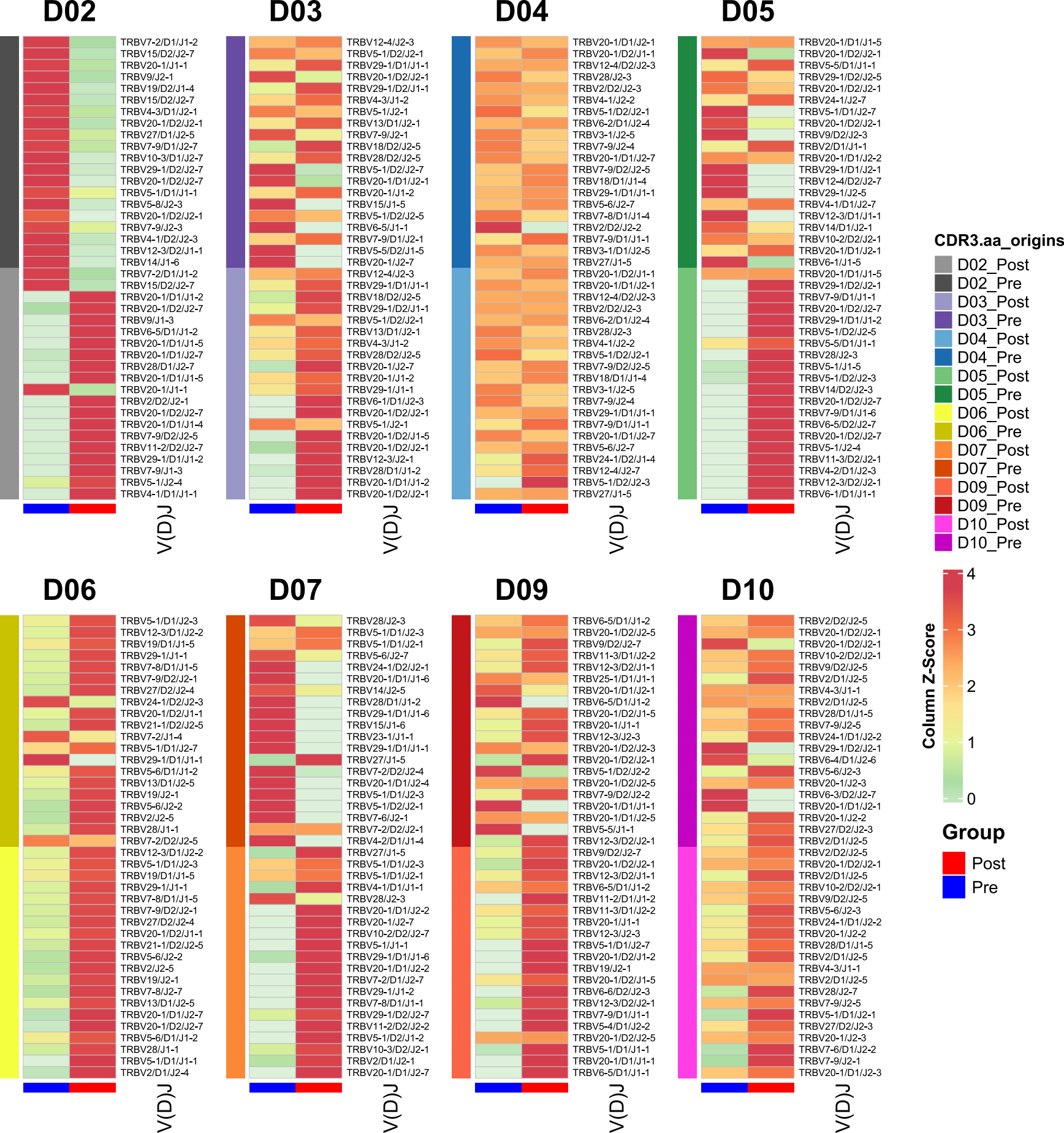

Supplement: Supplementary file 8 — Figure S7: Differences in the top 20 high‐frequency TCR Vβ clones in different donors before and after G‐CSF mobilisation. [file CPR-9999-e70213-s002.pdf]

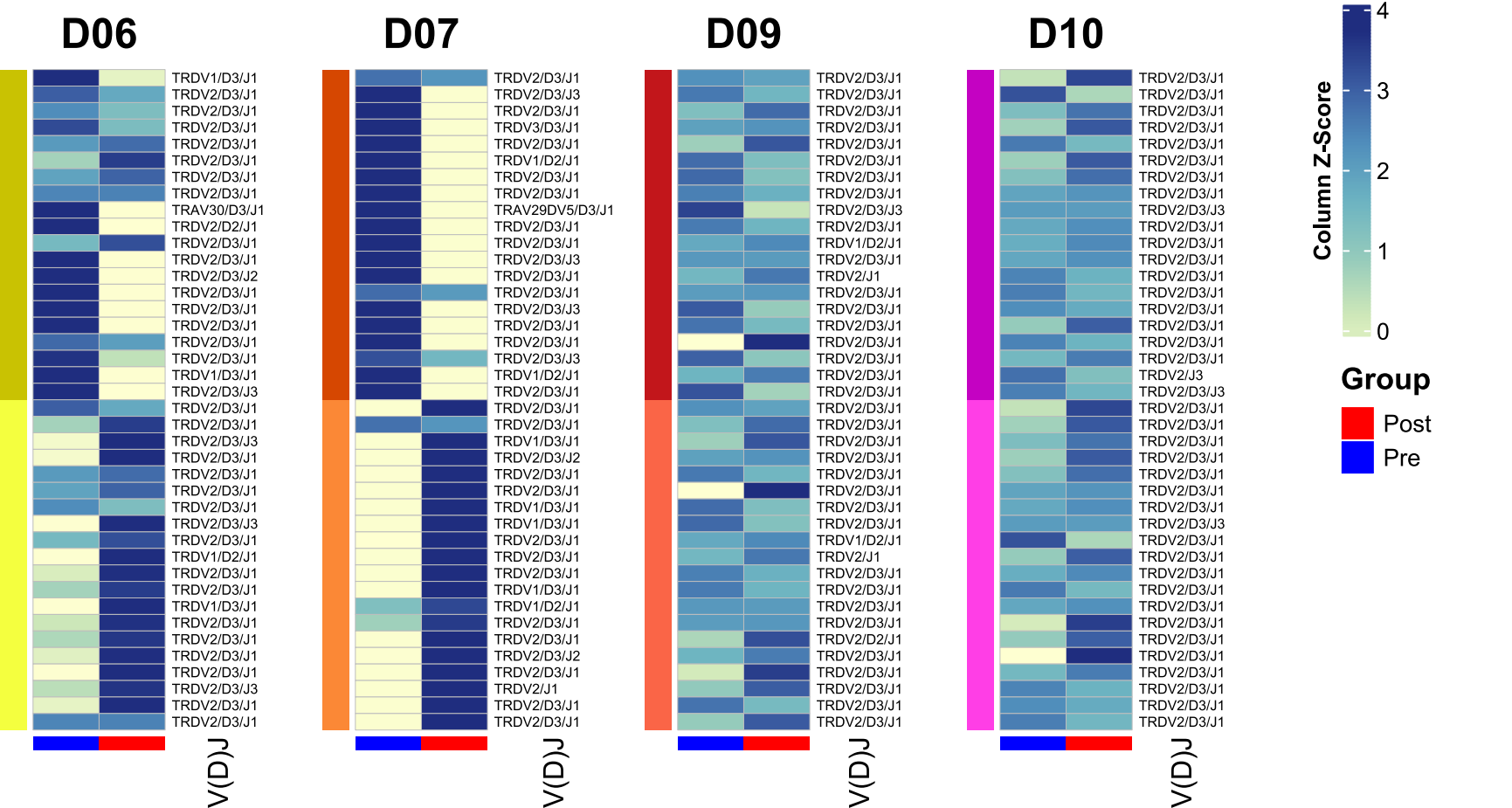

Supplement: Supplementary file 9 — Figure S8: Differences in the top 20 high‐frequency TCR Vδ clones in different donors before and after G‐CSF mobilisation. [file CPR-9999-e70213-s001.pdf]

A

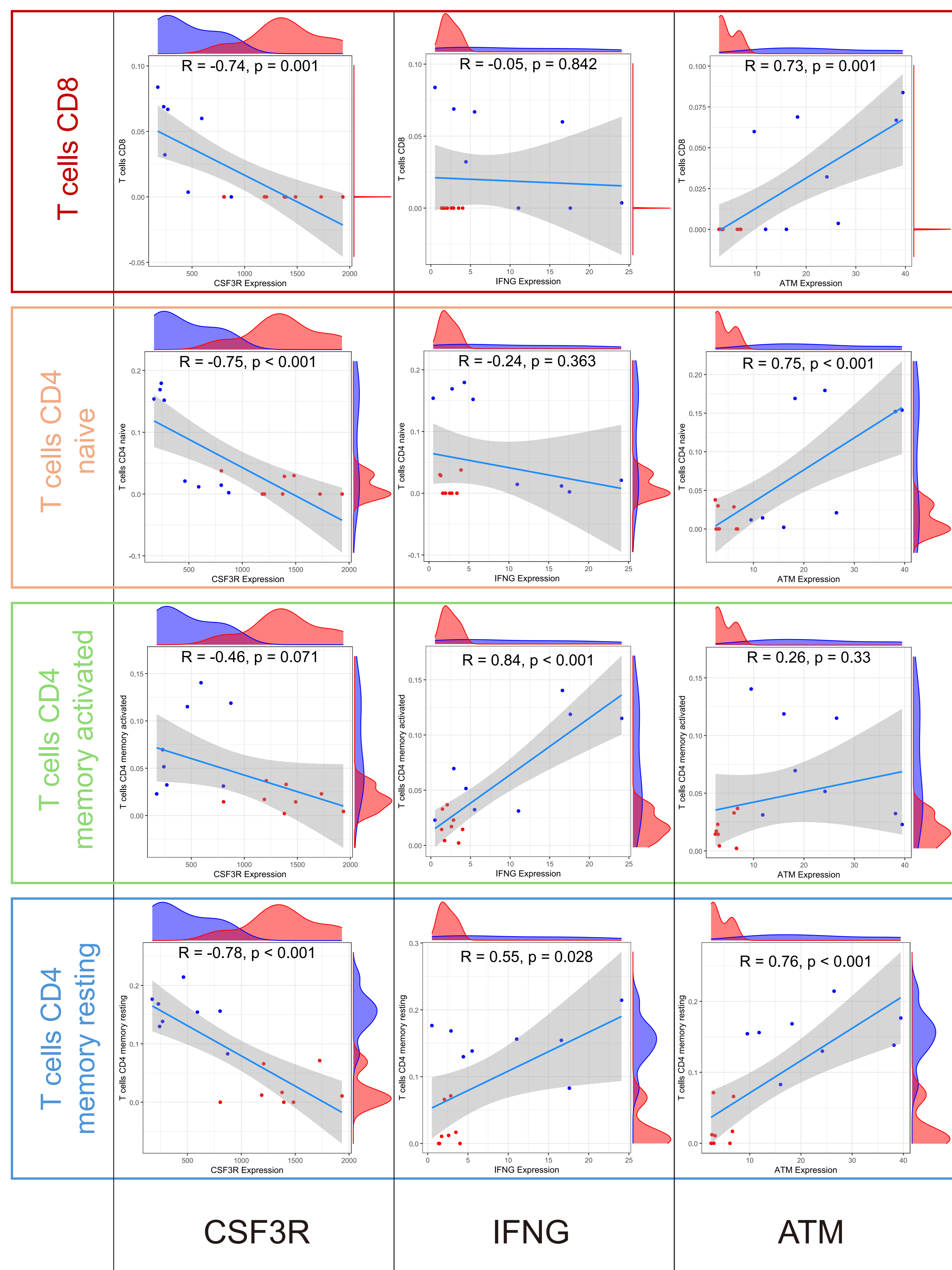

B

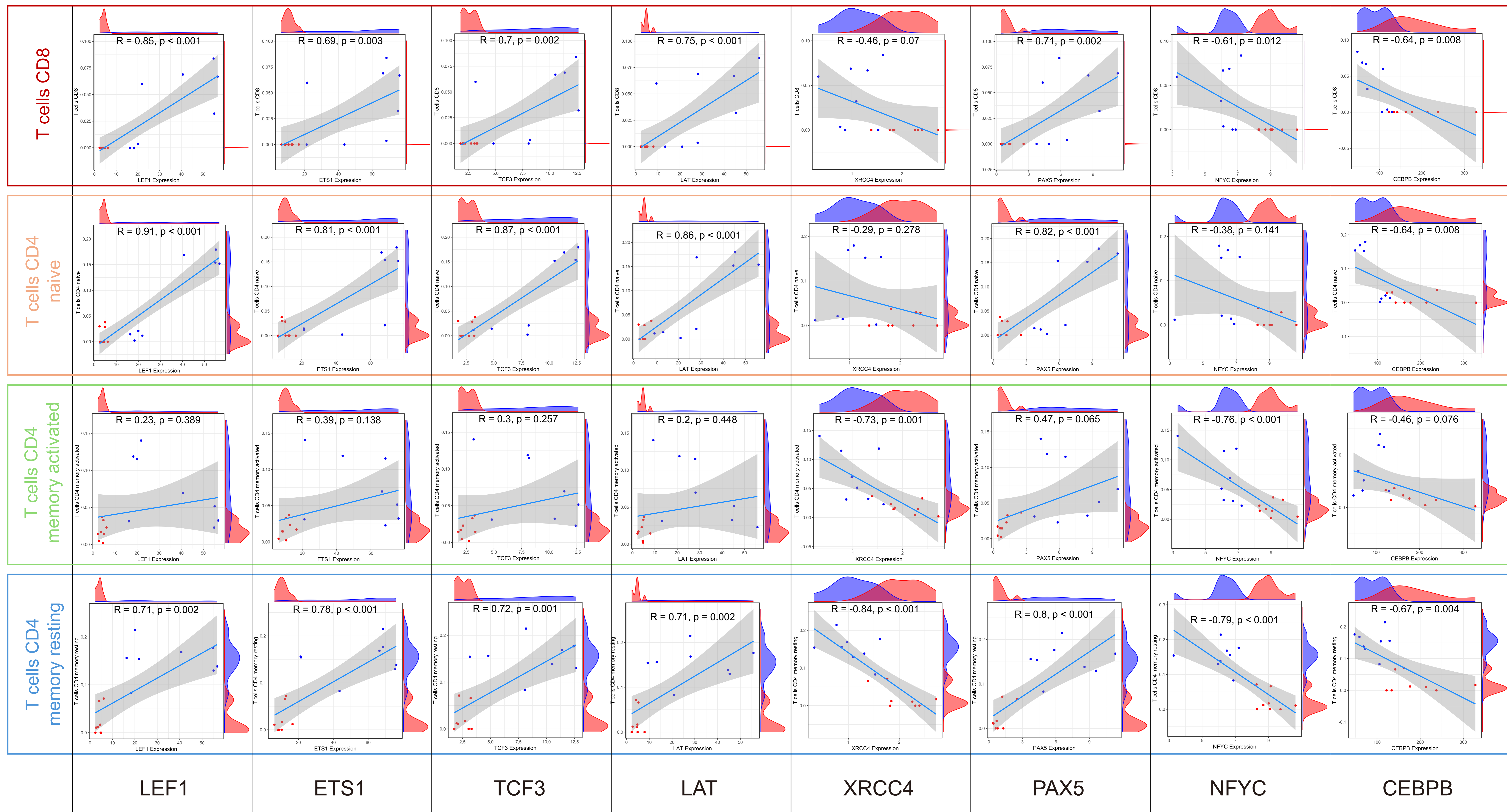

Supplement: Supplementary file 11 — Figure S10: Correlation analysis of the key differentially expressed genes and proportions of the various cell populations predicted by CIBERSORT before and after G‐CSF mobilisation. (A) Correlation between the proportion of resting CD4+ memory T cells, activated CD4+ memory T cells, naive CD4+ T cells, and CD8+ T cells and the expression of CSF3R, IFNG and ATM. (B) Correlation between the proportion of resting CD4+ memory T cells, activated CD4+ memory T cells, naive CD4+ T cells, and CD8+ T cells and the expression of key TCR rearrangement‐related genes. Statistical methods: Pearson correlation. [file CPR-9999-e70213-s011.pdf]
