# Supplementary material for: Impact of G‐CSF on Donor TCR Clonal Diversity and T Cell Function During Donor HSC Mobilisation
Source: Cell Prolif. 2026 Apr 16:e70213. Online ahead of print. doi: 10.1111/cpr.70213 (PMC13325648; doi:10.1111/cpr.70213)
Supplement: Supplementary file 5 — Figure S4: Changes in high‐frequency usage of the TCR Vδ chain V, J, and D gene segments in donors before and after G‐CSF treatment. (A) V region gene changes. (B) J region gene changes. (C) D region gene changes. [file CPR-9999-e70213-s009.pdf]

TCR δ chain

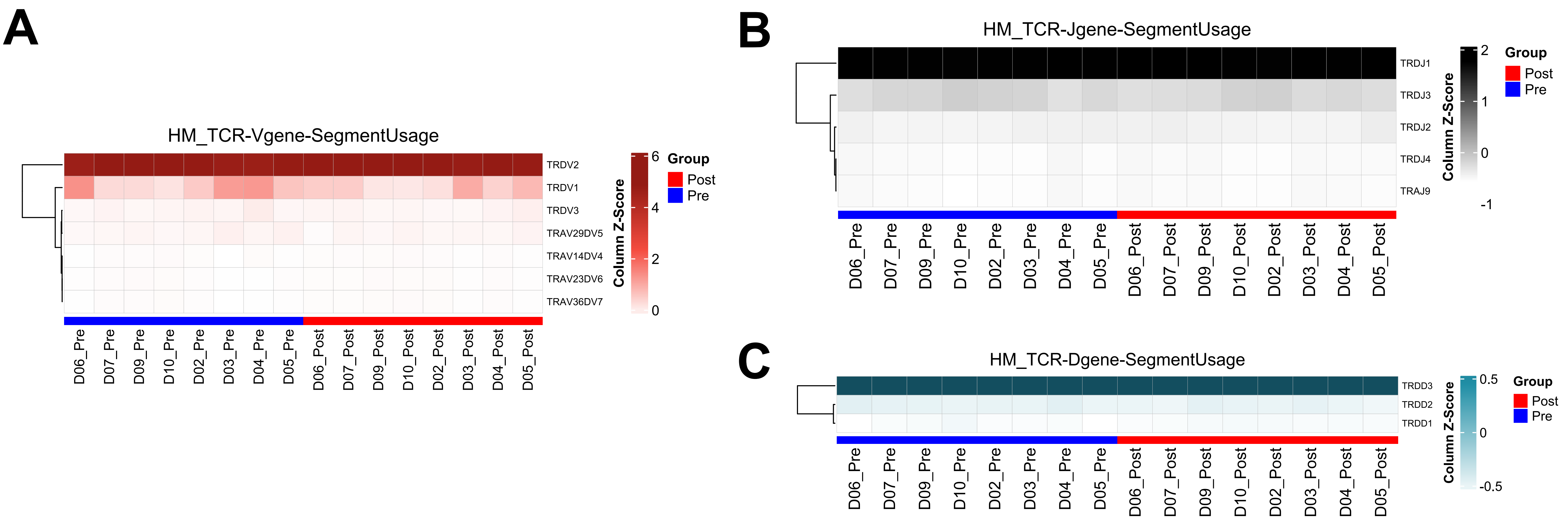
A
HM_TCR-Vgene-SegmentUsage
TRDV2
TRDV1
TRDV3
TRAV29DV5
TRAV14DV4
TRAV23DV6
TRAV36DV7
Column Z-Score
6
4
2
0
Group
Post
Pre
D06_Pre
D07_Pre
D09_Pre
D10_Pre
D02_Pre
D03_Pre
D04_Pre
D05_Pre
D06_Post
D07_Post
D09_Post
D10_Post
D02_Post
D03_Post
D04_Post
D05_Post
B
HM_TCR-Jgene-SegmentUsage
TRDJ1
TRDJ3
TRDJ2
TRDJ4
TRAJ9
Column Z-Score
2
1
0
-1
Group
Post
Pre
D06_Pre
D07_Pre
D09_Pre
D10_Pre
D02_Pre
D03_Pre
D04_Pre
D05_Pre
D06_Post
D07_Post
D09_Post
D10_Post
D02_Post
D03_Post
D04_Post
D05_Post
C
HM_TCR-Dgene-SegmentUsage
TRDD3
TRDD2
TRDD1
Column Z-Score
0.5
0
-0.5
Group
Post
Pre
D06_Pre
D07_Pre
D09_Pre
D10_Pre
D02_Pre
D03_Pre
D04_Pre
D05_Pre
D06_Post
D07_Post
D09_Post
D10_Post
D02_Post
D03_Post
D04_Post
D05_Post
