# Supplementary material for: Impact of G‐CSF on Donor TCR Clonal Diversity and T Cell Function During Donor HSC Mobilisation
Source: Cell Prolif. 2026 Apr 16:e70213. Online ahead of print. doi: 10.1111/cpr.70213 (PMC13325648; doi:10.1111/cpr.70213)
Supplement: Supplementary file 6 — Figure S5: Mathematical analysis showing the decline in TCR diversity in donors before and after G‐CSF mobilisation. (A) D50 statistical analysis of donor TCR Vβ chain clones before and after G‐CSF mobilisation. (B) Chao1 statistical analysis of donor TCR Vβ chain clones before and after G‐CSF mobilisation. (C) D50 statistical analysis of donor TCR Vδ chain clones before and after G‐CSF mobilisation. (D) Chao1 statistical analysis of donor TCR Vδ chain clones before and after G‐CSF mobilisation. (E) Clone index of donor TCR Vβ chain clones before and after G‐CSF mobilisation. (F) Clone index of donor TCR Vδ chain clones before and after G‐CSF mobilisation. Statistical methods: Wilcoxon rank‐sum test. [file CPR-9999-e70213-s007.pdf]

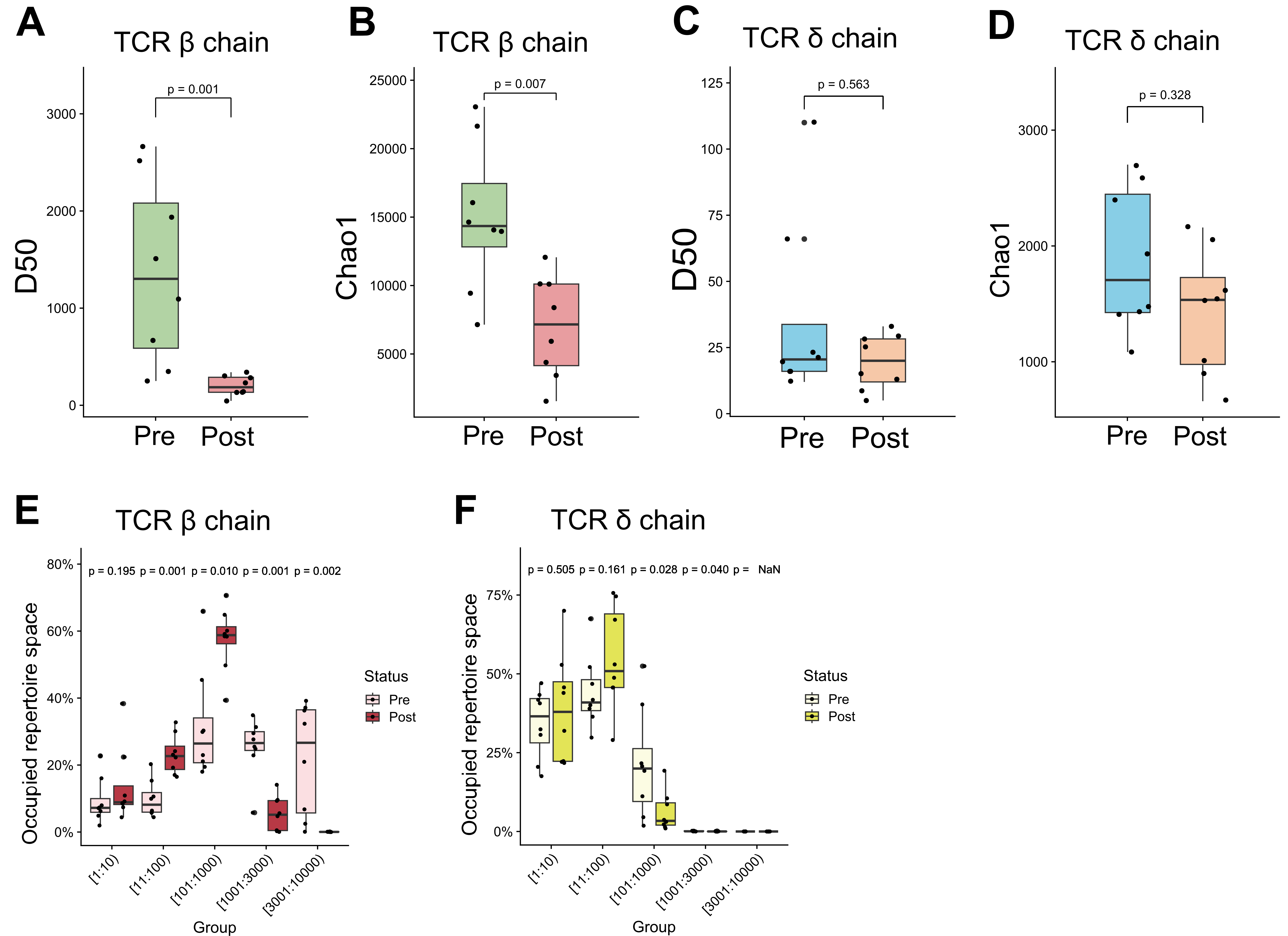

A
TCR β chain
D50
p = 0.001
Pre
Post
B
TCR β chain
Chao1
p = 0.007
Pre
Post
C
TCR δ chain
D50
p = 0.563
Pre
Post
D
TCR δ chain
Chao1
p = 0.328
Pre
Post
E
TCR β chain
Occupied repertoire space
p = 0.195 p = 0.001 p = 0.010 p = 0.001 p = 0.002
[1:10) [11:100) [101:1000) [1001:3000) [3001:10000)
Group
Status
Pre
Post
F
TCR δ chain
Occupied repertoire space
p = 0.505 p = 0.161 p = 0.028 p = 0.040 p = NaN
[1:10) [11:100) [101:1000) [1001:3000) [3001:10000)
Group
Status
Pre
Post
