# Supplementary material for: Impact of G‐CSF on Donor TCR Clonal Diversity and T Cell Function During Donor HSC Mobilisation
Source: Cell Prolif. 2026 Apr 16:e70213. Online ahead of print. doi: 10.1111/cpr.70213 (PMC13325648; doi:10.1111/cpr.70213)
Supplement: Supplementary file 10 — Figure S9: ECDF curves for antigen‐related and background TCR clones before and after G‐CSF mobilisation. (A) ECDF plot for the top 200 clonotypes. (B) ECDF plot for the top 300 clonotypes. (C) ECDF plot for the top 500 clonotypes. [file CPR-9999-e70213-s003.pdf]

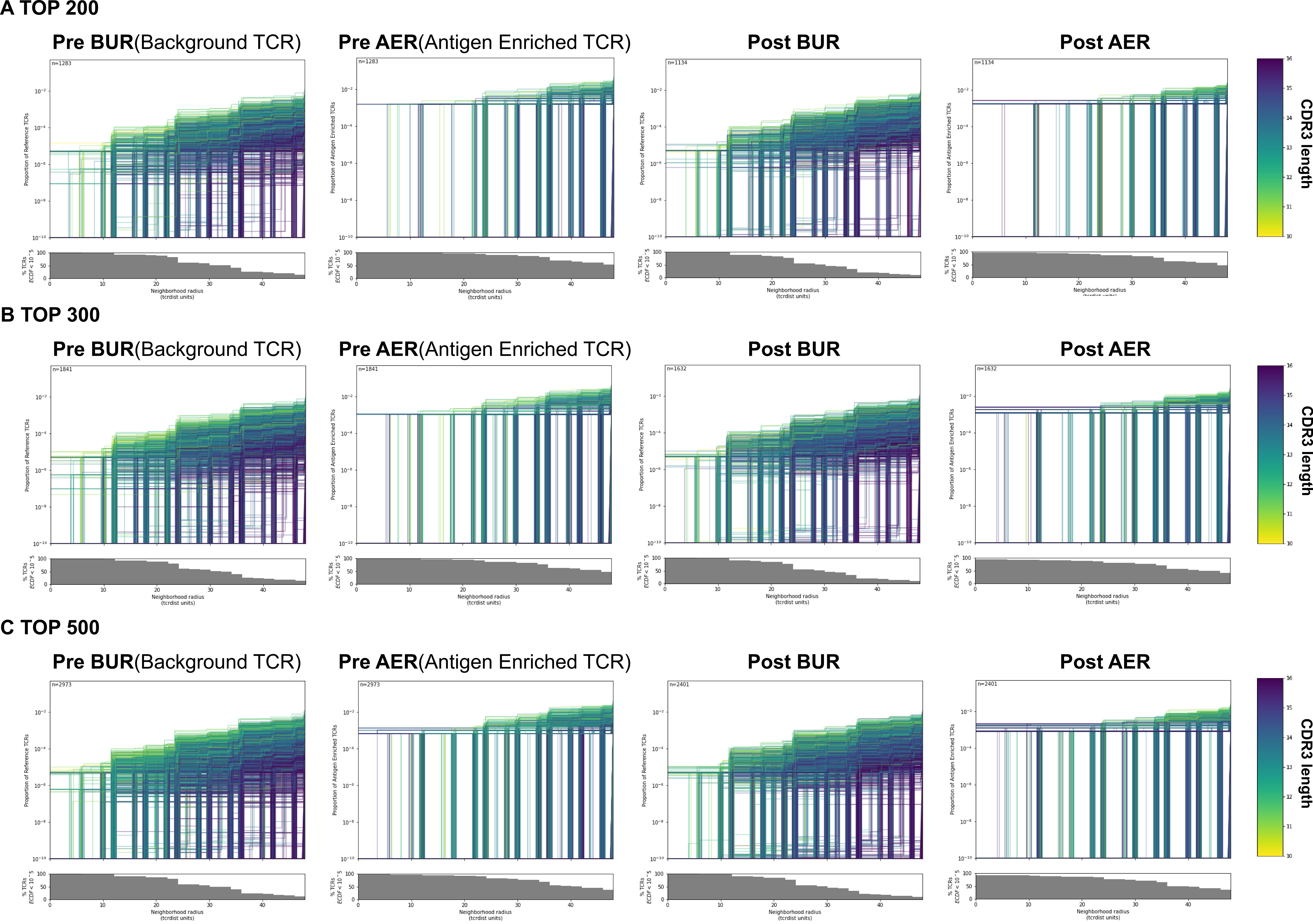
A TOP 200
Pre BUR(Background TCR)
Pre AER(Antigen Enriched TCR)
Post BUR
Post AER
n=1283
n=1283
n=1134
n=1134
Proportion of Reference TCRs
Proportion of Antigen Enriched TCRs
% TCRs ECDF < 10^-5
Neighborhood radius
(tcrdist units)
CDR3 length
B TOP 300
Pre BUR(Background TCR)
Pre AER(Antigen Enriched TCR)
Post BUR
Post AER
n=1841
n=1841
n=1632
n=1632
CDR3 length
C TOP 500
Pre BUR(Background TCR)
Pre AER(Antigen Enriched TCR)
Post BUR
Post AER
n=2973
n=2973
n=2401
n=2401
CDR3 length
